# A comprehensive review of the botany, phytochemistry, pharmacology, and toxicology of Murrayae Folium et Cacumen

**DOI:** 10.3389/fphar.2024.1337161

**Published:** 2024-03-28

**Authors:** Yue Qi, Lin Wang, Na Wang, Siyi Wang, Xu Zhu, Tie Zhao, Qinghua Jiang

**Affiliations:** ^1^ Department of Obstetrics and Gynecology, Shengjing Hospital of China Medical University, Shenyang, China; ^2^ School of Pharmacy, China Medical University, Shenyang, China; ^3^ Department of Pharmacy, The People’s Hospital of Liaoning Province, Shenyang, China; ^4^ The 1st Clinical Department, China Medical University, Shenyang, China; ^5^ Department of Pharmacy, Shengjing Hospital of China Medical University, Shenyang, China

**Keywords:** Murrayae Folium et Cacumen, *Murraya paniculata*, *Murraya exotica*, phytochemistry, pharmacology, toxicology

## Abstract

**Ethnopharmacological relevance:** Murrayae Folium et Cacumen (MFC) is a plant considered to be a traditional Chinese medicine with culinary value as well. The dry leaves and twigs of *Murraya paniculata* and *M. exotica* are used to treat stomach aches, rheumatism, toothaches, swelling, and insect and snake bites. They are also used to prepare spicy chicken dishes.

**Aim of the review:** This review comprehensively summarizes the available information on the botanical characterization, phytochemistry, pharmacological activities, pharmacodynamics, pharmacokinetics, and toxicity of MFC.

**Methods:** Relevant scientific literature up to August 2023 was included in the study. Chinese and English studies on MFC were collected from databases, including PubMed, Elsevier, Web of Science, Springer, Science Direct, Wiley, ACS, and CNKI (Chinese). Doctoral and Master’s dissertations were also included.

**Results:** In total, 720 compounds have been identified and reported in the literature, including flavonoids, coumarins, alkaloids, sterols, phenylpropenols, organic acids, spirocyclopentenones, and volatile oils. Flavonoids and coumarins are the two most important bioactive compounds responsible for these pharmacological activities. MFC has anti-inflammatory, anti-bacterial, anti-microbial, anti-diabetic, anti-tumor, anti-oxidant, anti-depressant, potential anti-Alzheimer’s disease, chondroprotective, and analgesic properties. The pharmacological effects include interrupting the STAT3/NF-κB/COX-2 and EGFR signaling pathways, downregulating EpCAM expression, inhibiting NF-κB and ERK signals, inhibiting the EP/cAMP/PKA signaling pathway and miR-29a/Wnt/β-catenin signaling activity, and upregulating Foxo3a expression.

**Conclusion:** This review demonstrates that the chemical constituents, pharmacological activities, pharmacodynamics, pharmacokinetics, and toxicity of MFC support its use in traditional Chinese botanical medicines. MFC contains a wide range of chemical compounds. Flavonoids and coumarins promote strong pharmacological activity and, are low-toxicity natural phytomedicines that are widely used in medicine, food, ornamentation, and cosmetics, making MFC a promising compound for development and use in the treatment of several medical conditions.

## 1 Introduction

The genus *Murraya* comprises 21 accepted species (http://www.worldfloraonline.org). *Murraya paniculata* (L.) Jack and *M. exotica* L. (Rutaceae) are the most widely used *Murraya* species listed in the Chinese Pharmacopoeia (Chinese Pharmacopoeia Commission, 2020). Murrayae Folium et Cacumen (MFC), a traditional Chinese medicine, consists of dry leaves and twigs of *M. paniculata* and *M. exotica* (Lu et al., 2021a). It promotes qi, relieves pain, activates blood, and removes blood stasis and is used mainly to treat stomach pain, rheumatism, arthralgia, toothache, tumefaction, and snakebites (Chinese Pharmacopoeia Commission, 2020). MFC is the main ingredient in Sanjiu Weitai granules, a well-known Chinese-patented medicine used for the treatment of gastric conditions (Lu et al., 2021b). In addition, it is also used in the *Baihu Dan* in the *Jingyue Complete Book* to treat swelling of the head, face, limbs and eyes, and *Zhitong Jing* in the *Compilation of Chinese Medicine* to promote circulation and relieve pain.

The leaves of *Murraya paniculata* are used as a spice by the people of India, Southeast Asia, Pakistan, and Malaysia in a variety of food preparations. Malaysians typically use *M. paniculata* leaves to prepare soups, fish, and meat. *M. paniculata* has also been used to prepare spicy chicken dishes in popular fast-food restaurants (Ng et al., 2012; Saqib et al., 2015). In ancient China, India, and Indonesia, *M. paniculata* was used as a botanical medicine for numerous healthcare purposes. In the Chinese Pharmacopoeia, *M. paniculata* is reported to have analgesic effects and the potential to treat microbial infections and inflammatory diseases (Zhang et al., 2011). The ground stem bark of *M. paniculata* is used as an antidote for snake bites, whereas the ground roots are used to treat body pain. The leaves are irritating and astringent and are used by the Indonesian community to relieve diarrhea and dysentery (Saqib et al., 2015). *M. paniculata* has also been used to treat coughs, hysteria, and rheumatism (Gautam et al., 2012a). It is used to treat snake bites and as a detergent for other types of bites. The roots and bark are chewed and rubbed against the skin to treat pain. Crushed leaves are applied to fresh wounds and as a remedy for alcohol-related fluid retention. *M. paniculata* can be used to treat toothaches, stomach-aches, and gout. It is also used for abortion and to treat venereal diseases (Kinoshita and Firman, 1996a; Rahman et al., 2010). Terpenoid volatile oils extracted from the flowers of *M. paniculata* are used in the cosmetic industry (Rout et al., 2007; Rehman et al., 2013).

*Murraya exotica* is a dwarf tree or evergreen shrub commonly cultivated as an ornamental plant in many tropical and subtropical areas because of its glossy green leaves and clusters of fragrant white flowers (Sharma and Arora, 2015). It has also been used for the treatment of analgesia, anesthesia, abdominal pain, and rheumatism. The leaves of *M. exotica* are rich in coumarins, which exhibit anti-oxidant, anti-tumor, anti-mycobacterial, anti-fungal, anti-viral, and anti-inflammatory properties. The roots of *M. exotica* are rich in coumarins and alkaloids, such as paniculidines (A−F) (He et al., 2017).

Previous phytochemical studies on MFC (*M. paniculata* and *M. exotica*) have indicated the main bioactive compounds as coumarins, flavonoids, alkaloids, and volatile oils, of which the alkaloids are mainly present in the roots of plants. Pharmacological studies have demonstrated that MFC possesses anti-inflammatory, analgesic, anti-bacterial, anti-oxidant, and insecticidal properties (Lu et al., 2021a). To date, there have been no systematic reports on MFC. Therefore, it is necessary to summarize the phytochemistry, pharmacology, pharmacodynamics, pharmacokinetics, and toxicology of MFC to guide clinical use in the Chinese Pharmacopoeia.

## 2 Botanical characterization

### 2.1 Plant description

**FIGURE 1:**
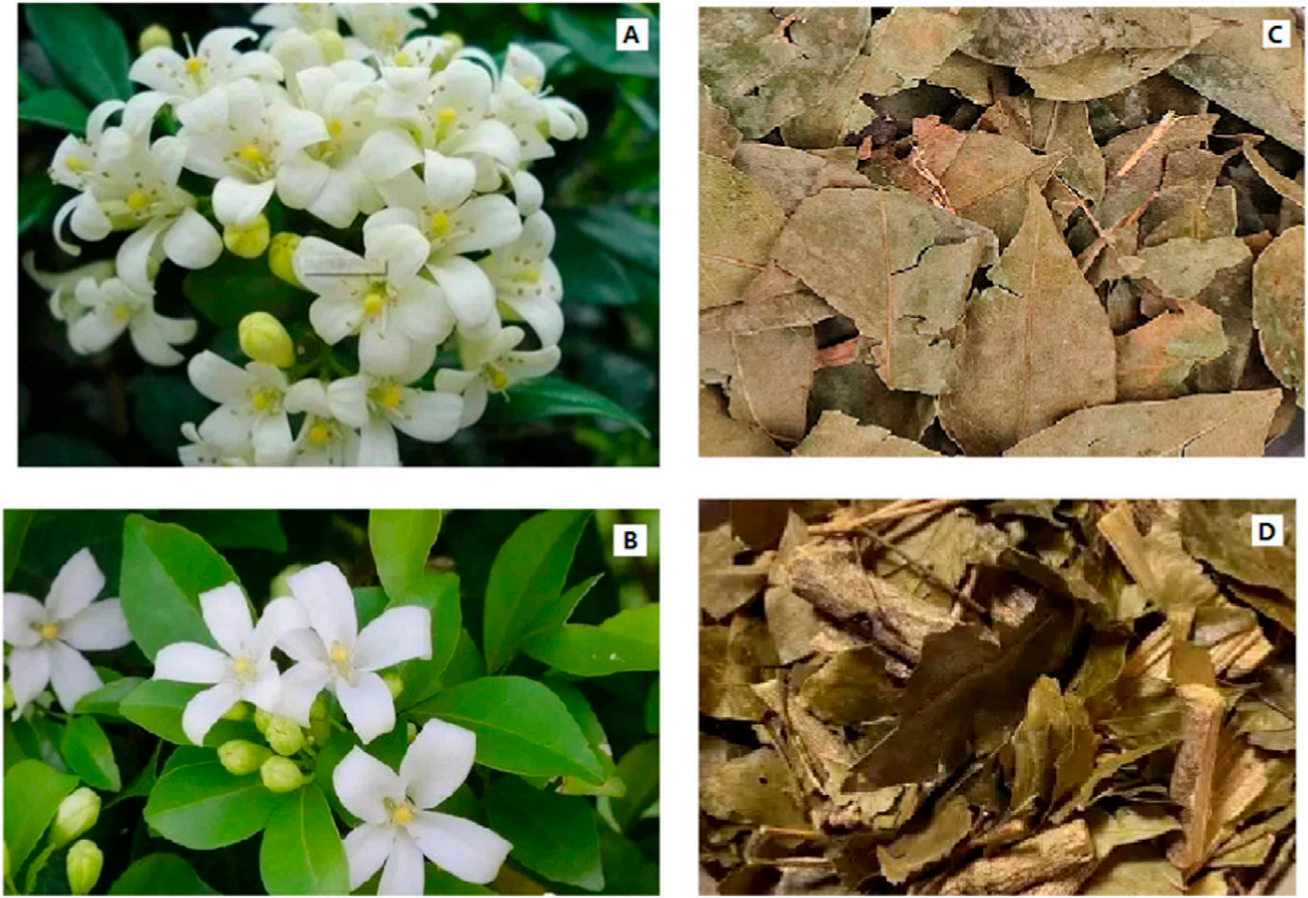
**(A)** *M*. *paniculata*; **(B)** *M*. *exotica*; **(C, D)** Murrayae Folium et Cacumen.

*Murraya paniculata* are 1.8–12 m tall shrubs or trees. Older branchlets are grayish-white to pale yellowish-gray. The leaves are 2–5 foliolate, and leaflet blades are mostly suborbicular to ovate to elliptical, margin entire or crenulate, and apex rounded to acuminate (http://www.worldfloraonline.org) (Figure 1A).

*Murraya exotica* are 2–8 m tall shrubs or trees. Older branchlets are grayish-white to pale yellowish-gray. The leaves are 3–7 foliolate, petiolules are rather short, leaflet blades are elliptic-obovate or obovate, and margins are entire, apex rounded, or obtuse (http://www.worldfloraonline.org) (Figure 1B).

### 2.2 Vernacular names

Vernacular names—in other words, local, common, or non-Latin names for a plant or animal—are derived from common native languages and are distinct from binomial nomenclature. The name is derived from the plant’s morphology, habits, habitats, organoleptic properties, and therapeutic uses (Hossain et al., 2021). The vernacular names of MFC are listed in Table 1.

**TABLE 1:** Vernacular name of MFC.

Species	Local name	Country	References
*M. paniculata*	Kemuning Putih; orange jasmine	Malaysia	Ng et al. (2012)
*M. paniculata*	Daun kumning	Indonesia	Kinoshit and Firman, (1996a)
*M. paniculata*	Chinese box	America; Canada	Aziz et al. (2010)
*M. paniculata*	Qianlixiang; jiulixiang	China	Zhang et al. (2011)
*M. paniculata*	Orange jasmine; honey bush; kamini	India	Gautam et al. (2012a), Rehman et al. (2013)
*M. paniculata*	Orange jasmine	Pakistan	Rehman et al. (2013)
*M. paniculata*	Bajardante	Nepal	Dosoky et al. (2016)
*M. paniculata*	Nguyet que	Vietnam	Cuong et al. (2014)
*M. paniculata*	Kaew	Thailand	Rodanant et al. (2015)
*M. paniculata*	Orange jasmine	Brazil	Menezes et al. (2017)
*M. exotica*	Jiulixiang; qianlixiang	China	Wu et al. (2010)
*M. exotica*	Marwa	Pakistan	Siddiqua et al. (2023)

## 3 Materials and methods

This review focuses on the research advances in the phytochemical constituents, pharmacological activities, pharmacodynamics, pharmacokinetics, and toxicology of MFC. Related scientific literature up to August 2023 was collected from the following databases: PubMed, Elsevier, Web of Science, Springer, Science Direct, Wiley, ACS, and CNKI (Chinese). Doctoral and Master′s dissertations were also included in the analysis. We used the terms (all fields) “Murrayae Folium et Cacumen,” “*Murraya paniculata*,” “*Murraya exotica*,” and “*Murraya*” and collated all published works from the China Medical University Library. Only data published in English or Chinese were included in the analysis. ChemDraw 20.0 was used to extract the chemical compounds. The PubChem database (https://pubchem.ncbi.nlm.nih.gov) was used to confirm the chemical classifications and structures. World Flora Online (http://www.worldfloraonline.org/) was used to verify the names of the plants.

### 3.1 Inclusion and exclusion criteria

Firstly, duplicate articles, review articles, conference abstracts; non-English and non-Chinese articles were excluded. Further exclusions were duplicate articles and articles unrelated to the topic. Finally, 125 eligible articles were included.

## 4 Phytochemistry

**FIGURE 2:**
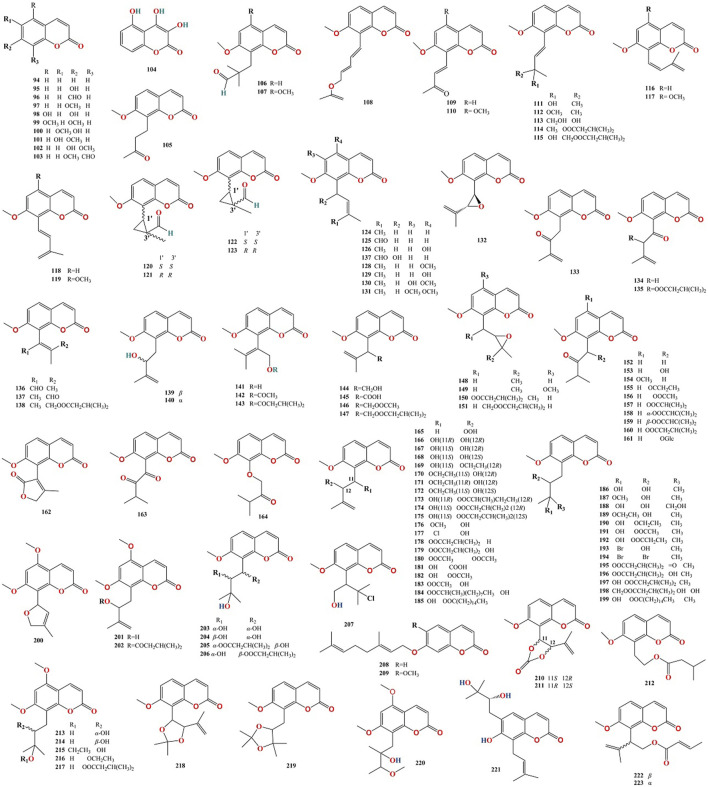
Chemical structures of flavonoids isolated from Murrayae Folium et Cacumen.

**FIGURE 3:**
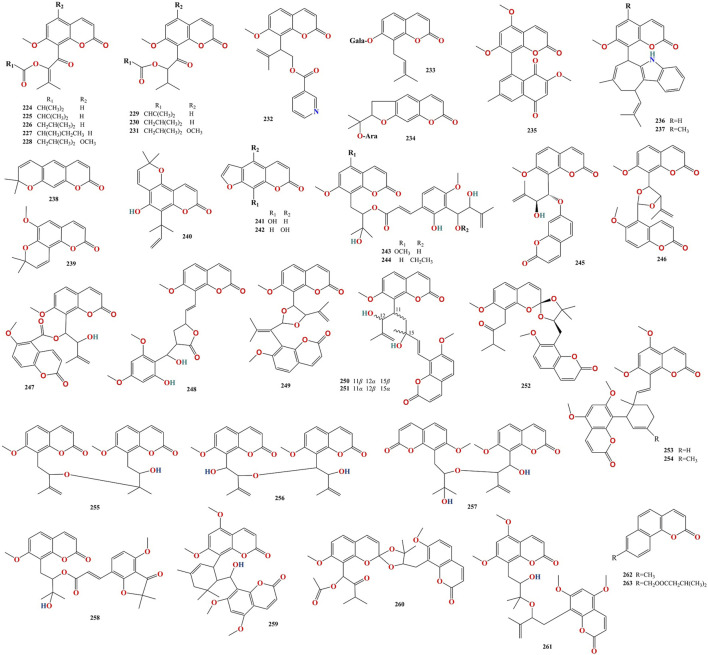
Chemical structures of coumarins isolated from Murrayae Folium et Cacumen.

**FIGURE 4:**
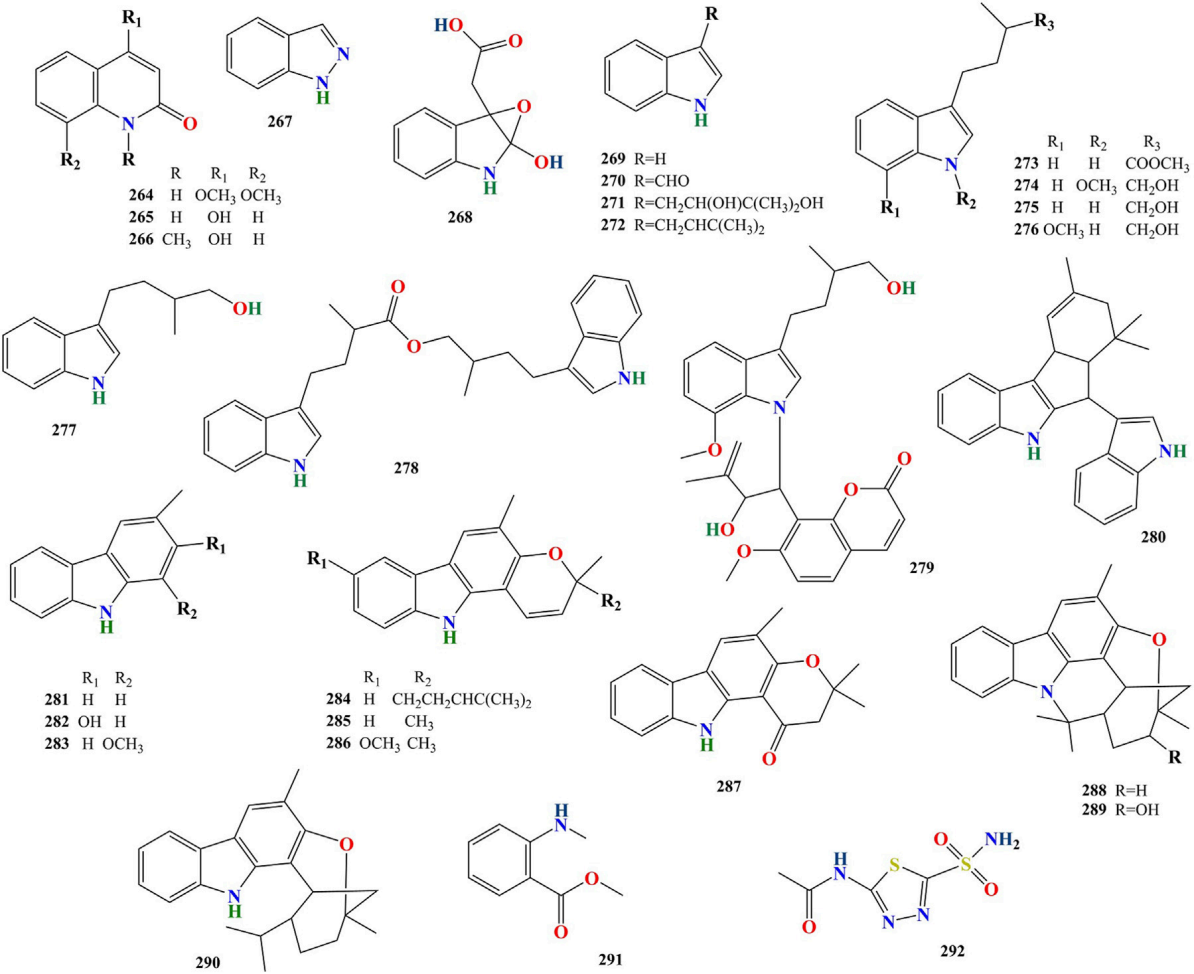
Chemical structures of alkaloids isolated from Murrayae Folium et Cacumen.

**FIGURE 5:**
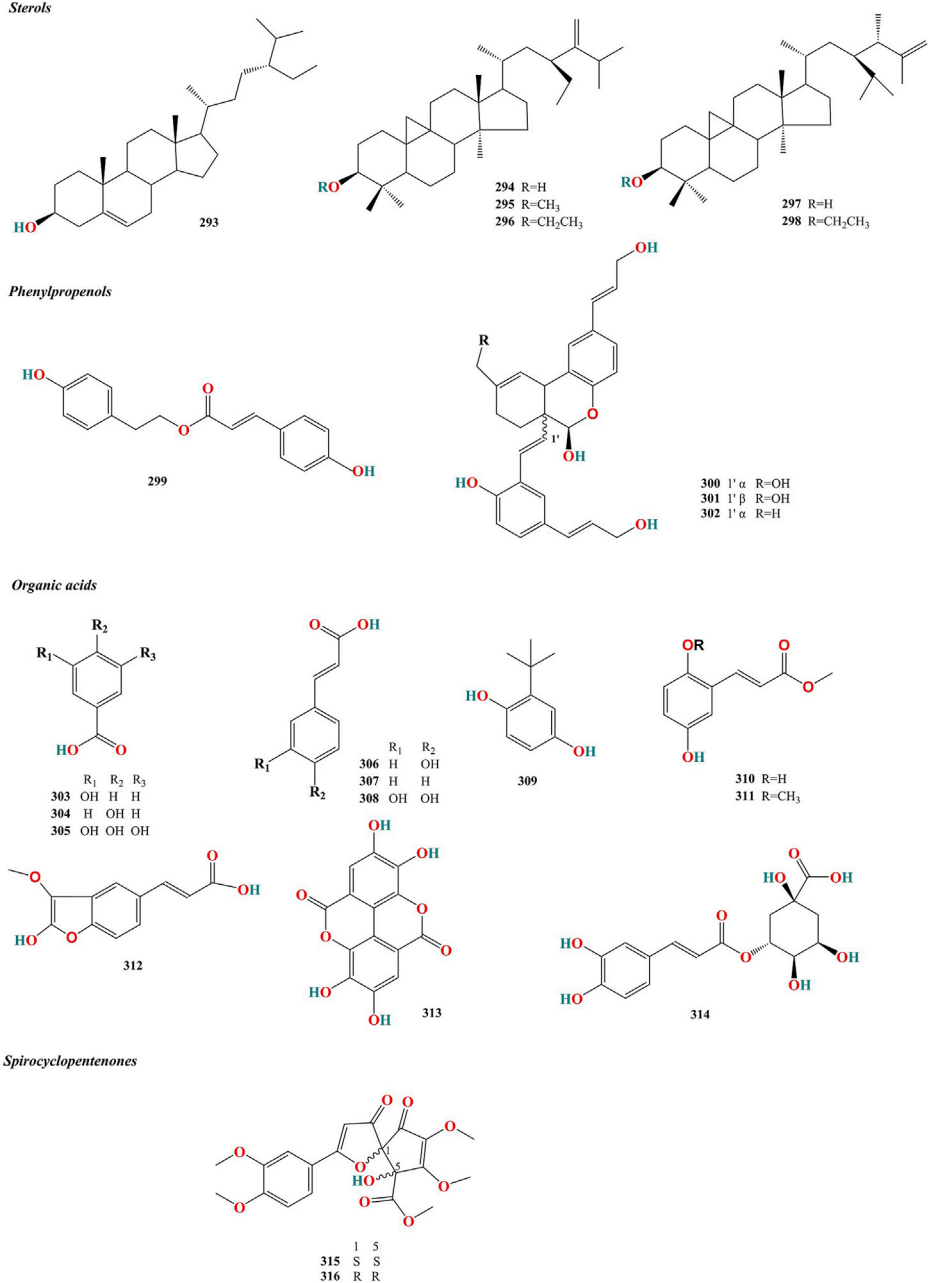
Chemical structures of sterols, pehylpropenols and other constituents isolated from Murrayae Folium et Cacumen.

To date, 316 compounds have been identified in MFC, including flavonoids, coumarins, alkaloids, sterols, phenylpropanoids, organic acids, spirocyclopentenones, and 404 volatile oils. These have been identified using thin layer chromatography (TLC), high-performance liquid chromatography (HPLC), ultra- performance liquid chromatography (UPLC), nuclear magnetic resonance (NMR), ultraviolet (UV), mass spectrometry (MS), NMR-MS, ultra-performance liquid chromatography-electrospray ionization-mass spectrometry (UPLC-ESI-MS), HR-FAB-MS, heteronuclear multiple-bond correlation (HMBC), heteronuclear multiple-quantum correlation (HMQC), and gas chromatography-mass spectrometry (GC-MS) (Kong Y. et al., 1985; Liu et al., 2015a; Kaur et al., 2016; Liang et al., 2020c; Saikia et al., 2021). Flavonoids are the main compounds of *M. paniculata*, whereas coumarins are the main compounds of *M. exotica*. Alkaloids mainly exist in the roots of plants but rarely in the twigs and leaves (Lu et al., 2021a). A detailed list of these chemical compounds and their classes is presented in Figures 2–5, and Tables 2–5 and Supplementary Table S.

**TABLE 2:** Flavonoids isolated from Murrayae Folium et Cacumen.

No.	Name	Species	Formula	Weight	PubChem CID	Extract	Parts of the plant	References
*Flavones*								
1	5,6,7,8,3′,4′,5′-Heptamethoxyflavone	*M. paniculata*	C_22_H_24_O_9_	432.4	72815	Methanol	Leaves	Sangkaew et al. (2020)
		*M. exotica*				Acetone	Branches	Negi et al. (2015)
2	5,6,7,8,3′,4′-Hexamethoxyflavone; nobiletin	*M. paniculata*	C_21_H_22_O_8_	402.4	72344	Methanol	Leaves	Zhang et al. (2012a)
3	5,6,7,3′,4′,5′-Hexamethoxyflavone	*M. paniculata*	C_21_H_22_O_8_	402.4	185670	Methanol	Leaves	Sun et al. (2015)
		*M. paniculata*				Dichloromethane	Peel of fresh ripe fruits	Santos et al. (2009)
		*M. exotica*				Acetone	Branches	Negi et al. (2015)
4	6,7,8,3′,4′,5′-Hexamethoxyflavone	*M. paniculata*	C_21_H_22_O_8_	402.4		Chloroform	Leaves	Kinoshita and Firman (1996b)
5	5,7,8,3′,4′,5′-Hexamethoxyflavone	*M. paniculata*	C_21_H_22_O_8_	402.4		Methanol	Leaves	Zhang et al. (2013b)
		*M. paniculata*				Dichloromethane	Peel of fresh ripe fruits	Santos et al. (2009)
		*M. exotica*				Methanol	Leaves	Zhang et al. (2014)
6	5,6,7,3′,4′-Pentamethoxyflavone; sinensetin	*M. paniculata*	C_20_H_20_O_7_	372.4	145659	Ethanol	Leaves	Li et al. (2018)
		*M. exotica*				Acetone	Branches	Zhang et al. (2013a)
7	5,7,8,3′,4′-Pentamethoxyflavone	*M. paniculata*	C_20_H_20_O_7_	372.4	632135	Methanol	Leaves	Zhang et al. (2013b)
8	5,7,3′,4′,5′-Pentamethoxyflavone	*M. paniculata*	C_20_H_20_O_7_	372.4	493376	Ethanol	Stems, leaves	Wu et al. (2015)
		*M. exotica*				Ethanol	Leaves, twigs	Peng et al. (2010)
9	5,7,3′,4′-Tetramethoxyflavone	*M. paniculata*	C_19_H_18_O_6_	342.3	631170	Ethanol	Leaves	Li et al. (2018)
		*M. exotica*				Ethanol	Leaves	Huang et al. (2019)
10	7,3′,4′,5′-Tetramethoxyflavone	*M. paniculata*	C_19_H_18_O_6_	342.3		Ethyl acetate	Leaves	Rodanant et al. (2015)
11	6,8,3′,4′-Tetramethoxyflavone	*M. paniculata*	C_19_H_18_O_6_	342.3		Methanol	Leaves	Wang et al. (2010)
12	7,3′,4′,5′-Tetramethoxyflavone	*M. paniculata*	C_19_H_18_O_6_	342.4		Ethyl acetate	Leaves	Rodanant et al. (2015)
13	5,3′,5′-Trihydroxy-6,7,8,4′-tetramethoxyflavone; gardenin E	*M. paniculata*	C_19_H_18_O_9_	390.3	3084508	Chloroform	Leaves	Kinoshita and Firman (1996b)
14	5, 3′-Dihydroxy-6,7,4′,5′-tetramethoxyflavone	*M. paniculata*	C_19_H_18_O_8_	374.3	183329	Ethanol	Leaves, twigs	Liang et al. (2020c)
15	5,3′-Dihydroxy-7,4′,5′-trimethoxyflavone	*M. paniculata*	C_18_H_16_O_7_	344.3	5496476	Ethanol	Leaves, twigs	Liang et al. (2020c)
16	5,3′-Dihydroxy-7, 8,4′-trimethoxyflavone	*M. paniculata*	C_18_H_16_O_7_	344.3		Ethyl acetate	Leaves, twigs	Li, (2022)
17	5,3′-Dihydroxy-7,4′-dimethoxyflavone	*M. paniculata*	C_17_H_14_O_6_	314.3		Ethanol	Leaves	Shan et al. (2010)
18	5,3′-Dihydroxy-7,8,4′,5′-tetramethoxyflavone	*M. paniculata*	C_19_H_18_O_8_	374.3	44258624	Methanol	Leaves	Zhang et al. (2013b)
19	5,3′-Dihydroxy-6,7,8,4′,5′-pentamethoxyflavone; gardenin C	*M. paniculata*	C_20_H_20_O_9_	404.4	3084507	Chloroform	Leaves	Kinoshita and Firman (1996b)
20	3′,5′-Dihydroxy-5,7,4′-trimethoxyflavone	*M. paniculata*	C_18_H_16_O_7_	344.3	134822350	Ethanol	Leaves	Li et al. (2018)
21	5, 4′-Dihydroxy-7,3′, -dimethoxyflavone	*M. paniculata*	C_17_H_14_O_6_	314.3		Ethyl acetate	Leaves	Zhang et al. (2010)
22	7,4′-Hydroxy-5,3′-dimethoxyflavone	*M. paniculata*	C_17_H_14_O_6_	314.3		Ethanol	Leaves	Li et al. (2018)
23	5-Hydroxy-6,7,8,3′,4′,5′-hexamethoxyflavone; gardenin A	*M. paniculata*	C_21_H_22_O_9_	418.4		Methanol	Leaves	Zhang et al. (2012a)
24	5-Hydroxy-6,7,3′,4′,5′-pentamethoxyflavone	*M. paniculata*	C_20_H_20_O_8_	388.4		Methanol	Leaves	Zhang et al. (2013b)
25	5-Hydroxy-6,7,8,3′,4′-pentamethoxyflavone	*M. paniculata*	C_20_H_20_O_8_	388.4	358832	Ethanol	Leaves, stems	Wu et al. (2015)
		*M. exotica*				Ethanol	Leaves, twigs	Peng et al. (2010)
26	5-Hydroxy-7,8,3′,4′-tetramethoxyflavone	*M. paniculata*	C_19_H_18_O_7_	358.3	9950661	Ethanol	Leaves, twigs	Liang et al. (2020c)
27	5-Hydroxy-6,7,3′,4′-tetramethoxyflavone	*M. paniculata*	C_19_H_18_O_7_	358.3		Methanol	Leaves	Zhang et al. (2012b)
		*M. exotica*				Ethanol	Leaves, twigs	Peng et al. (2010)
28	5-Hydroxy-7,3′,4′-trimethoxyflavone	*M. paniculata*	C_18_H_16_O_6_	328.3		Methanol	Leaves	Zhang et al. (2011)
29	3′-Hydroxy-5,6,7,4′,5′-pentamethoxyflavone	*M. paniculata*	C_20_H_20_O_8_	388.4		Ethanol	Leaves, twigs	Liang et al. (2020c)
30	3′-Hydroxy-5,7,4′,5′-tetramethoxyflavone	*M. paniculata*	C_19_H_18_O_7_	358.3	72703223	Ethanol	Leaves	Li et al. (2018)
31	3′-Hydroxy-5,7,4′-trimethoxyflavone	*M. paniculata*	C_18_H_16_O_6_	328.3	13964545	Ethanol	Leaves	Li et al. (2018)
32	7-Hydroxy-5,3′,4′-trimethoxyflavone	*M. paniculata*	C_18_H_16_O_6_	328.3		Ethanol	Leaves	Li et al. (2018)
33	4′-Hydroxy-5,6,7,3′,5′-pentamethoxyflavone	*M. paniculata*	C_20_H_20_O_8_	388.4	44258535	Ethanol	Leaves, twigs	Liang et al. (2020c)
34	4′-Hydroxy-5,7,3′-trimethoxyflavone	*M. paniculata*	C_18_H_16_O_6_	328.3	13964546	Ethanol	Leaves	Li et al. (2018)
35	5,7,3′,4′-Tetrahydroxyflavone; luteolin	*M. paniculata*	C_15_H_10_O_6_	286.2	5280445	Hydroalcoholic	Leaves	Menezes et al. (2017)
36	5,3′-Dihydroxy-6,7,4′-trimethoxyflavone-8-*O*-*β*-glucopyranoside	*M. paniculata*	C_24_H_26_O_13_	522.5		Ethanol	Leaves, shoots	Zhang et al. (2012a)
		*M. exotica*				Methanol	Leaves	Zhang et al. (2014)
37	5,3′-Dihydroxy-6,4′-dimethoxyflavone-7-*O*-*β*-glucopyranoside	*M. exotica*	C_23_H_24_O_12_	492.4		Methanol	Leaves	Zhang et al. (2014)
38	5-Hydroxy-6,3′,4′-trimethoxyflavone-7-*O*-*β*-glucopyranoside	*M. paniculata*	C_24_H_26_O_12_	506.5		Ethanol	Leaves	Li et al. (2018)
39	5,4′-Dihydroxy-3′-methoxyflavone-7-*O*-*β*-glucopyranoside	*M. paniculata*	C_22_H_22_O_11_	462.4		Ethanol	Leaves	Li et al. (2018)
40	5,4′-Dihydroxy-6,3′-dimethoxyflavone-7-*O*-*β*-D-glucopyranoside	*M. paniculata*	C_23_H_24_O_12_	492.4		Methanol	Leaves	Zhang et al. (2013b)
41	5-Hydroxy-6,7,3′,4′,-tetramethoxyflavone-8-*O*-*β*-D-glucopyranoside	*M. paniculata*	C_25_H_28_O_13_	536.4		Methanol	Leaves	Zhang et al. (2013b)
*Flavonols*								
42	3,5,6,7,8,3′,4′,5′-Octamethoxyflavone; exoticin	*M. paniculata*	C_23_H_26_O_10_	462.4	389000	Ethyl acetate	Leaves and twigs	Li, (2022)
43	3,5,6,7,3′,4′,5′-Heptamethoxyflavone	*M. paniculata*	C_22_H_24_O_9_	432.4	389001	Methanol	Leaves	Sangkaew et al. (2020)
		*M. paniculata*				Dichloromethane	Pulp of fresh ripe fruits	Santos et al. (2009)
		*M. exotica*				Methanol	Stems, branches, twigs, leaves	Liu et al. (2015a)
44	3,5,7,8,3′,4′,5′-Heptamethoxyflavone	*M. paniculata*	C_22_H_24_O_9_	432.4	5318050	Methanol	Leaves	Sangkaew et al. (2020)
		*M. paniculata*				Methanol	Peel and pulp of ripe fruits	Santos et al. (2009)
		*M. exotica*				Acetone	Branches	Negi et al. (2015)
45	3,5,6,8,3′,4′,5′-Heptamethoxyflavone	*M. exotica*	C_22_H_24_O_9_	432.4		Petrol	Leaves	Braik et al. (1983b)
46	3,5,6,7,8,3′,4′-Heptamethoxyflavone	*M. paniculata*	C_22_H_24_O_9_	432.4	150893	Methanol	Leaves, twigs	Liang et al. (2021)
		*M. exotica*				Methanol	Leaves, twigs	Liang et al. (2021)
47	3,5,7,3′,4′,5′-Hexamethoxyflavone	*M. paniculata*	C_21_H_22_O_8_	402.4	634113	Methanol	Leaves	Sangkaew et al. (2020)
		*M. paniculata*				Chloroform	Flowers	Kinoshita and Firman (1997)
48	3,5,7,8,3′,4′-Hexamethoxyflavone	*M. paniculata*	C_21_H_22_O_8_	402.4	146093	Dichloromethane	Peel and pulp of ripe fruits	Ferracin et al. (1998)
49	5-Hydroxy-3,7,8,3′,4′,5′-hexamethoxyflavone	*M. paniculata*	C_21_H_22_O_9_	418.4		—	Leaves	Alam et al. (2021)
		*M. paniculata*				Dichloromethane	Peel of fresh ripe fruits	Santos et al. (2009)
50	5-Hydroxy-3,7,8,3′,4′-pentamethoxyflavone	*M. paniculata*	C_20_H_20_O_8_	388.4	10200272	Dichloromethane	Peel and pulp of ripe fruits	Ferracin et al. (1998)
51	8-Hydroxy-3,5,7,3′,4′,5′-hexamethoxyflavone	*M. paniculata*	C_21_H_22_O_9_	418.4		—	Leaves	Alam et al. (2021)
		*M. paniculata*				Dichloromethane	Peel of fresh ripe fruits	Santos et al. (2009)
52	Quercetin	*M. paniculata*	C_15_H_10_O_7_	302.2	5280343	Hydroalcoholic	Leaves	Menezes et al. (2017)
		*M. exotica*				Ethanol	Leaves	Kaur et al. (2016)
53	Kaempferol	*M. paniculata*	C_15_H_10_O_6_	286.2	5280863	Hydroalcoholic	Leaves	Menezes et al. (2017)
		*M. exotica*				Ethanol	Leaves	Kaur et al. (2016)
54	Kaempferide	*M. exotica*	C_16_H_12_O_6_	300.3	5281666	_	_	Li et al. (2022)
55	Quercetin-3-*O*-rhamnoside; quercitrin	*M. paniculata*	C_21_H_20_O_11_	448.4	5280459	Hydroalcoholic	Leaves	Menezes et al. (2017)
56	Quercetin-3-*O*-rutinoside (rutin)	*M. paniculata*	C_27_H_30_O_16_	610.5	5280805	Hydroalcoholic	Leaves	Menezes et al. (2017)
		*M. exotica*				Ethanol	Leaves	Kaur et al. (2016)
*Flavanones*								
57	5,6,7,3′,4′,5′-Hexamethoxyflavanone	*M. paniculata*	C_21_H_24_O_8_	404.4	42608106	Ethanol	Leaves, twigs	Liang et al. (2020b)
58	6,7,8,3′,4′,5′-Hexamethoxyflavanone	*M. paniculata*	C_21_H_24_O_8_	404.4		Ethanol	Leaves, twigs	Liang et al. (2020b)
59	5,6,7,3′,4′-Pentamethoxyflavanone	*M. paniculata*	C_20_H_22_O_7_	374.4		Methanol	Leaves	Zhang et al. (2013b)
		*M. exotica*				Methanol	Leaves	Zhang et al. (2014)
60	5,7,3′,4′,5′-Pentamethoxyflavanone	*M. paniculata*	C_20_H_22_O_7_	374.4	4692111	Chloroform	Leaves	Kinoshita and Firman (1997)
		*M. paniculata*				Dichloromethane	Peel and pulp of ripe fruits	Ferracin et al. (1998)
		*M. exotica*				Methanol	Leaves	Zhang et al. (2014)
61	5,7,8,3′,4′-Pentamethoxyflavanone	*M. paniculata*	C_20_H_22_O_7_	374.4		Ethanol	Leaves	Shan et al. (2010)
62	6,7,8,3′,4′-Pentamethoxyflavanone	*M. paniculata*	C_20_H_22_O_7_	374.4		Ethanol	Leaves	Yao et al. (2013)
63	5,7,3′,4′-Tetramethoxyflavanone	*M. paniculata*	C_19_H_20_O_6_	344.4	91212489	Ethanol	Leaves, twigs	Liang et al. (2020b)
64	3-Hydroxy-5,7,3′,4′,5′-pentamethoxyflavanone	*M. paniculata*	C_20_H_22_O_8_	390.4		Chloroform	Leaves	Kinoshita and Firman (1997)
65	3-Hydroxy-5,7,3′,4′-tetramethoxyflavanone	*M. paniculata*	C_19_H_20_O_7_	360.4		Ethanol	Leaves, twigs	Liang et al. (2020b)
66	4′-Hydroxy-5,7-dimethoxyflavanone	*M. paniculata*	C_17_H_16_O_5_	300.3	5271551	Ethanol	Leaves, twigs	Liang et al. (2020b)
67	5,6,7,3′,4′,5′- Hexamethoxyflavanone-8-*O*-[rhamnopyranosyl-(1→4)-rhamnopyranoside	*M. paniculata*	C_33_H_44_O_17_	712.7		Ethanol	Twig	Shi et al. (2017)
*Flavan-3-ols*								
68	(−)-Epicatechin; epicatechin	*M. paniculata*	C_15_H_14_O_6_	290.3	72276	Hydroalcoholic	Leaves	Menezes et al. (2017)
		*M. exotica*				Ethanol	Leaves	Kaur et al. (2016)
69	Catechin	*M. paniculata*	C_15_H_14_O_6_	290.3	9064	Hydroalcoholic	Leaves	Menezes et al. (2017)
		*M. exotica*				Ethanol	Leaves	Kaur et al. (2016)
*Chalcones*								
70	2′-Hydroxy-3,4,4′,6′-tetramethoxychalcone	*M. paniculata*	C_19_H_20_O_6_	344.4	5373259	Ethanol	Leaves, twigs	Liang et al. (2020b)
71	2′-Hydroxy-3,4,5,4′,6′-pentamethoxychalcone	*M. paniculata*	C_20_H_22_O_7_	374.4	5374858	Chloroform	Leaves	Kinoshita and Firman (1997)
72	2′-Hydroxy-3,4,3′,4′,6′-pentamethoxychalcone	*M. paniculata*	C_20_H_22_O_7_	374.7		Ethanol	Leaves, twigs	Liang et al. (2020b)
73	2′-Hydroxy-3,4,5,3′,4′,6′-hexamethoxychalcone	*M. paniculata*	C_21_H_24_O_8_	404.4	129823511	Ethanol	Leaves	Yao et al. (2013)
74	2′,4-Dihydroxy-3,5,4′,6′-tetramethoxychalcone	*M. paniculata*	C_19_H_20_O_7_	360.4		Ethanol	Leaves, twigs	Liang et al. (2020b)
75	6′-Hydroxy-3,4,5,2′,3′,4′-hexamethoxychalcone	*M. paniculata*	C_21_H_24_O_8_	404.4		Methanol	Leaves	Zhang et al. (2011)
76	6′-Hydroxy-3,4,5,2′,4′,5′-hexamethoxychalcone	*M. paniculata*	C_21_H_24_O_8_	404.4		Methanol	Leaves	Zhang et al. (2013b)
		*M. exotica*				Methanol	Leaves	Zhang et al. (2014)
77	6′-Hydroxy-3,4,5,2′,5′-pentamethoxychalcone	*M. paniculata*	C_20_H_22_O_7_	374.4		Methanol	Leaves	Zhang et al. (2013b)
		*M. exotica*				Methanol	Leaves	Zhang et al. (2014)
78	6′-Hydroxy-3,4,5,2′,4′-pentamethoxychalcone	*M. paniculata*	C_20_H_22_O_7_	374.4		Methanol	Leaves	Zhang et al. (2011)
*Isoflavones*								
79	Genistein	*M. paniculata*	C_15_H_10_O_5_	270.2	5280961	Methanol	Leaves	Lapčík et al. (2004)
80	Formononetin	*M. paniculata*	C_16_H_12_O_4_	268.3	5280378	Methanol	Leaves	Lapčík et al. (2004)
81	Prunetin	*M. paniculata*	C_16_H_12_O_5_	284.3	5281804	Methanol	Leaves	Lapčík et al. (2004)
82	Biochanin A	*M. paniculata*	C_16_H_12_O_5_	284.3	5280373	Methanol	Leaves	Lapčík et al. (2004)
83	Daidzein	*M. paniculata*	C_15_H_10_O_4_	254.2	5281708	Methanol	Leaves	Lapčík et al. (2004)
84	Daidzin	*M. paniculata*	C_21_H_20_O_9_	416.4	107971	Methanol	Leaves	Lapčík et al. (2004)
85	Glycitin	*M. paniculata*	C_22_H_22_O_10_	446.4	187808	Methanol	Leaves	Lapčík et al. (2004)
86	Genistin	*M. paniculata*	C_21_H_20_O_10_	432.4	5281377	Methanol	Leaves	Lapčík et al. (2004)
87	Daidzin-6″-*O*-malonate	*M. paniculata*	C_24_H_22_O_12_	502.4	9913968	Methanol	Leaves	Lapčík et al. (2004)
88	Glycitin-6″-*O*-malonate	*M. paniculata*	C_25_H_24_O_13_	532.4	23724657	Methanol	Leaves	Lapčík et al. (2004)
89	Daidzin-6″-*O*-acetate	*M. paniculata*	C_23_H_22_O_10_	458.4	156155	Methanol	Leaves	Lapčík et al. (2004)
90	Glycitin-6″-*O*-acetate	*M. paniculata*	C_24_H_24_O_11_	488.4	10228095	Methanol	Leaves	Lapčík et al. (2004)
91	Genistin-6″-*O*-malonate	*M. paniculata*	C_24_H_22_O_13_	518.4	15934091	Methanol	Leaves	Lapčík et al. (2004)
92	Genistin-6″-*O*-acetate	*M. paniculata*	C_23_H_22_O_11_	474.4	22288010	Methanol	Leaves	Lapčík et al. (2004)
93	Sissotrin	*M. paniculata*	C_22_H_22_O_10_	446.4	5280781	Methanol	Leaves	Lapčík et al. (2004)

Note: —Refer to “Not mention”, the same below.

**TABLE 3:** Coumarins isolated from Murrayae Folium et Cacumen.

No.	Name	Species	Formula	Weight	PubChem CID	Extract	Parts of the plant	References
*Simple coumarins*								
94	Coumarin	*M. exotica*	C_9_H_6_O_2_	146.1	323	_	_	Li et al. (2022)
95	Umbelliferone	*M. paniculata*	C_9_H_6_O_3_	162.1	5281426	Ethyl acetate	Aerial parts	Saeed et al. (2011)
		*M. exotica*				Dichloromethane	Roots	Liu et al. (2018)
		*M. exotica*				Acetone	Leaves	Xu et al. (2016)
96	7-Coumarincarboxaldehyde	*M. exotica*	C_10_H_6_O_3_	174.2		Dichloromethane	Roots	Liu et al. (2018)
97	7-Methoxycoumarin	*M. exotica*	C_10_H_8_O_3_	176.2	10748	Acetone	Leaves	Xu et al. (2016)
98	5,7-Dihydroxycoumarin	*M. exotica*	C_9_H_6_O_4_	178.1	5324654	_	_	Li et al. (2022)
99	5,7-Dimethoxycoumarin	*M. exotica*	C_11_H_10_O_4_	206.2	2775	_	_	Li et al. (2022)
100	Scopoletin	*M. paniculata*	C_10_H_8_O_4_	192.2	5280460	Ethyl acetate	Aerial part	Saeed et al. (2011)
		*M. paniculata*				Chloroform	Root bark	Imai et al. (1989)
		*M. exotica*				Acetone	Branches	Negi et al. (2015)
		*M. exotica*				Ethanol	Leaves	Wu et al. (2010)
101	Isoscopoletin	*M. exotica*	C_10_H_8_O_4_	192.2	69894	Dichloromethane	Roots	Liu et al. (2018)
		*M. exotica*				Acetone	Branches	Negi et al. (2015)
102	7-Hydroxy-8-methoxycoumarin	*M. paniculata*	C_10_H_8_O_4_	192.2		Ethyl acetate	Leaves, twigs	Li, (2022)
103	7-Methoxy-8-formylcoumarin	*M. exotica*	C_11_H_8_O_4_	204.2	11275724	Acetone	Leaves	Ito and Furukawa (1987b)
		*M. exotica*				Acetone	Branches	Negi et al. (2015)
104	Trihydroxy coumarin	*M. exotica*	C_9_H_6_O_5_	194.2		Dichloromethane	Leaves	He et al. (2017)
105	Hassanon	*M. paniculata*	C_14_H_14_O_4_	246.3		Chloroform	Roots	Wang et al. (2019a)
106	7-Methoxy-8-(2′-methyl-2′-formylpropyl)-coumarin	*M. exotica*	C_15_H_16_O_4_	260.3	5319433	Dichloromethane	Leaves	He et al. (2017)
107	Seselinal	*M. paniculata*	C_16_H_18_O_5_	290.3		Chloroform	Roots	Wang et al. (2019a)
108	7-Methoxy-8-(5-(prop-1-en-2-yloxy) enta-1,3-dien-1-yl)-coumarin	*M. exotica*	C_18_H_18_O_4_	298.3		Ethanol	Root	Jiang et al. (2015)
109	*cis*-Osthenon	*M. paniculata*	C_14_H_12_O_4_	244.3		Acetone	Leaves	Ito and Furukawa (1987a)
		*M. exotica*				Dichloromethane	Roots	Liu et al. (2018)
110	Toddalenone	*M. paniculata*	C_15_H_14_O_5_	274.3	101893838	Chloroform	Leaves	Aziz et al. (2010)
		*M. exotica*				Dichloromethane	Roots	Liu et al. (2018)
*Prenylated coumarins*								
111	Murraol	*M. exotica*	C_15_H_16_O_4_	260.3	15593213	Acetone	Leaves	Ito and Furukawa (1987b)
112	3′-*O*-Methylmurraol	*M. exotica*	C_16_H_18_O_4_	274.3	102337145	Ethyl acetate	Leaves, twigs	Li, (2022)
113	Casegravol	*M. exotica*	C_15_H_16_O_5_	276.3	6440521	Acetone	Leaves	Ito and Furukawa (1987b)
114	Murraexotin A	*M. exotica*	C_21_H_26_O_4_	342.4		Ethyl acetate	Leaves, twigs	Li, (2022)
115	Casegravol isovalerate	*M. paniculata*	C_20_H_24_O_6_	360.4	14429495	Chloroform	Roots	Wang et al. (2019a)
		*M. exotica*				Methanol	Leaves, twigs	Liang et al. (2021)
116	*cis*-Dehydroosthol	*M. exotica*	C_15_H_14_O_3_	242.3	13917397	Acetone	Leaves	Ito and Furukawa (1987b)
117	*cis*-Dehydrocoumarrayin	*M. paniculata*	C_16_H_16_O_4_	272.3		Chloroform	Leaves	Kinoshita and Firman (1996a)
118	*trans*-Dehydroosthol	*M. paniculata*	C_15_H_14_O_3_	242.3		Chloroform	Roots	Wang et al. (2019a)
		*M. exotica*				Dichloromethane	Roots	Liu et al. (2018)
		*M. exotica*				Acetone	Leaves	Ito and Furukawa (1987b)
119	*trans*-Gleinadiene	*M. paniculata*	C_16_H_16_O_4_	272.3		Chloroform	Leaves	Aziz et al. (2010)
		*M. exotica*				Dichloromethane	Roots	Liu et al. (2018)
120	(1′*S*, 3′*S*)-Murratin A	*M. exotica*	C_15_H_14_O_4_	258.3		Ethanol	Leaves, twigs	Liang et al. (2020b)
121	(1′*R*, 3′*R*)-Murratin A	*M. exotica*	C_15_H_14_O_4_	258.3		Ethanol	Leaves, twigs	Liang et al. (2020b)
122	(1′*S*, 3′*R*)-Murratin B	*M. exotica*	C_15_H_14_O_4_	258.3		Ethanol	Leaves, twigs	Liang et al. (2020b)
123	(1′*R*, 3′*S*)-Murratin B	*M. exotica*	C_15_H_14_O_4_	258.3		Ethanol	Leaves, twigs	Liang et al. (2020b)
124	Osthol	*M. paniculata*	C_15_H_16_O_3_	244.3	10228	Chloroform	Roots	Wang et al. (2019a)
		*M. paniculata*				Ethyl acetate	Leaves, twigs	Li, (2022)
		*M. exotica*				Dichloromethane	Roots	Liu et al. (2018)
		*M. exotica*				Acetone	Leaves	Ito and Furukawa (1987b)
125	7-Methoxy-8-(3′-formylbut-2′-enyl) coumarin	*M. paniculata*	C_15_H_14_O_4_	258.3		Chloroform	Roots	Wang et al. (2019a)
126	Sibiricol	*M. paniculata*	C_15_H_16_O_4_	260.3	13917413	Ethyl acetate	Leaves, twigs	Li, (2022)
		*M. exotica*				Dichloromethane	Roots	Liu et al. (2018)
		*M. exotica*				Acetone	Leaves	Ito and Furukawa (1987b)
127	Panial	*M. paniculata*	C_15_H_14_O_5_	274.3		Acetone	Leaves	Ito and Furukawa (1987a)
128	Coumurrayin	*M. paniculata*	C_16_H_18_O_4_	274.3	176911	Chloroform	Roots	Wang et al. (2019a)
		*M. paniculata*				Ethyl acetate	Leaves, twigs	Li, (2022)
		*M. exotica*				Dichloromethane	Roots	Liu et al. (2018)
		*M. exotica*				Ethyl acetate	Leaves, twigs	Li, (2022)
129	Sibirinol	*M. exotica*	C_16_H_18_O_5_	290.3		Dichloromethane	Roots	Liu et al. (2018)
130	6-Hydroxycoumurrayin	*M. paniculata*	C_16_H_18_O_5_	290.3	133561628	Ethanol	Twigs, leaves	Li et al. (2016)
131	Muralatin C	*M. exotica*	C_17_H_20_O_5_	304.3		Ethanol	Leaves, twigs	Liang et al. (2020b)
132	Phebalosin	*M. paniculata*	C_15_H_14_O_4_	258.3	188300	Methanol	Leaves	Jiwajinda et al. (2000)
		*M. paniculata*				Acetone	Roots	Ito et al. (1990)
		*M. exotica*				Ethanol	Leaves	Yan et al. (2018)
		*M. exotica*				Acetone	Branches	Negi et al. (2015)
133	Murrayone	*M. paniculata*	C_15_H_14_O_4_	258.3	5319964	Chloroform	Roots	Wang et al. (2019a)
		*M. exotica*				Dichloromethane	Leaves	He et al. (2017)
134	Micropubescin	*M. paniculata*	C_15_H_14_O_4_	258.3	14185882	Ethyl acetate	Leaves	Rodanant et al. (2015)
135	Murpanitin D	*M. paniculata*	C_20_H_23_O_6_	359.4		Ethanol	Leaves, stems	Liang et al. (2020a)
136	Murralongin	*M. paniculata*	C_15_H_14_O_4_	258.3	179620	Chloroform	Roots	Wang et al. (2019a)
		*M. paniculata*				Ethyl acetate	Leaves, twigs	Li (2022)
		*M. exotica*				Acetone	Leaves	Ito and Furukawa (1987b)
		*M. exotica*				Acetone	Branches	Negi et al. (2015)
137	Murralonginal	*M. exotica*	C_15_H_14_O_4_	258.3		Ethyl acetate	Leaves, twigs	Xia-Hou et al. (2022)
138	Murraexotin C	*M. exotica*	C_20_H_24_O_5_	344.4		Ethyl acetate	Leaves, twigs	Li, (2022)
139	Auraptenol	*M. exotica*	C_15_H_16_O_4_	260.3	13343541	Dichloromethane Chloroform	Roots	Liu et al. (2018)
		*M. exotica*					Leaves	Barik et al. (1983b)
140	(*S*)-Auraptenol	*M. exotica*	C_15_H_16_O_4_	260.3	13343540	Acetone	Leaves	Xu et al. (2016)
141	Murralonginol	*M. paniculata*	C_15_H_16_O_4_	260.3		Chloroform	Roots	Wang et al. (2019a)
142	Murratin M	*M. exotica*	C_17_H_18_O_5_	302.3		Ethanol	Leaves, twigs	Liang et al. (2020b)
143	Murralonginol isovalerate	*M. paniculata*	C_20_H_24_O_6_	360.4		Chloroform	Roots	Wang et al. (2019a)
		*M. exotica*				Ethyl acetate	Leaves, twigs	Li, (2022)
144	Isomurralonginol	*M. paniculata*	C_15_H_16_O_4_	260.3		Chloroform	Roots	Wang et al. (2019a)
		*M. exotica*				Ethyl acetate	Leaves, twigs	Xia-Hou et al. (2022)
145	Isomurralonginoic acid	*M. exotica*	C_15_H_14_O_5_	274.3		Acetone	Branches	Negi et al. (2015)
146	Isomurralonginol acetate	*M. paniculata*	C_17_H_18_O_5_	302.3	13917402	Ethyl acetate	Leaves, twigs	Li, (2022)
		*M. exotica*				Acetone	Leaves	Ito and Furukawa (1987b)
147	Isomurralonginol isovalerate	*M. paniculata*	C_20_H_24_O_6_	360.4	45359775	Chloroform	Roots	Wang et al. (2019a)
		*M. exotica*				Acetone	Branches	Negi et al. (2015)
148	Meranzin	*M. exotica*	C_15_H_16_O_4_	260.3	1803558	Ethanol	Leaves	Yan et al. (2018)
149	Sibiricin	*M. paniculata*	C_16_H_18_O_5_	290.3	12315526	Chloroform	Roots	Wang et al. (2019a)
150	Panitin E	*M. paniculata*	C_20_H_24_O_6_	360.4		Chloroform	Roots	Wang et al. (2019a)
151	Muralatin P	*M. exotica*	C_20_H_24_O_6_	360.4		Ethyl acetate	Leaves, twigs	Xia-Hou et al. (2022)
152	Isomeranzin	*M. paniculata*	C_15_H_16_O_4_	260.3	473252	Chloroform	Roots	Wang et al. (2019a)
		*M. exotica*				Acetone	Leaves	Xu et al. (2016)
		*M. exotica*				Acetone	Branches	Negi et al. (2015)
153	Murranganon	*M. paniculata*	C_15_H_16_O_5_	276.3	5319956	Methanol	Leaves	Choudhary et al. (2002)
		*M. exotica*				Acetone	Branches	Negi et al. (2015)
154	Isosibiricin	*M. paniculata*	C_16_H_18_O_5_	290.3	5316871	Chloroform	Leaves	Kinoshita and Firman (1996a)
		*M. exotica*				Chloroform	Root barks	He et al. (2017)
		*M. exotica*				—	—	Wang et al. (2019b)
155	7-Methoxy-8-(1′-acetoxy-2′-oxo-3′-methylbutyl)coumarin	*M. exotica*	C_17_H_20_O_5_	304.4		Acetone	Leaves	Ito and Furukawa (1987b)
156	Hainanmurpanin	*M. paniculata*	C_17_H_18_O_6_	318.3	5317952	Ethyl acetate	Aerial parts	Saied et al. (2008)
		*M. exotica*				Ethanol	Leaves	Yan et al. (2018)
		*M. exotica*				Acetone	Branches	Negi et al. (2005)
157	Murpanitin C	*M. paniculata*	C_19_H_23_O_6_	347.3		Ethanol	Leaves, stems	Liang et al. (2020a)
158	Epimurpaniculol senecioate	*M. exotica*	C_20_H_22_O_6_	358.4		Ethanol	Leaves, twigs	Liang et al. (2020b)
159	Murranganonsenecioate	*M. paniculata*	C_20_H_22_O_6_	358.4		Ethyl acetate	Leaves	Rodanant et al. (2015)
		*M. exotica*				Acetone	Branches	Negi et al. (2015)
160	Paniculatin	*M. paniculata*	C_20_H_24_O_6_	360.4	5320400	Ethanol	Leaves, stems	Liang et al. (2020a)
		*M. exotica*				Acetone	Branches	Negi et al. (2015)
161	Murratin F	*M. exotica*	C_21_H_26_O_10_	438.4		Ethanol	Leaves, twigs	Liang et al. (2020b)
162	Microminutin	*M. paniculata*	C_15_H_12_O_5_	272.3	5319827	Chloroform	Leaves, stems	Liang et al. (2020a)
163	Muralatin I	*M. paniculata*	C_15_H_14_O_5_	274.3		Ethyl acetate	Leaves, twigs	Li, (2022)
164	8-(2′-Oxo-3′-methyl) butoxy-7-methoxycoumarin	*M. paniculata*	C_15_H_16_O_5_	276.3		Methanol	Aerial parts	Rahman et al. (1997)
165	Peroxyauraptenol	*M. exotica*	C_15_H_16_O_5_	276.3	13917394	Acetone	Leaves	Ito and Furukawa (1987b)
166	Murrangatin	*M. paniculata*	C_15_H_16_O_5_	276.3		Ethanol	Leaves, stems	Liang et al. (2020a)
		*M. paniculata*				Chloroform	Root bark	Imai et al. (1989)
		*M.exotica*				Dichloromethane	Roots	Liu et al. (2018)
		*M.exotica*				Acetone	Leaves	Xu et al. (2016)
167	Minumicrolin	*M. paniculata*	C_15_H_16_O_5_	276.3	389002	Ethanol	Leaves, stems	Liang et al. (2020a)
		*M.exotica*				Dichloromethane	Roots	Liu et al. (2018)
		*M.exotica*				Acetone	Leaves	Xu et al. (2016)
168	Murpanidin	*M. exotica*	C_15_H_16_O_5_	276.3	6426907	Methanol	Leaves, twigs	Liang et al. (2021)
169	2′-*O*-Ethylmurrangatin	*M. paniculata*	C_17_H_20_O_5_	304.3		Ethanol	Leaves, stems	Liang et al. (2020a)
		*M. exotica*				Dichloromethane	Roots	Liu et al. (2018)
		*M. exotica*				Ethyl acetate	Leaves, twigs	Xia-Hou et al. (2022)
170	Muralatin K	*M. paniculata*	C_17_H_20_O_5_	304.3		Ethanol	Leaves, stems	Liang et al. (2020a)
		*M. exotica*				Ethyl acetate	Leaves, twigs	Xia-Hou et al. (2022)
171	(+)-Murraxocin	*M. exotica*	C_17_H_20_O_5_	304.3	188750	Dichloromethane	Roots	Zhou et al. (2020)
172	(−)-Murraxocin	*M. paniculata*	C_17_H_20_O_5_	304.3		Ethanol	Leaves, stems	Liang et al. (2020a)
		*M. exotica*				Dichloromethane	Roots	Zhou et al. (2020)
		*M. exotica*				Acetone	Leaves	Xu et al. (2016)
173	Panitin F	*M. paniculata*	C_20_H_24_O_6_	360.4		Chloroform	Roots	Wang et al. (2019a)
174	Fisovalerate	*M. paniculata*	C_20_H_24_O_6_	360.4		Ethanol	Leaves, stems Roots	Liang et al. (2020a)
		*M. exotica*				Dichloromethane		Liu et al. (2018)
175	Murrangatin 2′- isovalerate	*M. paniculata*	C_20_H_24_O_6_	360.4		Ethanol	Leaves, stems	Liang et al. (2020a)
		*M. exotica*				Dichloromethane	Roots	Liu et al. (2018)
		*M. exotica*				Acetone	Branches	Negi et al. (2015)
176	Murracarpin	*M. paniculata*	C_16_H_18_O_5_	290.3	5319464	Methanol	Leaves	Cuong et al. (2014)
		*M. exotica*				Dichloromethane	Roots	Liu et al. (2018)
		*M. exotica*				Ethanol	Leaves	Wu et al. (2010)
177	Chloculol	*M. paniculata*	C_15_H_15_ClO_4_	294.7	183084	Acetone	Roots	Ito et al. (1990)
178	7 -Methoxy-8-(2′-isovaleryloxy-3-butenyl-3-methyl)coumarin	*M. paniculata*	C_20_H_24_O_5_	344.4		Hexane	Stem barks	Srivastava et al. (1996)
179	Murrangatin 1′-isovalerate	*M. exotica*	C_20_H_24_O_6_	360.4		Acetone	Branches	Negi et al. (2015)
180	Murrangatin diacetate	*M. exotica*	C_19_H_20_O_7_	360.4	389004	Acetone	Leaves	Xu et al. (2016)
181	Murrangatin 2′-formate	*M. exotica*	C_16_H_16_O_6_	304.3		Acetone	Branches	Negi et al. (2015)
182	Murrangatin 2′-acetate	*M. paniculata*	C_17_H_18_O_6_	318.3		Ethyl acetate	Leaves	Rodanant et al. (2015)
		*M. exotica*				Acetone	Branches	Negi et al. (2015)
183	Murrangatin 1′-acetate	*M. exotica*	C_17_H_18_O_6_	318.3		Acetone	Branches	Negi et al. (2015)
184	Paniculacin	*M. paniculata*	C_26_H_36_O_6_	444.6		Ethyl acetate	Aerial part	Saeed et al. (2011)
185	Murrangatin palmitate	*M. paniculata*	C_31_H_46_O_6_	514.7		Chloroform	Root barks	Imai et al. (1989)
		*M. exotica*				Acetone	Branches	Negi et al. (2015)
186	Meranzin hydrate	*M. paniculata*	C_15_H_18_O_5_	278.3	5070783	Methanol	Leaves, twigs	Liang et al. (2021)
		*M. exotica*				Ethyl acetate	Leaves, twigs	Xia-Hou et al. (2022)
187	Yuehgesin B	*M. exotica*	C_16_H_20_O_5_	292.3		Ethyl acetate	Leaves, twigs	Li, (2022)
188	(2′S, 3′S) -Murratin G	*M. exotica*	C_15_H_18_O_6_	294.3		Ethanol	Leaves, twigs	Liang et al. (2020b)
189	Yuehgesin C	*M. exotica*	C_17_H_22_O_5_	306.4	5319451	Acetone	Leaves	Xu et al. (2016)
190	Meranzin hydrate acetate	*M. exotica*	C_17_H_22_O_5_	306.4		Petroleum ether	Leaves	Barik et al. (1983a)
191	2′-Acetoxy-3′-dihydroxyl-osthol	*M. exotica*	C_17_H_20_O_6_	320.3		Ethyl acetate	Leaves, twigs	Xia-Hou et al. (2022)
192	Murratin J	*M. exotica*	C_18_H_22_O_6_	334.3		Ethanol	Leaves, twigs	Liang et al. (2020b)
193	7-Methoxy-8-(2′-hydroxy-3′bromo)-coumarin	*M. exotica*	C_15_H_17_BrO_4_	341.2		Acetone	Leaves	Xu et al. (2016)
194	7-Methoxy-8-(2′,3′-dibromo)-coumarin	*M. exotica*	C_15_H_16_Br_2_O_3_	404.4		Acetone	Leaves	Xu et al. (2016)
195	Paniculonol isovalerate	*M. paniculata*	C_20_H_24_O_6_	360.4		Methanol	Leaves, twigs	Liang et al. (2021)
		*M. exotica*				Ethyl acetate	Leaves, twigs	Xia-Hou et al. (2022)
196	Muralatin M	*M. paniculata*	C_20_H_26_O_6_	362.4		Ethyl acetate	Leaves, twigs	Li, (2022)
197	Murrayatin	*M. paniculata*	C_20_H_26_O_6_	362.4	621354	Methanol	Aerial parts	Rahman et al. (1997)
		*M. exotica*				Acetone	Branches	Negi et al. (2015)
198	Exotimarin G	*M. exotica*	C_20_H_26_O_7_	378.2		Dichloromethane	Roots	Liu et al. (2018)
		*M. exotica*				Ethyl acetate	Leaves, twigs	Xia-Hou et al. (2022)
199	Meranzin hydrate-2′-palmitate	*M. exotica*	C_32_H_50_O_7_	546.7	101245396	Acetone	Branches	Negi et al. (2015)
200	Panitin G	*M. paniculata*	C_16_H_17_O_6_	289.3		Chloroform	Roots	Wang et al. (2019a)
201	Omphamurin	*M. paniculata*	C_16_H_18_O_5_	290.3	11778953	Chloroform	Roots	Wang et al. (2019a)
202	Omphamurin isovalerate	*M. paniculata*	C_21_H_26_O_6_	374.4	10643130	Methanol	Leaves, twigs	Liang et al. (2021)
203	Murratin H	*M. exotica*	C_15_H_18_O_6_	294.3		Ethanol	Leaves, twigs	Liang et al. (2020b)
204	Murratin I	*M. exotica*	C_15_H_18_O_6_	294.3		Ethanol	Leaves, twigs	Liang et al. (2020b)
205	Murratin K	*M. exotica*	C_20_H_26_O_7_	378.2		Ethanol	Leaves, twigs	Liang et al. (2020b)
206	Murratin L	*M. exotica*	C_20_H_26_O_7_	378.2		Ethanol	Leaves, twigs	Liang et al. (2020b)
207	Chloticol	*M. exotica*	C_15_H_17_ClO_4_	296.8		Acetone	Branches	Negi et al. (2015)
208	Auraptene	*M. paniculata*	C_19_H_22_O_3_	298.4	1550607	Petroleum ether	Leaves	Aziz et al. (2010)
209	7-Geranyloxy-6-methoxycoumarin	*M. paniculata*	C_20_H_24_O_4_	328.4	5319406	Chloroform	Roots	Wang et al. (2019a)
		*M. exotica*				Ethanol	Leaves, twigs	Liang et al. (2020b)
210	(11*S*, 12*R*)-Murpanitin A	*M. paniculata*	C_16_H_14_O_6_	302.3		Ethanol	Leaves, stems	Liang et al. (2020a)
211	(11*R*, 12*S*)-Murpanitin A	*M. paniculata*	C_16_H_14_O_6_	302.3		Ethanol	Leaves, stems	Liang et al. (2020a)
212	Murraexotin B	*M. exotica*	C_17_H_20_O_5_	304.3		Ethyl acetate	Leaves, twigs	Li, (2022)
213	Mexoticin	*M. paniculata*	C_16_H_20_O_6_	308.3	176970	Chloroform	Roots	Wang et al. (2019a)
		*M. exotica*				Acetone	Branches	Negi et al. (2015)
214	Isomexoticin	*M. paniculata*	C_16_H_20_O_6_	308.3	4465807	Ethanol	Leaves	Yang and Su (1983)
		*M.exotica*				Dichloromethane	Roots	Liu et al. (2018)
215	5,7-Dimethoxy-8-(2-hydroxyl-3-ethoxy-3-methylbutyl)coumarin	*M. paniculata*	C_18_H_24_O_6_	336.4		Chloroform	Roots	Wang et al. (2019a)
216	Exotimarin I	*M. paniculata*	C_18_H_24_O_6_	336.4		Chloroform	Roots	Wang et al. (2019a)
		*M. exotica*				Dichloromethane	Roots	Liu et al. (2018)
217	5-Methoxymurrayatin	*M. paniculata*	C_21_H_28_O_7_	392.4	10644213	Ethanol	Leaves, stems	Liang et al. (2020a)
218	Minumicrolin acetonide	*M. paniculata*	C_18_H_20_O_5_	316.3		Chloroform	Leaves, stems	Liang et al. (2020a)
		*M. exotica*				Acetone	Branches	Negi et al. (2015)
219	Pranferin	*M. exotica*	C_18_H_22_O_5_	318.3	101967153	Ethyl acetate	Leaves, twigs	Xia-Hou et al. (2022)
220	Omphalocarpin	*M. paniculata*	C_17_H_22_O_6_	322.4	101988840	Methanol	Leaves	Sangkaew et al. (2020)
		*M. exotica*				Dichloromethane	Roots	Liu et al. (2018)
221	6-(2′,3′-Dihydroxy-3-methylbutyl)-8-prenylumbelliferone	*M. paniculata*	C_19_H_24_O_5_	332.4		Chloroform	Roots	Wang et al. (2019a)
222	Isomurralonginol senecioate	*M. paniculata*	C_20_H_22_O_5_	342.4		Ethyl acetate	Leaves, twigs	Li, (2022)
		*M. exotica*				Acetone	Branches	Negi et al. (2015)
223	2-(7-Methoxy-2-oxochromen-8-yl)-3-methylbut-2-enyl] 3-methylbut-2-enoate	*M. exotica*	C_20_H_22_O_5_	342.4	45783081	Ethanol	Leaves, twigs	Liang et al. (2020b)
224	Murpanitin B	*M. paniculata*	C_19_H_21_O_6_	345.3		Ethanol	Leaves, stems	Liang et al. (2020a)
225	Kimcuongin	*M. paniculata*	C_20_H_20_O_6_	356.4	102141971	Methanol	Leaves	Cuong et al. (2014)
		*M. exotica*				Methanol	Leaves, twigs	Liang et al. (2021)
226	Exotimarin H	*M. paniculata*	C_20_H_22_O_6_	358.4		Ethanol	Leaves, stems	Liang et al. (2020a);
		*M. exotica*				Ethanol	Roots	Liu et al. (2018)
227	Panitin C	*M. paniculata*	C_20_H_22_O_6_	358.4		Chloroform	Roots;	Wang et al. (2019a)
		*M. paniculata*				Ethanol	Leaves, stems	Liang et al. (2020a);
		*M. exotica*				Ethanol	Leaves, twigs	Liang et al. (2020b)
228	Panitin D	*M. paniculata*	C_21_H_24_O_7_	388.2		Chloroform	Roots	Wang et al. (2019a)
229	Isomurranganon senecioate	*M. exotica*	C_20_H_22_O_6_	358.4		Acetone	Leaves	Ito and Furukawa (1987b)
230	Muralatin O	*M. paniculata*	C_20_H_24_O_6_	360.4		Chloroform	Roots	Wang et al. (2019a)
		*M. exotica*				Ethyl acetate	Leaves, twigs	Xia-Hou et al. (2022)
231	Panitin B	*M. paniculata*	C_21_H_26_O_7_	390.2		Chloroform	Roots	Wang et al. (2019a)
232	Isomurralonginol nicotinate	*M. paniculata*	C_21_H_19_NO_5_	375.4		Ethyl acetate	Leaves, twigs	Li, (2022)
		*M. exotica*				Dichloromethane	Roots	Liu et al. (2018)
		*M. exotica*				Ethyl acetate	Leaves, twigs	Xia-Hou et al. (2022)
233	8-(Butenyl-3′-methyl)-7 –*O*-*β*-D-galactopyranoside	*M. paniculata*	C_19_H_22_O_9_	378.2		Hexane	Stem barks	Srivastava et al. (1996)
234	Marmesin-4′-*O*-*α*-L-arabinopyranoside	*M. paniculata*	C_20_H_22_O_9_	406.4		Hexane	Stem barks	Srivastava et al. (1996)
235	Toddacoumaquinone	*M. paniculata*	C_23_H_18_O_7_	406.4	10046907	Chloroform	Roots	Wang et al. (2019a)
236	Exotines A	*M. exotica*	C_28_H_27_NO_3_	425.2		Ethanol	Roots	Liu et al. (2015b)
237	Exotines B	*M. exotica*	C_29_H_29_NO_4_	455.6		Ethanol	Roots	Liu et al. (2015b)
*Pyranocoumarins*								
238	Xanthyletin	*M. exotica*	C_14_H_12_O_3_	228.2	65188	Dichloromethane	Roots	Liu et al. (2018)
239	Braylin	*M. exotica*	C_15_H_14_O_4_	258.3	618370	Ethyl acetate	Leaves, twigs	Li, (2022)
240	Nordentatin	*M. exotica*	C_19_H_20_O_4_	312.4	5320206	Dichloromethane	Roots	Liu et al. (2018)
Furanocoumarin*s*								
241	Xanthotoxol	*M. exotica*	C_11_H_6_O_4_	202.2	65090	—	—	Li et al. (2022)
242	Bergaptol	*M. exotica*	C_11_H_6_O_4_	202.2	5280371	—	—	Li et al. (2022)
*Coumarin dimers*								
243	(10′*R*,11′*R*,12*R*)-Exotimarin F	*M. exotica*	C_31_H_36_O_11_	584.6		Dichloromethane	Roots	Liu et al. (2018)
244	5-Demethoxy-10′-ethoxyexotimarin F	*M. exotica*	C_32_H_38_O_10_	582.3		Ethyl acetate	Leaves, twigs	Xia-Hou et al. (2022)
245	(11*R*,12*R*) -Exotimarin A	*M. exotica*	C_24_H_20_O_7_	420.4		Dichloromethane	Roots	Liu et al. (2018)
246	Cladimarin A	*M. paniculata*	C_26_H_22_O_8_	462.4	12108777	Ethyl acetate	Leaves, twigs	Li, (2022)
247	Cladimarin B	*M. paniculata*	C_26_H_22_O_9_	478.4	101271041	Chloroform	Roots	Wang et al. (2019a)
		*M. exotica*				Dichloromethane	Roots	Liu et al. (2018)
		*M. exotica*				Acetone	Branches	Negi et al. (2015)
248	Exotimarin D	*M. exotica*	C_26_H_26_O_9_	482.4		Dichloromethane	Roots	Liu et al. (2018)
249	Exotimarin B	*M. exotica*	C_30_H_28_O_8_	516.6		Dichloromethane	Roots	Liu et al. (2018)
250	(+)-Exotimarin C	*M. exotica*	C_30_H_30_O_8_	518.6		Dichloromethane	Roots	Liu et al. (2018)
251	(−)-Exotimarin C	*M. exotica*	C_30_H_30_O_8_	518.6		Dichloromethane	Roots	Liu et al. (2018)
252	Murratin E	*M. exotica*	C_30_H_32_O_8_	520.6		Ethanol	Leaves, twigs	Liang et al. (2020b)
253	Toddasin	*M. paniculata*	C_31_H_30_O_8_	530.6	101999460	Chloroform	Leaves	Kinoshita and Firman (1996a)
254	Mexolide	*M. exotica*	C_32_H_32_O_8_	544.6	54598332	Benzene	Stem barks	Chakraborty et al. (1980)
255	Murradimerin A	*M. exotica*	C_30_H_32_O_8_	520.6	12146410	Ethyl acetate	Leaves, twigs	Xia-Hou et al. (2022)
256	Bismurrangatin	*M. exotica*	C_30_H_30_O_9_	534.6		Acetone	Branches	Negi, et al. (2005)
257	Murrmeranzin	*M. paniculata*	C_30_H_32_O_9_	536.6		Ethyl acetate	Aerial parts	Saied et al. (2008)
258	Exotimarin E	*M. exotica*	C_30_H_32_O_10_	552.6		Dichloromethane	Roots	Liu et al. (2018)
259	Toddalosin	*M. exotica*	C_32_H_34_O_9_	562.6	15071281	Dichloromethane	Roots	Liu et al. (2018)
260	Murramarin A	*M. exotica*	C_32_H_34_O_10_	578.6		Acetone	Branches	Negi et al. (2005)
261	Panitin A	*M. paniculata*	C_32_H_36_O_10_	580.6		Chloroform	Roots	Wang et al. (2019a)
*Benzocoumarins*								
262	8-Methylbenzo [*h*]coumarin	*M. exotica*	C_14_H_10_O_2_	210.2		Ethanol	Leaves, twigs	Liang et al. (2020b)
263	8-(3-Methylbutanoyloxy)methylbenzo [*h*] coumarin	*M. exotica*	C_19_H_18_O_4_	310.3		Ethanol	Leaves, twigs	Liang et al. (2020b)

**TABLE 4:** Alkaloids isolated from Murrayae Folium et Cacumen.

No.	Name	Species	Formula	Weight	PubChem CID	Extract	Parts of the plant	References
*Quinoline alkaloids*								
264	Edulitine	*M. paniculata*	C_11_H_11_NO_3_	205.2	826073	Chloroform	Root bark	Imai et al. (1989)
265	2,4-Quinolinediol	*M. exotica*	C_9_H_7_NO_2_	162.2	5280371	—	—	Li et al. (2022)
266	4-Hydroxy-1-methyl-2-quinolone	*M. exotica*	C_10_H_9_NO_2_	175.2	54686436	—	—	Li et al. (2022)
*Indazole alkaloids*								
267	Indazole	*M. paniculata*	C_7_H_6_N_2_	118.1	9221	Ethyl acetate	Leaves, twigs	Li, (2022)
*Indole alkaloids*								
268	Murrayaculatine	*M. paniculata*	C_10_H_9_NO_4_	207.2	101416188	Butanol	Flowers	Wu et al. (1994)
269	1H-indole	*M. exotica*	C_8_H_7_N	117.2	798	—	—	Li et al. (2022)
270	Indol-3-carbaldehyde	*M. paniculata*	C_9_H_7_NO	145.2	10256	Chloroform	Roots	Wang et al. (2017)
271	Tanakine	*M. paniculata*	C_13_H_17_NO_2_	219.3	57357311	Chloroform	Roots	Wang et al. (2017)
272	3-Prenylindole	*M. paniculata*	C_13_H_15_N	185.3	10867041	Acetone	Roots	Ito et al. (1990)
273	Paniculidine A	*M. paniculata*	C_14_H_17_NO_2_	231.3	14166401	Chloroform	Roots	Wang et al. (2017)
274	Paniculidine B	*M. paniculata*	C_14_H_19_NO_2_	233.3	14070748	Chloroform	Roots	Wang et al. (2017)
275	Paniculidine C	*M. paniculata*	C_13_H_17_NO	203.3	11264158	Chloroform	Roots	Wang et al. (2017)
276	Paniculidine D	*M. paniculata*	C_14_H_19_NO_2_	233.3		Chloroform	Roots	Wang et al. (2017)
277	Paniculol	*M. paniculata*	C_13_H_17_NO	203.3		Acetone	Roots	Ito et al. (1990)
278	Paniculidine E	*M. paniculata*	C_26_H_30_N_2_O_2_	402.5		Chloroform	Roots	Wang et al. (2017)
279	Paniculidine F	*M. paniculata*	C_29_H_33_NO_6_	491.3		Chloroform	Roots	Wang et al. (2017)
280	Yuehchukene	*M. paniculata*	C_26_H_26_N_2_	366.5	126009	Chloroform	Roots	Wang et al. (2017)
*Carbazole alkaloids*								
281	3-Methyl-9H-carbazole	*M. exotica*	C_13_H_11_N	181.2	20746	—	—	Li et al. (2022)
282	3-Methyl-9H-carbazol-2-ol	*M. exotica*	C_13_H_11_NO	197.2	3459141	—	—	Li et al. (2022)
283	1-Methoxy-3-methyl-9H-carbazole	*M. exotica*	C_14_H_13_NO	211.3	375150	—	—	Li et al. (2022)
284	Mahanimbine	*M. exotica*	C_23_H_25_NO	331.4	167963	Petroleum ether	Stem barks	Bhattacharyyaet al. (1978)
285	Girinimbine	*M. exotica*	C_18_H_17_NO	263.3	96943	Petroleum ether	Stem barks	Roy and Bhattacharyya (1981)
286	Koenimbine	*M. exotica*	C_19_H_19_NO_2_	293.4	97487	Petroleum ether	Stem barks	Roy and Bhattacharyya (1981)
287	Euchrestifoline	*M. paniculata*	C_18_H_17_NO_2_	279.3	25172103	Methanol	Leaves	Rehman et al. (2013)
288	Murrayazoline	*M. exotica*	C_23_H_25_NO	331.4	21770913	Petroleum ether	Stem barks	Bhattacharyyaet al. (1978)
289	Murrayazolinol	*M. exotica*	C_23_H_25_NO_2_	347.4	180314	—	—	Ahmad, (1994)
290	Exozoline	*M. exotica*	C_23_H_27_NO	333.5	101324894	Ethanol	Leaves	Ganguly and Sarkar (1978)
*Organic amine alkaloid*								
291	Methyl N-methyl anthranylate	*M. paniculata*	C_9_H_11_NO_2_	165.2	6826	Chloroform	Root barks	Imai et al. (1989)
*Other alkaloid*								
292	Acetazolamide	*M. paniculata*	C_4_H_6_N_4_O_3_S_2_	222.3	1986	Methanol	Leaves	Sangkaew et al. (2020)

**TABLE 5:** Sterols, phenylpropenols, organic acids and spirocyclopentenones isolated from Murrayae Folium et Cacumen.

No.	Name	Species	Formula	Weight	PubChem CID	Extract	Parts of the plant	References
*Sterols*								
293	*β*-Sitosterol	*M. paniculata*	C_29_H_50_O	414.7	222284	Ethyl acetate	Aerial parts	Saeed et al. (2011)
294	(23*S*)-23-Ethyl-24-methyl-cycloart-24(24^1^)-en-3*β*-ol	*M. exotica*	C_33_H_56_O	468.8		Cyclohexane	Leaves	Desoky, (1995)
295	3*β*-Methoxy-(23*S*)-23-ethyl-24-methyl-cycloart-24(24^1^)-en-3*β*-ol	*M. exotica*	C_34_H_58_O	482.8		Cyclohexane	Leaves	Desoky, (1995)
296	(23*S*)-23-Ethyl-24-methyl-cycloart-24(24^1^)-3*β*-yl acetate	*M. exotica*	C_35_H_60_O	496.8		Cyclohexane	Leaves	Desoky, (1995)
297	(23ζ)-23-Isopropyl-24-methyl-cycloart-25-en-3*β*-ol	*M. exotica*	C_34_H_58_O	482.8		Cyclohexane	Leaves	Desoky, (1995)
298	(23ζ)-23-Isopropyl-24-methyl-cycloart-25-en-3*β*-yl acetate	*M. exotica*	C_36_H_62_O	510.9		Cyclohexane	Leaves	Desoky, (1995)
*Phenylpropenols*								
299	2-(*p*-Hydroxyphenyl)ethyl *p*-coumarate	*M. exotica*	C_17_H_16_O_4_	284.3		Dichloromethane	Roots	Liu et al. (2018)
300	Exotiacetal A	*M. exotica*	C_28_H_30_O_6_	462.5		Dichloromethane	Roots	Liu et al. (2018)
301	Exotiacetal B	*M. exotica*	C_28_H_30_O_6_	462.5		Dichloromethane	Roots	Liu et al. (2018)
302	Exotiacetal C	*M. exotica*	C_28_H_30_O_5_	446.5		Dichloromethane	Roots	Liu et al. (2018)
*Organic acids*								
303	3-Hydroxybenzoic acid	*M. exotica*	C_7_H_6_O_3_	138.1	7420	—	—	Li et al. (2022)
304	4-Hydroxybenzoic acid	*M. paniculata*	C_7_H_6_O_3_	138.1	135	Ethyl acetate fraction	Aerial parts	Saeed et al. (2011)
305	Gallic acid	*M. paniculata*	C_7_H_6_O_5_	170.1	370	Ethanol	Leaves	Kaur et al. (2016)
		*M. exotica*				Hydroalcoholic	Leaves	
306	Coumaric acid	*M. exotica*	C_9_H_8_O_3_	164.2	637542	Ethanol	Leaves	Menezes et al. (2017)
307	*trans*-Cinnamic acid	*M. paniculata*	C_9_H_8_O_2_	148.2	444539	Ethylacetate	Aerial parts	Kaur et al. (2016)
308	Caffeic acid	*M. paniculata*	C_9_H_8_O_4_	180.2	689043	Ethanol	Leaves	Saeed et al. (2011)
		*M. exotica*				Hydroalcoholic	Leaves	
309	tert-Butylhydroquinone	*M. exotica*	C_10_H_14_O_2_	166.2	16043	Ethanol	Leaves	Kaur et al. (2016)
310	Methyl 2,5-dihydroxycinnamate	*M. paniculata*	C_10_H_10_O_4_	194.2	5353609	Methanol	Aerial parts	Menezes et al. (2017)
311	Methyl 2-methoxy-5-hydroxycinnamate	*M. paniculata*	C_11_H_12_O_4_	208.1		Methanol	Aerial parts	Kaur et al. (2016)
312	Murraxonin	*M. exotica*	C_13_H_13_O_5_	249.2		Chloroform	Leaves	Rahman et al. (1997)
313	Ellagic acid	*M. paniculata*	C_14_H_6_O_8_	302.2	5281855	Ethanol	Leaves	Rahman et al. (1997)
		*M. exotica*				Hydroalcoholic	Leaves	
314	Chlorogenic acid	*M. paniculata*	C_16_H_18_O_9_	354.3	1794427	Ethanol	Leaves	Braik and Kundu (1987)
		*M. exotica*				Hydroalcoholic	Leaves	
*Spirocyclopentenones*								
315	(1*S*, 5*S*)-Murrayaspiroketone	*M. paniculata*	C_20_H_21_O_10_	421.1		Ethanol	Leaves, stems	Menezes et al. (2017)
316	(1*R*, 5*R*)-Murrayaspiroketone	*M. paniculata*	C_20_H_21_O_10_	421.1		Ethanol	Leaves, stems	Kaur et al. (2016)

### 4.1 Flavonoids

Ninety-three flavonoids (**1–93**, Table 2) have been identified in MFC. Of these, 72 compounds belong to the polymethoxylated flavonoid family, 90 were isolated from *M. paniculata*, and 24 were isolated from *M. exotica*, confirming that flavonoids were isolated mainly from *M. paniculata* (Lu et al., 2021a). Twenty-one compounds were isolated from *M. paniculata* and *M. exotica* (Negi et al., 2015; Sangkaew et al., 2020). Depending on their structure, flavonoids can be divided into six types: flavones, flavonols, flavanones, flavan-3-ols, chalcones, and isoflavones; of these, flavones are the primary structures of flavonoids in MFC (Figure 3).

### 4.2 Coumarins

One hundred and seventy coumarins (**94–263**, Table 3) have been identified in MFC. Of these, 89 compounds were isolated from *M. paniculata*, 130 compounds were isolated from *M. exotica*, and 49 were isolated from both *M. paniculata* and *M. exotica* (Liu et al., 2018; Wang X. et al., 2019; Wang Y. et al., 2019). Depending on their structure, coumarins can be classified into six types: simple coumarins, prenylated coumarins, pyranocomarins, furanocoumarins, dimeric coumarins, and benzocoumarins (Figure 4).

### 4.3 Alkaloids

Twenty-nine alkaloids (**264**–**292**, Table 4) have been identified in MFC. Of these, 17 compounds were isolated from *M. paniculata,* and 12 compounds were isolated from *M. exotica*. No alkaloids have been isolated in both *M. paniculata* and *M. exotica.* Depending on their structure, alkaloids can be divided into six types: quinoline, indazole, indole, carbazole, organic amine, and other alkaloids (Figure 5).

### 4.4 Sterols

Six sterols (**293**–**298**, Table 5) have been isolated from MFC (Desoky, 1995; Saeed et al., 2011) (Figure 5).

### 4.5 Phenylpropenols

Four phenylpropenols (**299**–**302**, Table 5) have been isolated from the roots of *M. exotica* (Liu et al., 2018), compounds **300** and **301** of which are isomers (Figure 5).

### 4.6 Organic acids

Twelve organic acids (**303**–**314**, Table 5) have been isolated from MFC (Rahman et al., 1997; Saeed et al., 2011; Kaur et al., 2016; Menezes et al., 2017) (Figure 5).

### 4.7 Spirocyclopentenones

Two spirocyclopentenones (**315** and **316**, Table 5) have been isolated from *M. paniculata* (Liang et al., 2020a), including (1*S*, 5*S*)-murrayaspiroketone (**315**) and (1*R*, 5*R*)-murrayaspiroketone (**316**). Compounds **315** and **316** are enantiomers (Figure 5).

### 4.8 Volatile oils

Four-hundred and four volatile oils (**317**–**711**, Supplementary Table S) have been isolated from MFC (Pino et al., 2006; Raina et al., 2006; Krishnamoorthy et al., 2015; Silva et al., 2020; Saikia et al., 2021). The major volatile organic compounds dominated by benzenoids, sesquiterpenes, diterpenoids, triterpenoids, coumarins, and phenylethanoids in hydrodistillation and pentane, *n*-hexane, and dichloromethane extracts using GC-MS and gas chromatography-flame ionization detection (GC-FID). Of these, 287 compounds were isolated from *M. paniculata,* 244 compounds from *M. exotica*, and 127 from both *M. paniculata* and *M. exotica.* Sesquiterpenes are the predominant constituents of the essential oils from MFC. The main compounds are *β*-caryophyllene, spathulenol, *α*-zingiberene, *α*-copaene, germacrene D, and methyl palmitate (Lv et al., 2013; Dosoky et al., 2016; Silva et al., 2019).

## 5 Pharmacological activities

**FIGURE 6:**
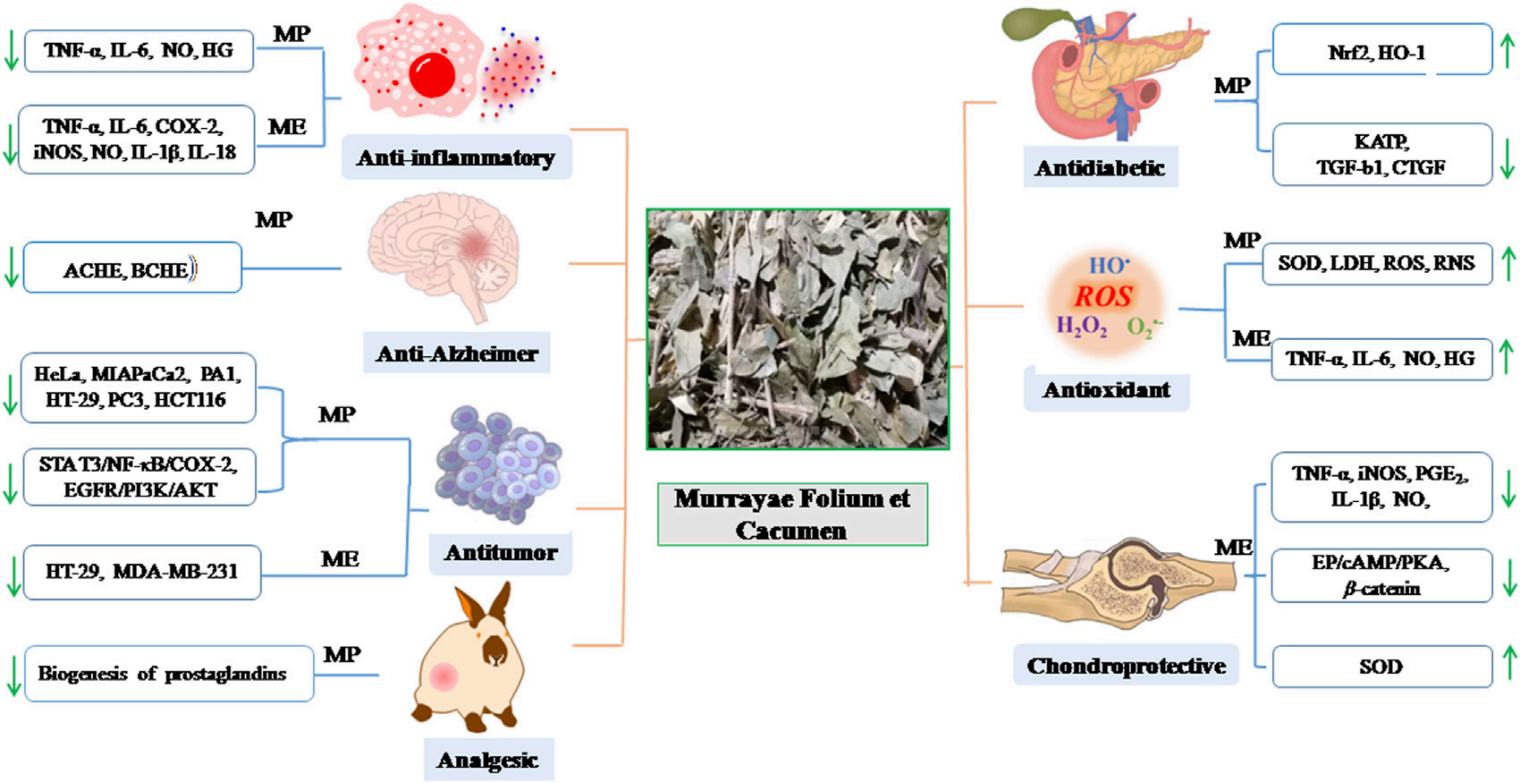
The pharmacological activity mechanism of Murrayae Folium et Cacumen (MP: *Murraya. Paniculata*; ME: *M. exotica*).

Modern pharmacological research has indicated that MFC has anti-inflammatory, anti-bacterial, anti-microbial, anti-diabetic, anti-tumor, and anti-oxidant properties (Lu et al., 2021a). The mechanisms of the compounds, extracts, and fractions from MFC are summarized in Table 6 and discussed in subsequent sections. Figure 6 summarizes the pharmacological mechanisms of MFC.

**TABLE 6:** Pharmacological Mechanism, models of compounds and various extracts in Murrayae Folium et Cacumen.

Activities	Resource	Compounds/extracts	*In vivo*/*in vitro* and Dosage	Experimental Model (Animals/Cell lines)	Mechanisms of action	References
Anti-inflammatory	*M. exotica*	Isosibiricin (**154**)	*In vitro*	BV-2 cell, Balb/c mice;	Inhibiting TNF-*α* and IL-6 production, reducing COX-2 and iNOS expression	Wang et al. (2019b)
			Dose range: 0–50 µm	Positive control:		
				Sultopride		
	*M. exotica*	Isomeranzin (**152**)	*In vivo*:	Female C57BL/6 mice; murine Raw 264.7 cells	Inhibiting the expression of IL-1β and IL-6 mRNA and NO release via the inhibition of NF-*κ*B and ERK signals	Xu et al. (2016)
			10 and 30 mg/kg			
			*In vitro*:			
			3, 10, and 30 µm			
	*M. paniculata*	5,7,3′,4′,5′-pentamethoxyflavone (**8**), 5,7,3′,4′-tetramethoxyflavone (**9**), 5-hydroxy-6,7,8,3′,4′-pentamethoxyflavone (**25**) (70% Ethanol extract of stems and leaves)	*In vitro*:	RAW 264.7, GES-1 cells	Reducing NO production and IL-6 production	Wu et al. (2015)
			0.01, 0.1, 1, 10, and 100 µm			
			IC_50_: 53.40 µm (**8**)			
			120.98 µm (**9**)			
			10.73 µm (**25**)			
	*M. paniculata*	Total flavonoids (Ethanol extract of leaves)	*In vitro*: 25, 50, 75, 100, and 200 μg/mL;	H9c2 cells;	Inhibiting HG-induced expression of TNF-*α* and IL-6	Zou et al. (2021)
			*In vivo*: 35 and 70 mg/kg	Male Wistar rats		
	*M. paniculata*	3′,4′,5′,7-tetramethoxyflavone (**10**), micropubescin (**134**), murranganonsenecionate (**159**), Murrangatin (**166**), murrangatin 2′-acetate (**182**) (Ethyl acetate extract of leaves)	*In vitro*: 20 and 50 μg/mL	HGFs and U937 cells	Suppressing the IL-1β production through LPS-stimulated macrophage	Rodanant et al. (2015)
				Positive controls:		
				0.5 μg/mL Dexamethasone and 0.5 μg/mL LPS		
				Negative control:		
				1% DMSO		
	*M. exotica*	*cis*-osthenon (**109**), *trans*-dehydroosthol (**118**), sibirinol (**129**), Exotiacetal A (**300**) (95% Aqueous EtOH extract of roots)	*In vitro*	BV-2 microglial cells	Inhibiting against LPS-induced NO production	Liu et al. (2018)
			IC_50_: 16.9 ± 1.0 µm (**109**)			
			11.8 ± 0.9 µm (**118**)			
			15.5 ± 0.9 µm (**129**)			
			8.6 ± 0.9 µm (**300**)			
	*M. paniculata*	*trans*-dehydroosthol (**118**), exotimarin I (**216)**, Panitin D (**228**) (95% Aqueous EtOH extract of roots)	*In vitro*	BV-2 microglial cells	Inhibiting against LPS-induced NO production	Wang et al. (2019a)
			IC_50_: 12.4 ± 0.9 µm (**118**)			
			26.9 ± 0.8 µm (**216**)			
			19.6 ± 2.3 µm (**228**)			
	*M. exotica*	70% Ethanol extract of leaves	*In vivo*:	Male mice; rats Positive control:	Inhibiting the production of iNOS, decreasing IL-1β and TNF-α and elevating the activity of SOD	Wu et al. (2010)
			50, 100, and 200 mg/kg	0.5 mg/kg Hexadecadrol		
	*M. exotica*	CH_2_Cl_2_ fraction (95% Aqueous EtOH extract of leaves and twigs)	*In vitro*	RAW 264.7	Inhibiting the release of NO by inhibiting iNOS protein	Liang et al. (2020b)
			Positive control:	Positive control:		
			IC_50_ = 15.7 ± 1.1 µm	Dexamethasone		
	*M. paniculata/M. exotica*	Ethanol extract of twigs and leaves	*In vivo*:	ICR mice, SD rats Positive controls:	Decreasing the TNF-*α* and PGE2 in plasma	Lu et al. (2021a)
			100, 300, and 600 mg/kg	5 mg/kg Dexamethasone		
				5 mg/kg Domperidone		
Antibacterial and antimicrobial	*M. paniculata*	3′,4′,5′,7-tetramethoxyflavone (**10**), micropubescin (**134**), murranganonsenecionate (**159**), Murrangatin (**166**), murrangatin 2′-acetate (**182**) (Ethyl acetate extract of leaves)	*In vitro*: 100 μg/mL	*Prophyromonas* *gingivalis*	Exhibiting antibacterial activity against *P. gingivalis*	Rodanant et al. (2015)
				Positive control:		
				2% Chlorhexidine		
				Negative control:		
				DMSO		
	*M. paniculata*	Volatile oils (Leaves by hydrodistillation)	*In vitro*: 10 µL	*Pseudomonas aeruginosa*	Exhibiting against *Pseudomonas aeruginosa* and *Mycobacterium smegmati*s	Saikia et al. (2021)
			MIC = 4 μg/mL	*Mycobacterium smegmati*s		
	*M. paniculata*	Volatile oils (Leaves by hydrodistillation)	*In vitro*	Bacterial species	Inhibiting *Klebsiella pneumoniae* and *Bacillus subtilis*	Rodríguez et al. (2012)
	*M. paniculata*	Volatile oils (Ripe and unripe fruits by hydrodistillation)	*In vitro*	Bacterial strains	Inhibiting *Mycobacterium kansasii* and *M. tuberculosis*	Silva et al. (2020)
			Dose range:	Positive controls:		
			400–3.9 μg/mL	Chlorhexidine dihydrochloride;		
			*Mycobacterium kansasii* (MIC = 250 μg/mL)	Isoniazid		
			*M. tuberculosis* (MIC = 500 μg/mL)			
Antitumor activity	*M. paniculata*	5,6,7,3′,4′,5′-Hexamethoxyflavanone-8-*O*- [rhamnopyranosyl-(1→4)-rhamnopyranoside] (**67**) (75% Ethanol extract of twigs)	*In vitro*	A549, PC9 cells	Interrupting the STAT3/NF-κB/COV-2 and EGFR signaling pathways	Shi et al. (2017)
			Dose range:			
			1–100 μg/mL			
	*M. paniculata*	Phebalosin (**132**), murralongin (**136**) (Ethyl acetate extract of roots)	*In vitro*	HCT116 cells	Down-regulating EpCAM expression	Shao et al. (2016)
			Dose range:	Positive control:		
			1–100 μg/mL	Warfarin		
	*M. paniculata*	Volatile oils (Leaves by hydrodistillation)	*In vitro*	L6, MIA-PaCa2, PA1 and Hela cells	Inhibiting HeLa, L6, MIAPaCa2 and PA1 cell lines	Saikia et al. (2021)
			HeLa cells:			
			IC_50_ = 6.28 ± 1.82 μg/mL			
			L6 cells:			
			IC_50_ = 13.62 ± 4.02 μg/mL			
			MIA-PaCa2 cells:			
			IC_50_ = 55.12 ± 0.77 μg/mL			
			PA1 cells:			
			IC_50_ = 13.14 ± 1.56 μg/mL			
	*M. paniculata*	Volatile oils (Fresh leaves by hydrodistillation)	*In vitro*	Hepa 1c1c7 cells	Inhibiting Hepa 1c1c7 cells	Selestino Neta et al. (2017)
			Dose range:			
			7.8–500 μg/mL			
			IC_50_ = 63.7 μg/mL			
	*M. exotica*	Chloroform and methylene chloride extracts of roots and leaf parts, respectively	*In vitro*:	MDA-MB-231 cells	Inhibiting IL-1β, inducing VCAM-1 expression	He et al. (2017)
			1, 10, 50, and 100 μg/mL			
	*M. exotica*	Ethyl acetate extract of roots and leaves	*In vitro*:	HT-29 cells	Inhibiting HT-29 tumor cells	Jiang et al. (2015)
			1, 10, 50, and 100 μg/mL			
	*M. paniculata*	Dichloromethane fractions (75% Refluxing ethanol extract of twigs)	*In vitro*	HT29 cell lines	Down-regulating the expression of integrin β1, α6, CD44 in HT-29 cells and E-selectin in endothelial cells	Jiang et al. (2016)
			Dose range:			
			1–200 μg/mL			
			IC_50_ = 145.8 μg/mL			
			EC_50_ = 18.43 μg/mL			
Antidiabetic activity	*M. paniculata*	5,6,7,3′,4′,5′-Hexamethyl flavone (**3**), 5,6,7,3′, 4′-pentamethoxyl flavone (**6**), 5,7,3′,4′, 5′-pentamethoxyl flavone (**8**), 5,7,3′, 4′-tetramethoxy flavone (**9**), 7-hydroxyl-5,3′, 4′-trimethyl flavone (**32**)		Rats	Reducing the blood glucose level	Jin et al. (2008)
	*M. paniculata*	Total flavonoids (95% Ethanol extract of air-dried leaves)	*In vivo*:	Male Wistar rats	Decreasing the expression of TGF-β1 and CTGF protein	Zou et al. (2014)
			35 and 70 mg/kg	Positive control:		
				10 mg/kg Captopril		
	*M. paniculata*	Total flavonoids (95% Ethanol extract of dried leaves)	*In vivo*:	H9c2 cells;	Increasing Nrf2 and HO-1 gene expression	Zou et al. (2021)
			35 and 70 mg/kg	Male Wistar rats		
			*In vitro*:	Negative control:		
			25, 50, 75, 100, and 200 μg/mL	0.5%CMC-Na		
	*M. paniculata*	50% Ethanol extract of leaves	*In vivo*:	Male Wistar rats	Reducing the blood glucose, TGs, and cholesterol levels	Menezes et al. (2017)
			100, 200, and 400 mg/kg	Positive controls:		
				5 mg/kg Glibenclamide		
				50 mg/kg Metformin		
Antioxidant activity	*M. paniculata*	Volatile oils of leaves	*In vitro*	Positive controls:	Showing strong antioxidant activity	Rodríguez et al. (2012)
				Thymol;		
				Butylated hydroxyanisole;		
				Butylated hydroxytoluene;		
				Propyl gallate		
	*M. exotica*	Ethanol extract of fresh leaves (Polyphenols and flavonoids)	*In vitro*		Exhibiting the antioxidant activity	Kaur et al. (2016)
			10–1,000 μg/mL			
	*M. exotica*	Methanol extract of leaves	*In vitro* *:*	Positive control:	Indicating the marked antioxidant activity	Khatun et al. (2014)
			1, 5, 10, 50, and 100 μg/mL IC_50_ = 1.25 μg/mL; IC_90_ = 4.4 μg/mL	Ascorbic acid		
			Positive control:			
			IC_50_ = 0.01 μg/mL; IC_90_ = 3.58 μg/mL			
Chondroprotective activity	*M. exotica*	5,7,3ʹ,4ʹ-Tetramethoxyflavone (**9**)	*In vivo*:	Rats;	Inhibiting EP/cAMP/PKA signaling pathway and β-catenin signaling pathway	Wu et al. (2014)
			25, 50 and 100 mg/kg	Joint cartilage cells		
			*In vitro*:			
			5, 10, and 20 μg/mL			
	*M. exotica*	5,7,3ʹ,4ʹ-Tetramethoxyflavone (**9**)	*In vivo*:	Rats;	Up-regulating Foxo3a expression and inhibiting miR-29a/Wnt/β-catenin signaling activity	Huang et al. (2019)
			25 and 100 mg/kg	Joint cartilage cells		
			*In vitro*:			
			5 and 20 μg/mL			
	*M. exotica*	70% Ethanol extracts of leaves	*In vivo*:	Rats	Decreasing the IL-1β and TNF-α contents through inhibiting *β*-catenin signaling pathway	Wu et al. (2013)
			50, 100, and 200 mg/kg			
	*M. exotica*	70% Ethanol extracts of leaves	*In vivo*:	Rats	Increasing the SOD activity, inhibiting the NO activity, and decreasing the IL-1β and TNF-α contents	Wu et al. (2010)
			50, 100, and 200 mg/kg			
Potential anti-Alzheimer activity	*M. paniculata*	murranganone (**153**), (**160**), 2′-*O*-ethylmurrangatin (**169**) (Methanolic extract of air-dried leaves)	*In vitro*	-	Inhibiting AChE and BChE activities	Khalid et al. (2023)
			Positive control (IC_50_):			
			Tacrine 0.021 µm (AChE)			
			0.025 µm (BChE)			
			Galanthamine 0.45 µm (AChE)			
			32.5 µm (BChE)			
			Murranganone 79.1 µm (AChE)			
			74.3 µm (BChE)			
			Paniculatin 31.6 µm (AChE)			
			>100 µm (BChE)			
	*M. paniculata*	Germacrene D, α-zingiberene, δ-elemene (Leaves by hydrodistilation)	*In vitro*	-	Inhibiting AChE and BChE activities	El-Shiekh et al. (2023)
			IC_50_:			
			5.1 ± 0.3 μg/mL (BChE)			
			13.2 ± 0.9 μg/mL (AChE)			
Analgesic activity	*M. paniculata*	Barks extracts of petroleum ether, ethyl acetate and methanol in equal proportions	*In vivo*:	Mice	Inhibiting the writhing and extending the tail flicking time	Podder et al. (2011)
			200 and 400 mg/kg	Positive control:		
				2 mg/kg Morphine		
				50 mg/kg Aminopyrine		
Other activities	*M. paniculata*	Yuehchukene (**280**) (Chloroform extract of roots)	*In vivo*:	SD rats	having the anti-implantation and estrogenic activities	Kong et al. (1985a)
			2.5 and 3 mg/kg			
	*M. paniculata*	Chloroform fraction and ethanol extracts of leaves	*In vivo*:	Laca mice	Showing significant anxiolytic and antidepressant activities	Sharma et al. (2017)
			100, 200, and 400 mg/kg	Positive control:		
				2 mg/kg Diazepam		
				10 mg/kg Imipramine		
	*M. paniculata*	Chloroform extract of leaves	*In vitro*	-	Exhibiting moderate anti-giardial activity *in vitro*	Sawangjaroen et al. (2005)
			MIC = 250 μg/mL			
			IC_50_ = 144.87 ± 19.45 μg/mL			
	*M. paniculata*	Chloroform extract of leaves	*In vitro*	Positive control:	Showing moderate anti-amoebic activity	Sawangjaroen et al. (2006)
			Dose range:	Metronidazole		
			31.25–1,000 μg/mL			
			IC_50_ = 116.5 ± 3.5 μg/mL			
			Positive control:			
			IC_50_ = 1.1 ± 0.1 μg/mL			

### 5.1 Anti-inflammatory activity

Gastric ulcers promote the release of inflammatory factors leading to acute and chronic gastric mucosal lesions (Wu et al., 2015). MFC is the main crude material of a patented Chinese drug compound “Sanjiu Weitai,” indicated for gastritis therapy (Lu et al., 2021a), indicating that MFC could exert anti-inflammatory effects.

A pharmacodynamical study demonstrated that the levels of interleukin (IL)-6, tumor necrosis factor-alpha (TNF-*α*), and prostaglandin E2 (PGE2) in the plasma of rats were significantly decreased at 300 and 600 mg/kg of *M. paniculata* and *M. exotica*, respectively, and there was no statistical difference in the inhibition of inflammatory cytokines between *M. paniculata* and *M. exotica* at the same dose, indicating a good anti-inflammatory effect (Lu et al., 2021a). Prenylated phenylpropenols and coumarin derivatives from MFC exhibit anti-inflammatory effects by inhibiting lipopolysaccharide (LPS)-induced nitric oxide (NO) production in BV-2 microglial cells (Liu et al., 2018; Wang X. et al., 2019). The total flavonoids of *M. paniculata* leaves efficiently exert anti-inflammatory effects by inhibiting high glucose-induced expression of TNF-*α* and IL-6 in H9c2 cells (Zou et al., 2021). Coumarin derivatives isolated from the extracts of *M. exotica* leaves and twigs show anti-inflammatory activity by inhibiting the release of NO via the nitric oxide synthase (iNOS) protein (Liang et al., 2020b). Wu et al. (2010) evaluated the anti-inflammatory activity models of ethanol extracts and coumarin compounds of *M. exotica* and reported that the mechanism of action may involve proinflammatory cytokines, such as IL-1β and TNF-α. Murracarpin (**176**) shows the most potential for anti-inflammatory activity.

Isosibiricin (**154**), a natural bioactive coumarin compound isolated from MFC, markedly inhibits the release of NO and production of TNF-α and IL-6 and reduces the expression of cyclooxygenase-2 (COX-2) and inducible iNOS in a concentration-dependent manner to exert anti-inflammatory effects (Kinoshita and Firman, 1996a; Wang Y. et al., 2019). Isomeranzin (7-methoxy-8-(3-methyl-2-oxobutyl) coumarin, **152**) isolated from MFC produces the greatest inhibitory effect on proinflammatory factors, such as IL-1β and IL-6 mRNA expression and NO release. This suggests that compound **152** exerts anti-inflammatory effects primarily by selectively targeting macrophages via the inhibition of nuclear factor-kappa B (NF-*κ*B) and extracellular signal-regulated kinase signals (Xu et al., 2016). The 2′-*O*-Ehylmurrangatin (**169**) isolated from *M. paniculata* leaves promotes moderate respiratory burst inhibition, which could have potential anti-inflammatory effects (Shaikh and Choudhary, 2011).

Murrangatin (**166**), murrangatin 2′-acetate (**182**), murranganonsenecionate (**159**), micropubescin (**134**), and 3′,4′,5′,7-tetramethoxyflavone (**10**) isolated from the leaves of the ethyl acetate extract of *M. paniculata* also show strong anti-inflammatory activity via LPS-stimulated macrophages to produce the inhibitory effect (Rodanant et al., 2015). Three flavonoids (**8**, **9**, **25**) isolated from the dried stems and leaves of *M. paniculata* mainly suppress LPS-activated production of NO and IL-6 with little cytotoxicity in a dose-dependent manner. Wu et al. (2015) suggested that the C-2, 3 double bond might have an important inhibitory effect on the production of NO and IL-6 by LPS-activated RAW 264.7 cells and mouse peritoneal macrophages.

### 5.2 Anti-bacterial and anti-microbial activities

To date, studies on anti-bacterial and anti-microbial activities have been focused on *M. paniculata*, not *M. exotica.*

Murrangatin (**166**), murrangatin 2′-acetate (**182**), murranganonsenecionate (**159**), micropubescin (**134**), and 3′,4′,5′,7-tetramethoxyflavone (**10**) isolated from the leaves of ethyl acetate extract of *M. paniculata* are active against *Porphyromonas gingivalis*. Compounds **166** and **182** show better anti-bacterial potency than the crude extract, indicating that coumarins are the main constituents responsible for the anti-bacterial effects (Rodanant et al., 2015). Different extracts of *M. paniculata* used against different strains of human pathogenic bacteria have exhibited a broad spectrum of anti-bacterial activities. The ethanol and hydroalcoholic extracts show mild-to-moderate activity against human pathogenic bacteria. The methanol extract of *M. paniculata* leaves shows obvious antibacterial activities against Gram-positive and Gram-negative bacteria. Anti-bacterial properties against human pathogens with high phenol and flavonoid content have been reported (Gautam et al., 2012a). One study reported that the ethanol extract of *M. paniculata* leaf showed broad-spectrum activity in inhibiting the growth of *Staphylococcus aureus* (half minimal inhibitory concentration [MIC_50_] = 406 μg/mL) (Panda et al., 2020).

Saikia et al. (2021) reported that volatile oils showed promising anti-bacterial activity against *Mycobacterium smegmatis* (MIC_50_ = 4 μg/mL) and *Pseudomonas aeruginosa* (MIC_50_ = 4 μg/mL). Twenty-nine compounds in the volatile oils of *M. paniculata* leaves were identified using GC-MS. Most of the volatile oils were sesquiterpene hydrocarbons (80%); the major compound was caryophyllene (20.93%). Silva et al. (2020) reported that volatile oils were also effective against *Mycobacterium kansasii* (MIC_50_ = 250 μg/mL) and moderately active against *Mycobacterium tuberculosis,* demonstrating anti-streptococcal and anti-mycobacterial activities (MIC_50_ = 500 μg/mL). GC-FID and GC-MS were used to analyze the volatile oils of *M. paniculata* leaves using hydrodistillation. Anti-fungal tests *in vitro* showed that essential oils had a 91.2% inhibitory effect on the growth of mycelia in *Sclerotinia sclerotiorum*. Eighteen compounds were identified using GC-MS in the volatile oils of *M. paniculata* leaves obtained using hydrodistillation. Rodríguez et al. (2012) reported that the volatile oils exerted moderate inhibitory effects against *Klebsiella pneumoniae* and *Bacillus subtilis*. GC-MS analysis of fresh leaf extract of *M. paniculata* by hydrodistillation identified 13 compounds using GC-MS analyses, including 99.3% sesquiterpenes and 0.3% monoterpenes. The major compounds were *β*-caryophyllene (**320**), *α*-zingiberene (**325**), and *α*-caryophyllene (**336**). Another study indicated that the volatile oils and *β*-caryophyllene (**320**) exhibited moderate anti-bacterial activity (MIC_50_ < 1.0 mg/mL) (Selestino Neta et al., 2017).

### 5.3 Anti-tumor (cytotoxic) activity

Polymethoxyflavones (PMFs) exhibit a wide range of biological activities, including anti-inflammatory, anti-carcinogenic, and anti-tumor activities. Owing to the hydrophobicity of the methoxyl groups relative to the hydroxyl groups, PMFs are more lipophilic and inhibit tumor cell growth compared with hydroxylated flavonoids (Pan et al., 2007). The roots of *M. paniculata* and *M. exotica* are rich in coumarins, which exhibit cytotoxic activity in tumor cell lines (Shao et al., 2016; He et al., 2017). The leaves of *M. paniculata* also exhibits cytotoxic activity (Selestino Neta et al., 2017; Saikia et al., 2021).

The 5, 6, 7, 3′, 4′, 5′-hexamethoxyflavanone-8-*O*-[rhamnopyranosyl-(1→4)-rhamnopyranoside] (**67**) isolated from 75% ethanol extract of twigs of *M. paniculata* inhibited the adhesion, migration and invasion of lung adenocarcinoma A549 cells *in vitro.* Compound **67** blocked the adhesion of A549 cells to human pulmonary microvascular endothelial cells by targeting COX-2, matrix metalloproteinase (MMP)-2, and MMP-9 and blocked the NF-*κ*B/signal transducer and activator of transcription 3 and epidermal growth factor receptor/phosphatidylinositol 3-kinase/protein kinase B signaling pathways, thereby blocking the invasion and migration of targeted cancer cells and downregulating their epithelial–mesenchymal transition phenotype. Compound **67** inhibited the adhesion of cancer cells to abnormal endothelial cells by regulating the cellular microenvironment (Shi et al., 2017).

The raw methanol/dichloromethane extracted fraction (containing flavonoids and coumarins) of *M. paniculata* had a high adhesion inhibition rate, and the inhibition effect on human endothelial cells HT29 was concentration-dependent (1–30 μg/mL). In addition, this fraction inhibited the invasion and migration of HT29 cells. Oral administration of the fraction substantially inhibited lung metastasis in immunized mice inoculated with murine melanoma cells, without obvious side effects (Jiang et al., 2016).

Phebalosin (**132**) and murralongin (**136**), isolated from 80% acidic ethanol extracts of *M. paniculata* roots, inhibited the adhesion of cancer cells to the vascular intima because they specifically targeted cell–cell adhesion at low concentrations (Shao et al., 2016). Pharmacodynamic experiments showed that coumarin extracts from *M. exotica* roots had low cytotoxicity and high resistance to HT-29 tumor cells and could substantially inhibit the migration of tumor cells (Jiang et al., 2015). The root extracts of *M. exotica* were more efficient in restraining cell migration and had a slightly lower inhibition of cell adhesion in MDA-MB-231 cells than the leaf extracts *in vitro*. Compounds isolated from the roots of *M. exotica* show obvious inhibitory effects on the adhesion and migration of tumor cells (He et al., 2017).​

The volatile oils from fresh leaves of *M. paniculata* exhibited a half maximal inhibitory concentration (IC50) value of 63.73 μg/mL for tumorous cells of hepatocytes in a study by Selestino Neta et al. (2017). Twenty-nine compounds in the essential oil of M. paniculata leaf were identified using GC-MS. The major compound was caryophyllene (20.93%), which had an obvious inhibitory effect on HeLa, MIAPaCa2, and PA1 cell lines (Saikia et al., 2021).

### 5.4 Anti-diabetic activity

The total flavonoids extracted from *M. paniculata* (TFMP) effectively alleviated kidney damage in diabetic rats. The effects of TFMP on diabetic nephropathy may involve the regulation of glucose, lipid metabolism, oxidative stress, and inflammatory cytokines (Zou et al., 2014). Zou et al. (2021) found that TFMP could decrease the occurrence of diabetic cardiomyopathy in type 2 diabetic rats, and the protective effect may upregulate the expression of the NF-E2-related factor 2 and heme oxygenase-1 genes, inhibiting oxidative stress, inflammation, and apoptosis (Zou et al., 2021). The extract of *M. paniculata* leaves decreases glucose levels in alloxan-induced diabetic rats, effectively treating diabetes-related complications, such as hypercholesterolemia and hypertriglyceridemia, and reducing the damage associated with diabetic status. The hypoglycemic effect is similar to that of glibenclamide and metformin, which are related to the inhibition of ATP-sensitive potassium channels (Menezes et al., 2017).

The flavones extracted from *M. paniculata* leaf mainly include 5, 6, 7, 3′, 4′, 5′-hexamethyl flavone (**3**), 5, 6, 7, 3′, 4′-pentamethoxyl flavone (**6**), 5, 7, 3′, 4′, 5′-pentamethoxyl flavone (**8**), 5, 7, 3′, 4′-tetramethoxy flavone (**9**), and 7-hydroxyl-5, 3′, 4′-trimethyl flavone (**32**). A patent disclosed that the flavonoids could remarkably reduce blood glucose; improve the disturbance of lipid metabolism; increase the C-peptide level and the content of insulin in the serum; improve the excretion index of β cells of insulin and insulin resistance; reduce the insulin resistance index, malondialdehyde content in the blood serum, and contents of IL-1β, IL-6, and TNF-α; and improve superoxidase dismutase (SOD) activity (Jin et al., 2008).

### 5.5 Anti-oxidant activity

Various *in vitro* studies have shown that 50% ethanol extract of *M. paniculata* leaves possessed strong anti-oxidant activity (Gautam et al., 2012b). GC-MS was used to identify 18 compounds in volatile oils of *M. paniculata* leaves obtained via hydrodistillation. These volatile oils showed strong anti-oxidant activity, and the main component is *β*-caryophyllene (**320**) (Rodríguez et al., 2012).

Research has shown that ethyl acetate fraction of *M. exotica* leaves, which is rich in polyphenols and flavonoids, exhibits the highest anti-oxidant activity of the different parts obtained using the sequential extraction method (Kaur et al., 2016). The methanol extract of *M. exotica* leaves has been reported to contain numerous flavonoids and polyphenols, and these compounds show marked anti-oxidant activity (Khatun et al., 2014).

### 5.6 Chondroprotective activity

Wu and coworkers (2010) found the 70% ethanol extract of *M. exotica* leaves significantly reduced iNOS activity and IL-1β and TNF-α contents, increaseed SOD activity, decreaseed NO production, protected cartilage and chondrocytes from destruction, and maintained the normal function of femoral condyle cartilage and the arrangement of different layers of chondrocytes. Another study demonstrated that 70% ethanol extracts of *M. exotica* decreased the contents of TNF-α and IL-1β in rat osteoarthritis synovial fluid by inhibiting the *β*-catenin signaling pathway, and reduced chondrocyte apoptosis *in vitro* (Wu et al., 2013). 5,7,3ʹ,4ʹ-Tetramethoxyflavone (**9**) has been demonstrated to improve chondrocyte apoptosis by inhibiting Wnt/*β*-catenin signaling *in vivo* and *in vitro* (Wu et al., 2014). Further studies show that compound **9** from *M. exotica* exhibits chondroprotective activity by upregulating Foxo3a expression and inhibiting miR-29a/Wnt/*β*-catenin signaling activity (Huang et al., 2019).

### 5.7 Potential anti-Alzheimer’s disease activity

Alzheimer’s disease (AD) is a neurodegenerative disease that causes progressive loss of neuronal structure and function, leading to cognitive decline and dementia (Kabir et al., 2021). Some natural products have promising anti-AD properties. Acetylcholinesterase/butyrylcholinesterase (AChE/BChE) inhibitors are desirable because they improve cognition with minimal side effects (Wojtunik-Kulesza et al., 2021). In one study, the essential oil of *M. paniculata* leaves was the most potent selective BChE inhibitor with an IC_50_ of 5.1 ± 0.3 μg/mL and showed strong inhibitory activity against AChE (IC_50_ = 13.2 ± 0.9 μg/mL). Germacrene D (**324**), α-zingiberene (**325**), and δ-elemene (**333**) had a high affinity for BChE. These volatiles, with their *in vitro* cholinesterase inhibitory potential, have demonstrated a novel and safe treatment for AD (El-Shiekh et al., 2023). Experimentally, paniculatin (**160**) is most potent against AChE (IC_50_ = 31.6 µM), whileas murranganone (**153**) is the most potent against BChE (IC_50_ = 74.3 µM); neither compound shows selectivity toward any of the two enzymes. Paniculatin (**160**) is a mixed-type inhibitor of both AChE and BChE, whereas murranganone (**153**) promotes pure noncompetitive inhibition of AChE and BChE. Compound 2′-*O*-ethylmurrangatin (**169**) has no inhibitory effect on AChE but has a very weak inhibitory effect on BChE. These three coumarins represent a new class of natural coumarins that are active against cholinesterases. Therefore, they can be considered potential candidates for AD treatment. These coumarins show non-selective, moderate-to-good *in vitro* activity against both AChE and BChE via a mixed-type inhibitory mechanism (Khalid et al., 2023).

### 5.8 Analgesic activity

The bark extract of *M. paniculata* (200 and 400 mg/kg) has an obvious inhibitory effect on the writhing bodies of mice, and the degree of the inhibitory effect increases with increasing dose. The results at both doses are comparable to those of the standard drug aminopyrine. A similar analgesic activity was observed using the radiant heat method (Podder et al., 2011).

### 5.9 Other activities

Yuehchukene (**280**) from *M. paniculata* has been shown to exhibit long-standing anti-implantation and estrogenic activity (Kong YC. et al., 1985).

*M. paniculata* is widely used to treat mental health disorders. The chloroform (200 mg/kg) and ethanol (400 mg/kg) extracts of *M. paniculata* leaves demonstrate marked anxiolytic and anti-depressant activities, respectively (Sharma et al., 2017). The chloroform extract of *M. paniculata* leaves showed a moderate level of anti-giardial and anti-amoebic activity *in vitro* in patients with AIDS in southern Thailand (Sawangjaroen et al., 2005; Sawangjaroen et al., 2006).

Cuong et al. (2014) reported that the chloroform fraction of the methanol extract of *M. paniculata* leaves had the most potent vasorelaxing effect on rat aortic rings contracted using 60 mM K^+^. Kimcuongin (**225**) and murracarpin (**176**) isolated from the chloroform fraction showed vasorelaxant activity with IC_50_ values of 37.7 µM and 139.3 µM, respectively, suggesting that *M. paniculata* has an anti-hypertensive effect (Cuong et al., 2014).

## 6 Pharmacodynamics and pharmacokinetics

### 6.1 Pharmacodynamics

One pharmacodynamic study of MFC indicated that both *M. exotica* and *M. paniculata* markedly inhibited the writhing reaction induced by acetic acid in mice and the paw swelling induced by carrageenan in rats; decreased IL-6, TNF-*α,* and PGE2 levels in the plasma of rats with swollen paws; and increased the gastric emptying rate and intestinal propulsive rate in a dose-dependent manner. *M. exotica* and *M. paniculata* did not indicate marked differences at the same dose and are therefore considered to have similar anti-inflammatory, analgesic, and gastrointestinal motion-promoting effects (Lu et al., 2021a). The authors compared the preventive effects of *M. exotica* and *M. paniculata* against alcohol-induced gastric lesions. MFC effectively attenuated ethanol-HCl-induced gastric diseases by reversing inflammatory development and preventing ethanol-HCl-induced necrosis and apoptosis (Lu et al., 2021b).

### 6.2 Pharmacokinetics

A novel, fast, and sensitive UPLC–tandem mass spectrometry method with a sample preparation procedure, low limit of quantitation, short run time, and good accuracy was used to easily detect murrayone (**133**) extracted from *M. paniculata* in rat plasma. This method has been successfully applied in pharmacokinetic studies. Zhai et al. (2020) studied Sprague-Dawley rats administered with 20, 50, or 125 mg/kg murrayone intra-gastrically or 20 mg/kg murrayone via intravenous bolus injection. The mean T_max_ values of the relevant pharmacokinetic parameters ranged from 0.75 h to 1 h for all doses, indicating rapid murrayone absorption. The mean T_1/2_ was 3.52–5.97 h for all dose groups, indicating that the rate of murrayone elimination was also rapid. The relatively high Vd/F indicated that murrayone was widely distributed in the body and combined with tissues. The absorption rate of murrayone was high, and the absolute bioavailability of murrayone was 22.72%–37.81% in rats. The exposure level of murrayone was positively correlated with the dose administered, indicating that the *in vivo* pharmacokinetic behavior of murrayone is linear (Zhai et al., 2020). ​

## 7 Toxicology

The constituents and extracts of MFC have been studied for their pharmacological activity. However, studies on the potential toxicology of these compounds are limited.

In male and female Swiss mice, acute toxicity was assessed using a single oral dose of the hydroethanolic extract of *M. paniculata* leaves (2,000 and 5,000 mg/kg bw/day). No abnormal signs of toxicity (e.g., piloerection, diarrhea, or alteration in locomotor activity) or death were observed during the 14 days of observation (Menezes et al., 2015). The aqueous extracts of *M. paniculata* leaves against *Artemia salina* were presented as total phenolic compounds. The percentage mortality of brine shrimp increased with the concentration of the aqueous extract of *M. paniculata*, which has been shown to have a significant effect on brine shrimp (Bovornvattanangkul and Jiraungkoorskul, 2016). Liaqat et al. (2018) studied Wistar rats with free access to food and water that were administered with 400 mg/kg volatile oils of *M. paniculata*. The MIC and MBC results showed that the oils of *M. paniculata* were active against Gram-positive strains. Moreover, elevation in packed cell volume and depletion in mean corpuscular volume were observed; therefore, the volatile oils of *M. paniculata* are considered safe for internal use (Liaqat et al., 2018). The half lethal concentration (LC_50_) and 90% lethal concentration (LC_90_) values of petroleum ether extract and crude methanol extract from *M. paniculata* leaves were 0.471 ± 0.72 μg/mL and 0.773 ± 0.19 μg/mL, respectively, showing high cytotoxic activity in a brine shrimp lethal test (Mita et al., 2013). Another study showed that the ethanol extract (250 and 500 mg/kg dosages) of *M. paniculata* leaves produced strong anti-nociceptive activity and was toxic to brine shrimp (half lethal dose [LD_50_] = 32 μg/mL) (Sharker et al., 2009).

Khatun et al. (2014) identified the methanol extract from *M. exotica* leaves using a brine shrimp lethality assay. After 24 h, the LC_50_ and LC_90_ values of the extract were 1.27 μg/mLand 5.09 μg/mL, respectively, indicating cytotoxic effects (*p* < 0.01). The volatile oils of *M. exotica* possessed fumigant toxicity against *Sitophilus zeamais* and *Tribolium castaneum* adults, with LC_50_ values of 8.29 and 6.84 mg/L, respectively. The essential oils also showed contact toxicity against *S. zeamais* and *T. castaneum* adults with LD_50_ values of 11.41 and 20.94 μg/adult, respectively (Li et al., 2010).

## 8 Conclusion and future perspectives

MFC contains at least 720 components, 404 of which are volatile oils. Crude extracts and their chemical compounds have been shown to exert anti-inflammatory, anti-bacterial and anti-microbial, antitumor, anti-oxidant, anti-diabetic, anti-Alzheimer, and analgesic effects. Flavonoids, coumarins, and volatile oils are the most important bioactive compounds with pharmacological activity.

The compounds of *M. paniculata* and *M. exotica* differ in that flavonoids are the main compounds in *M. paniculata*, whereas coumarins are the main compounds in *M. exotica*. Flavonoid compounds, particularly polymethoxyflavones, exhibit anti-inflammatory, anti-bacterial, anti-microbial, antitumor, and chondroprotective effects. Coumarin compounds, especially prenylated coumarins, exhibit anti-inflammatory, anti-bacterial, anti-microbial, antitumor, potential anti-Alzheimer, chondroprotective, anti-implantation, estrogenic, and anti-hypertensive properties. Studies on volatile oil compounds have primarily focused on their anti-bacterial, anti-microbial and potential anti-Alzheimer effects.

Despite the remarkable outcomes of previous studies on MFC (*M. paniculata* and *M. exotica*), some questions remain, and further research is needed to bridge the current scientific gap. First, although MFC demonstrates considerable pharmacological activity, little is known about the active parts and components, and further research and exploration are warranted. Second, we focused more on the analysis of polymethoxyflavones and coumarins and less on the quality control of MFC. A quality control assessment of MFC is needed to ensure quality. Third, there are many studies on the pharmacological activities of *M. paniculata* and *M. exotica*; however, only two comparative studies on the pharmacodynamics have shown no statistical differences between *M. paniculata* and *M. exotica.*

Meanwhile, in pharmacological studies, most of the studies are limited to *in vitro* cell studies, while *in vivo* studies are rarely involved. So far, only two studies are related to clinical indications, which limits the further clinical use of MFC. In view of these gaps and challenges, we should focus on *in vivo* pharmacological research, the relationship between efficacy and mechanism of action, and activity research on the structure-activity relationship of compounds in the future. Fourth, the antitumor activity of MFC has been described as cytotoxic, but no *in vivo* model is currently available and further *in vivo* model and clinical tumor patient studies are needed. Fifth, there are no systematic reports on the mechanisms of MFC. MFC is mainly used in the treatment of stomach pain, rheumatism, arthralgia, toothache, and tumefaction; however, the underlying mechanisms remain unclear, except for the protective effects on gastric lesions. Volatile oils are the most abundant and promote important insecticidal activity; however, their composition varies markedly depending on their origin. Therefore, there is an urgent need to establish quality standards for MFC.

At present, the phenomenon of “heterogeneous equivalence” is widespread in many multisource traditional Chinese medicines. Therefore, several problems are associated with its clinical application, such as efficacy equivalence and quality control (Lu et al., 2021b). To ensure its efficacy and safety, it is necessary to study the rationality of two source plants, *M. paniculata* and *M. exotica,* used as the same kind of MFC. Therefore, we conducted a comprehensive review of the phytochemistry, pharmacology, pharmacodynamics, pharmacokinetics, and toxicity of *M. paniculata* and *M. exotica*. This systematic review of MFC provides a material and theoretical basis for rational and effective utilization and also provides direction for in-depth research.

In conclusion, although there have been many studies on the phytochemical and pharmacological effects of MFC, there are still many aspects that require further research and control to lay a theoretical foundation for their heterogeneous use.

## Abbreviations

AChE, acetylcholinesterase; AD, Alzheimer’s disease; BChE, butyrylcholinesterase; COX-2, cyclooxygenase-2; GC-FID, gas chromatography-flameionization detection; Foxo3a, Forkhead box class O 3a; GC-MS, gas chromatography-mass spectrometry; IC_50_, half maximal inhibitory concentration; IL, interleukin; iNOS, nitric oxide synthase; LC_50_, lethal concentration 50; LPS, lipopolysaccharide; MFC, Murrayae Folium et Cacumen; MIC, minimum inhibitory concentration; MIC_50_, half minimal inhibitory concentration; MMP, matrix metalloproteinase; NF-κB, nuclear factor-kappa B; NO, nitric oxide; PGE2, and prostaglandin E2; PMFs, polymethoxyflavones; TFMP, total flavonoids extracted from *Murraya paniculata*; TNF-α, tumor necrosis factor alpha; SOD, superoxidase dismutase.
