# Supplementary material for: A comprehensive review of the botany, phytochemistry, pharmacology, and toxicology of Murrayae Folium et Cacumen
Source: Front Pharmacol. 2024 Mar 28;15:1337161. doi: 10.3389/fphar.2024.1337161 (PMC11007094; doi:10.3389/fphar.2024.1337161)
Supplement: Supplementary file 1 [file Table1.DOCX]

TABLE S Volatile oils isolated from Murrayae Folium et Cacumen

| NO. | Name | Species | Molecular Formula | Molecular Weight | Pubchem CID | Extract | Parts | References |
| --- | --- | --- | --- | --- | --- | --- | --- | --- |
| 317 | *α*-Cubebene | *M. paniculata*  *M. exotica* | [C_15_H_24_](https://pubchem.ncbi.nlm.nih.gov/#query=C21H44) | 204.4 | 442359 | Hydrodistillation  Hydrodistillation | Unripe and ripe fruits  Branches with leaves | Silva et al. (2020)  You et al. (2015) |
| 318 | *α*-Copaene | *M. paniculata*  *M. exotica* | [C_15_H_24_](https://pubchem.ncbi.nlm.nih.gov/#query=C21H44) | 204.4 | 12303902 | Hydrodistillation Hydrodistillation | Unripe and ripe fruits  Branches with leaves | Silva et al. (2020)  You et al. (2015) |
| 319 | *α*-Gurjunene | *M. paniculata*  *M. exotica* | [C_15_H_24_](https://pubchem.ncbi.nlm.nih.gov/#query=C21H44) | 204.4 | 15560276 | Hydrodistillation  Hydrodistillation | Unripe and ripe fruits  Leaves | Silva et al. (2020)  Krishnamoorthy et al. (2015) |
| 320 | *β*-Caryophyllene | *M. paniculata*  *M. exotica* | [C_15_H_24_](https://pubchem.ncbi.nlm.nih.gov/#query=C21H44) | 204.4 | 5281515 | Hydrodistillation  Hydrodistillation | Unripe and ripe fruits  Leaves | Silva et al. (2020)  Krishnamoorthy et al. (2015) |
| 321 | *γ*-Muurolene | *M. paniculata*  *M. exotica* | [C_15_H_24_](https://pubchem.ncbi.nlm.nih.gov/#query=C21H44) | 204.4 | 12313020 | Hydrodistillation  Hydrodistillation | Unripe and ripe fruits  Leaves | Silva et al. (2020)  Krishnamoorthy et al. (2015) |
| 322 | *α*-Humulene | *M. paniculata*  *M. exotica* | [C_15_H_24_](https://pubchem.ncbi.nlm.nih.gov/#query=C21H44) | 204.4 | 5281520 | Hydrodistillation  Hydrodistillation | Unripe and ripe fruits  Leaves | Silva et al. (2020)  Pino et al. (2006) |
| 323 | Aromadendrene | *M. paniculata*  *M. exotica* | [C_15_H_24_](https://pubchem.ncbi.nlm.nih.gov/#query=C21H44) | 204.4 | 91354 | Hydrodistillation  Hydrodistillation | Unripe and ripe fruits  Leaves, twigs | Silva et al. (2020)  Lv et al. (2013) |
| 324 | Germacrene D | *M. paniculata*  *M. exotica* | [C_15_H_24_](https://pubchem.ncbi.nlm.nih.gov/#query=C21H44) | 204.4 | 5317570 | Hydrodistillation  Hydrodistillation | Unripe and ripe fruits  Leaves | Silva et al. (2020)  Pino et al. (2006) |
| 325 | *α*-Zingiberene | *M. paniculata*  *M. exotica* | [C_15_H_24_](https://pubchem.ncbi.nlm.nih.gov/#query=C21H44) | 204.4 | 11127403 | Hydrodistillation  Hydrodistillation | Unripe and ripe fruits  Branches with leaves | Silva et al. (2020)  You et al. (2015) |
| 326 | Bicyclogermacrene | *M. paniculata*  *M. exotica* | [C_15_H_24_](https://pubchem.ncbi.nlm.nih.gov/#query=C21H44) | 204.4 | 13894537 | Hydrodistillation  Hydrodistillation | Unripe and ripe fruits  Leaves | Silva et al. (2020)  Pino et al. (2006) |
| 327 | *β*-Cadinene | *M. paniculata*  *M. exotica* | [C_15_H_24_](https://pubchem.ncbi.nlm.nih.gov/#query=C21H44) | 204.4 | 10657 | Hydrodistillation  Hydrodistillation | Unripe and ripe fruits  Leaves | Silva et al. (2020)  Pino et al. (2006) |
| 328 | Cadina-1.4-diene | *M. paniculata*  *M. exotica* | [C_15_H_24_](https://pubchem.ncbi.nlm.nih.gov/#query=C21H44) | 204.4 | 6427091 | Hydrodistillation  Hydrodistillation | Unripe and ripe fruits  Aerial parts | Silva et al. (2020)  Li et al. (2010) |
| 329 | Germacrene-D-4-ol | *M. paniculata*  *M. exotica* | [C_15_H_26_](https://pubchem.ncbi.nlm.nih.gov/#query=C21H44)O | 222.4 | 5352847 | Hydrodistillation  Hydrodistillation | Unripe and ripe fruits  Leaves | Silva et al. (2020)  Pino et al. (2006) |
| 330 | Caryophyllene oxide | *M. paniculata*  *M. exotica* | [C_15_H_24_](https://pubchem.ncbi.nlm.nih.gov/#query=C21H44)O | 220.4 | 1742210 | Hydrodistillation  Hydrodistillation | Unripe and ripe fruits  Aerial parts | Silva et al. (2020)  Li et al. (2010) |
| 331 | *τ*-Muurolol | *M. paniculata*  *M. exotica* | [C_15_H_26_](https://pubchem.ncbi.nlm.nih.gov/#query=C21H44)O | 222.4 | 3084331 | Hydrodistillation  Hydrodistillation | Unripe and ripe fruits  Leaves | Silva et al. (2020)  Krishnamoorthy et al. (2015) |
| 332 | *α*-Cadinol | *M. paniculata*  *M. exotica* | [C_15_H_26_](https://pubchem.ncbi.nlm.nih.gov/#query=C21H44)O | 222.4 | 10398656 | Hydrodistillation  Hydrodistillation | Unripe and ripe fruits  Leaves | Silva et al. (2020)  Pino et al. (2006) |
| 333 | *δ*-Elemene | *M. paniculata*  *M. exotica* | [C_15_H_24_](https://pubchem.ncbi.nlm.nih.gov/#query=C21H44) | 204.4 | 12309449 | Hydrodistillation  Hydrodistillation | Leaves  Fresh leaves and flowers | Saikia et al. (2021)  Raina et al. (2006) |
| 334 | *β*-Elemene | *M. paniculata*  *M. exotica* | [C_15_H_24_](https://pubchem.ncbi.nlm.nih.gov/#query=C21H44) | 204.4 | 6918391 | Hydrodistillation  Hydrodistillation | Leaves  Leaves | Saikia et al. (2021)  Krishnamoorthy et al. (2015) |
| 335 | Alloaromadendrene | *M. paniculata*  *M. exotica* | [C_15_H_24_](https://pubchem.ncbi.nlm.nih.gov/#query=C21H44) | 204.4 | 91354 | Hydrodistillation  Hydrodistillation | Leaves  Branches with leaves | Saikia et al. (2021)  You et al. (2015) |
| 336 | *α*-Caryophyllene | *M. paniculata*  *M. exotica* | [C_15_H_24_](https://pubchem.ncbi.nlm.nih.gov/#query=C21H44) | 204.4 |  | Hydrodistillation  Hydrodistillation | Leaves  Aerial parts | Saikia et al. (2021)  Li et al. (2010) |
| 337 | *β*-Cubebene | *M. paniculata*  *M. exotica* | [C_15_H_24_](https://pubchem.ncbi.nlm.nih.gov/#query=C21H44) | 204.4 | 93081 | Hydrodistillation Hydrodistillation | Leaves  Leaves | Saikia et al. (2021)  Pino et al. (2006) |
| 338 | Viridiflorene | *M. paniculata*  *M. exotica* | [C_15_H_24_](https://pubchem.ncbi.nlm.nih.gov/#query=C21H44) | 204.4 | 10910653 | Hydrodistillation  Hydrodistillation | Leaves  Leaves, twigs | Saikia et al. (2021)  Lv et al. (2013) |
| 339 | *δ*-Cadinene | *M. paniculata*  *M. exotica* | [C_15_H_24_](https://pubchem.ncbi.nlm.nih.gov/#query=C21H44) | 204.4 | 441005 | Hydrodistillation  Hydrodistillation | Leaves  Branches with leaves | Saikia et al. (2021)  You et al. (2015) |
| 340 | Nerolidol | *M. paniculata*  *M. exotica* | C_15_H_26_O | 222.4 | 5284507 | Hydrodistillation  Hydrodistillation | Leaves  Leaves | Saikia et al. (2021)  Pino et al. (2006) |
| 341 | Spathulenol | *M. paniculata*  *M. exotica* | C_15_H_24_O | 220.4 | 92231 | Hydrodistillation  Hydrodistillation | Leaves  Aerial parts | Saikia et al. (2021)  Li et al. (2010) |
| 342 | Guaiol | *M. paniculata*  *M. exotica* | C_15_H_26_O | 222.4 | 227829 | Hydrodistillation  Hydrodistillation | Leaves  Leaves, twigs | Saikia et al. (2021)  Lv et al. (2013) |
| 343 | Rosifoliol | *M. paniculata*  *M. exotica* | C_15_H_26_O | 222.4 | 527256 | Hydrodistillation  Hydrodistillation | Leaves  Leaves, twigs | Saikia et al. (2021)  Lv et al. (2013) |
| 344 | *τ*-Cadinol | *M. paniculata*  *M. exotica* | C_15_H_26_O | 222.4 | 160799 | Hydrodistillation  n-Hexane | Leaves  Flowers | Saikia et al. (2021)  Naseem et al. (2015) |
| 345 | N-hexadecanoic acid | *M. paniculata*  *M. exotica* | C_16_H_32_O_2_ | 256.4 | 985 | Hydrodistillation  n-Hexane | Leaves  Flowers | Saikia et al. (2021)  Naseem et al. (2015) |
| 346 | Benzaldehyde | *M. paniculata*  *M. exotica* | C_7_H_6_O | 106.1 | 240 | -  Hydrodistillation | Flowers  Flowers | Paul et al. (2020)  Huang et al. (2013) |
| 347 | 6-Methyl-5-hepten-2-one | *M. paniculata*  *M. exotica* | C_8_H_14_O | 126.2 | 9862 | -  Hydrodistillation | Flowers  Leaves | Paul et al. (2020)  Pino et al. (2006) |
| 348 | Methyl benzoate | *M. paniculata*  *M. exotica* | C_8_H_8_O_2_ | 136.2 | 7150 | -  n-Hexane | Flowers  Flowers | Paul et al. (2020)  Naseem et al. (2015) |
| 349 | Linalool | *M. paniculata*  *M. exotica* | C_10_H_18_O | 154.3 | 6549 | -  n-Hexane | Flowers  Flowers | Paul et al. (2020)  Naseem et al. (2015) |
| 350 | Nonanal | *M. paniculata*  *M. exotica* | C_9_H_18_O | 142.2 | 31289 | -  Hydrodistillation | Flowers  Flowers | Paul et al. (2020)  Huang et al. (2013) |
| 351 | 2-Phenylethanol | *M. paniculata*  *M. exotica* | C_8_H_10_O | 122.2 | 6054 | -  n-Hexane | Flowers  Flowers | Paul et al. (2020)  Naseem et al. (2015) |
| 352 | Methyl salicylate | *M. paniculata*  *M. exotica* | C_8_H_8_O_3_ | 152.2 | 4133 | -  n-Hexane | Flowers  Flowers | Paul et al. (2020)  Naseem et al. (2015) |
| 353 | Decanal | *M. paniculata*  *M. exotica* | C_10_H_20_O | 156.3 | 8175 | -  Hydrodistillation | Flowers  Leaves | Paul et al. (2020)  Pino et al. (2006) |
| 354 | Indole | *M. paniculata*  *M. exotica* | C_8_H_7_N | 117.2 | 798 | -  n-Hexane | Flowers  Flowers | Paul et al. (2020)  Naseem et al. (2015) |
| 355 | Methyl anthranilate | *M. paniculata*  *M. exotica* | C_8_H_9_NO_2_ | 151.2 | 8635 | -  n-Hexane | Flowers  Flowers | Paul et al. (2020)  Naseem et al. (2015) |
| 356 | (*E*,*E*)-*α*-Farnesene | *M. paniculata*  *M. exotica* | C_15_H_24_ | 204.4 | 5281516 | -  n-Hexane | Flowers  Flowers | Paul et al. (2020)  Naseem et al. (2015) |
| 357 | Benzyl benzoate | *M. paniculata*  *M. exotica* | C_14_H_12_O_2_ | 212.2 | 2345 | Hydrodistillation Hydrodistillation | Leaves  Leaves | Dosoky et al. (2016)  Krishnamoorthy et al. (2015) |
| 358 | Methyl palmitate | *M. paniculata*  *M. exotica* | C_17_H_34_O_2_ | 270.5 | 8181 | -  n-Hexane | Flowers  Flowers | Paul et al. (2020)  Naseem et al. (2015) |
| 359 | *β*- Bourbonene | *M. paniculata*  *M. exotica* | [C_15_H_24_](https://pubchem.ncbi.nlm.nih.gov/#query=C21H44) | 204.4 | 62566 | Hydrodistillation  Hydrodistillation | Leaves  Leaves | Solva et al. (2019)  Krishnamoorthy et al. (2015) |
| 360 | *β*-Humulene | *M. paniculata*  *M. exotica* | [C_15_H_24_](https://pubchem.ncbi.nlm.nih.gov/#query=C21H44) | 204.4 | 5318102 | Hydrodistillation Hydrodistillation | Leaves  Leaves | Solva et al. (2019)  Krishnamoorthy et al. (2015) |
| 361 | Germacrene B | *M. paniculata*  *M. exotica* | [C_15_H_24_](https://pubchem.ncbi.nlm.nih.gov/#query=C21H44) | 204.4 | 5281519 | Dichloromethane  Hydrodistillation | Leaves  Fresh leaves and flowers | Arya et al. (2017)  Raina et al. (2006) |
| 362 | *γ*-Elemene | *M. paniculata*  *M. exotica* | [C_15_H_24_](https://pubchem.ncbi.nlm.nih.gov/#query=C21H44) | 204.4 | 6432312 | Dichloromethane  Hydrodistillation | Leaves  Leaves, twigs | Arya et al. (2017)  Lv et al. (2013) |
| 363 | *β*-Cyclocitral | *M. paniculata*  *M. exotica* | [C_10_H_16_O](https://pubchem.ncbi.nlm.nih.gov/#query=C13H20O) | 152.2 | 9895 | Dichloromethane  Hydrodistillation | Leaves  Leaves, twigs | Arya et al. (2017)  Lv et al. (2013) |
| 364 | (-)-Cubenol | *M. paniculata*  *M. exotica* | C_15_H_26_O | 222.4 | 11770062 | Dichloromethane  Hydrodistillation | Leaves  Leaves, twigs | Arya et al. (2017)  Lv et al. (2013) |
| 365 | *a*-Curcumene | *M. paniculata*  *M. exotica* | C_15_H_22_ | 202.3 | 92139 | Dichloromethane  Hydrodistillation | Leaves  Branches with leaves | Arya et al. (2017)  You et al. (2015) |
| 366 | *β*-Selinene | *M. paniculata*  *M. exotica* | [C_15_H_24_](https://pubchem.ncbi.nlm.nih.gov/#query=C21H44) | 204.4 | 442393 | Dichloromethane  Hydrodistillation | Leaves  Leaves, twigs | Arya et al. (2017)  Lv et al. (2013) |
| 367 | *α*-Cadinene | *M. paniculata*  *M. exotica* | [C_15_H_24_](https://pubchem.ncbi.nlm.nih.gov/#query=C21H44) | 204.4 | 12306048 | Dichloromethane  Hydrodistillation | Leaves  Leaves, twigs | Arya et al. (2017)  Lv et al. (2013) |
| 368 | 2-Phenylethanal | *M. paniculata*  *M. exotica* | C_8_H_8_O | 120.2 | 998 | -  Hydrodistillation | Flowers  Leaves, fruits, flowers | Paul et al. (2020)  El-Sakhawy et al. (1998) |
| 369 | *α*-Bisabolol | *M. paniculata*  *M. exotica* | C_15_H_26_O | 222.4 | 1549992 | Dichloromethane  Hydrodistillation | Leaves  Leaves | Arya et al. (2017)  Krishnamoorthy et al. (2015) |
| 370 | *epi*-Cubenol | *M. paniculata*  *M. exotica* | C_15_H_26_O | 222.4 | 12046149 | Dichloromethane  Hydrodistillation | Leaves  Leaves, twigs | Arya et al. (2017)  Pino et al. (2006) |
| 371 | *β*-Eudesmol | *M. paniculata*  *M. exotica* | C_15_H_26_O | 222.4 | 91457 | Dichloromethane  Hydrodistillation | Leaves  Leaves, twigs | Arya et al. (2017)  Lv et al. (2013) |
| 372 | *α*-Muurolol | *M. paniculata*  *M. exotica* | C_15_H_26_O | 222.4 | 91753440 | Dichloromethane  Hydrodistillation | Leaves  Leaves, twigs | Arya et al. (2017)  Lv et al. (2013) |
| 373 | (*E*,*E*)-Farnesol | *M. paniculata*  *M. exotica* | C_15_H_26_O | 222.4 | 445070 | Dichloromethane  Hydrodistillation | Leaves  Leaves, flowers | Arya et al. (2017)  Raina et al. (2006) |
| 374 | (*E*,6)-Farnesol | *M. paniculata*  *M. exotica* | [C_15_H_26_](https://pubchem.ncbi.nlm.nih.gov/#query=C21H44)O | 222.4 | 1549109 | Hydrodistillation  Hydrodistillation | Leaves  Leaves, flowers | Dosoky et al. (2016)  Raina, et al. (2006) |
| 375 | *α*-Pinene | *M. paniculata*  *M. exotica* | [C_10_H_162_](https://pubchem.ncbi.nlm.nih.gov/#query=C10H16O2) | 136.2 | 6654 | Hydrodistillation  Hydrodistillation | Leaves  Fresh flowers, leaves ,fruits | Dosoky et al. (2016)  El-Sakhawy et al. (1998) |
| 376 | *α*-(*E*)-Bergamotene | *M. paniculata*  *M. exotica* | [C_15_H_24_](https://pubchem.ncbi.nlm.nih.gov/#query=C21H44) | 204.4 | 6429302 | Hydrodistillation  Hydrodistillation | Leaves, twigs  Leaves, flowers | Lv et al. (2013)  Raina et al. (2006) |
| 377 | (*E*)-*β*-Farnesene | *M. paniculata*  *M. exotica* | [C_15_H_24_](https://pubchem.ncbi.nlm.nih.gov/#query=C21H44) | 204.4 | 5281517 | Hydrodistillation  Hydrodistillation | Leaves, twigs  Leaves, flowers | Lv et al. (2013)  Raina et al. (2006) |
| 378 | *γ*-Cadinene | *M. paniculata*  *M. exotica* | [C_15_H_24_](https://pubchem.ncbi.nlm.nih.gov/#query=C21H44) | 204.4 | 6432404 | Hydrodistillation  Hydrodistillation | Leaves  Branches with leaves | Rodríguez et al. (2012)  You et al. (2015) |
| 379 | Cubebol | *M. paniculata*  *M. exotica* | C_15_H_26_O | 222.4 | 11276107 | Hydrodistillation  Hydrodistillation | Leaves, fruits  Leaves | Olawore et al. (2005)  Pino et al. (2006) |
| 380 | *α*-Elemol | *M. paniculata*  *M. exotica* | C_15_H_26_O | 222.4 | 92138 | Hydrodistillation  Hydrodistillation | Leaves, twigs  Leaves | Lv et al. (2013)  Pino et al. (2006) |
| 381 | Viridiflorol | *M. paniculata*  *M. exotica* | C_15_H_26_O | 222.4 | 11996452 | Hydrodistillation  Hydrodistillation | Leaves  Fresh leaves and flowers | Rodríguez et al. (2012)  Raina et al. (2006) |
| 382 | *α*-Terpineol | *M. paniculata*  *M. exotica* | C_10_H_18_O | 154.3 | 17100 | Hydrodistillation  Hydrodistillation | Leaves  Leaves, flowers | Dosoky et al. (2016)  Raina et al. (2006) |
| 383 | Geraniol | *M. paniculata*  *M. exotica* | C_10_H_18_O | 154.3 | 637566 | Hydrodistillation  Hydrodistillation | Leaves  Leaves, twigs | Dosoky et al. (2016)  Lv et al. (2013) |
| 384 | *p*-Vinylguaiacol | *M. paniculata*  *M. exotica* | C_9_H_10_O_2_ | 150.2 | 332 | Hydrodistillation  Hydrodistillation | Leaves  Aerial parts | Dosoky et al. (2016)  Li et al. (2010) |
| 385 | (*Z*)-Jasmone | *M. paniculata*  *M. exotica* | C_11_H_16_O | 164.3 | 1549018 | Hydrodistillation  n-Hexane | Leaves  Flowers | Dosoky et al. (2016)  Naseem et al. (2015) |
| 386 | *cis*-Murrola-3,5-diene | *M. paniculata*  *M. exotica* | [C_15_H_24_](https://pubchem.ncbi.nlm.nih.gov/#query=C21H44) | 204.4 | 51351708 | Hydrodistillation  Hydrodistillation | Leaves  Leaves, twigs | Dosoky et al. (2016)  Lv et al. (2013) |
| 387 | *α*-Selinene | *M. paniculata*  *M. exotica* | [C_15_H_24_](https://pubchem.ncbi.nlm.nih.gov/#query=C21H44) | 204.4 | 10856614 | Hydrodistillation  Hydrodistillation | Leaves  Leaves | Dosoky et al. (2016)  Krishnamoorthy et al. (2015) |
| 388 | Germacrene A | *M. paniculata*  *M. exotica* | [C_15_H_24_](https://pubchem.ncbi.nlm.nih.gov/#query=C21H44) | 204.4 | 9548705 | Hydrodistillation  Hydrodistillation | Leaves  Leaves | Dosoky et al. (2016)  Pino et al. (2006) |
| 389 | *β*-Sesquiphellandrene | *M. paniculata*  *M. exotica* | [C_15_H_24_](https://pubchem.ncbi.nlm.nih.gov/#query=C21H44) | 204.4 | 12315492 | Hydrodistillation  Hydrodistillation | Leaves  Branches with leaves | Dosoky et al. (2016)  You et al. (2015) |
| 390 | *trans*-Sesquisabinene hydrate | *M. paniculata*  *M. exotica* | C_15_H_26_O | 222.4 | 6428444 | Hydrodistillation  Hydrodistillation | Leaves  Leaves, flowers | Dosoky et al. (2016)  Raina et al. (2006) |
| 391 | Ethyl palmitate | *M. paniculata*  *M. exotica* | [C_19_H_34_](https://pubchem.ncbi.nlm.nih.gov/#query=C21H44)O_2_ | 294.5 | 5284421 | Hydrodistillation  Hydrodistillation | Leaves  Leaves | Dosoky et al. (2016)  Krishnamoorthy et al. (2015) |
| 392 | (*E*,*E*)-Geranyl linalool | *M. paniculata*  *M. exotica* | [C_20_H_34_](https://pubchem.ncbi.nlm.nih.gov/#query=C21H44)O | 290.5 | 5365872 | Hydrodistillation  Hydrodistillation | Leaves  Flowers | Dosoky et al. (2016)  Naseem et al. (2015) |
| 393 | Methyl linoleate | *M. paniculata*  *M. exotica* | [C_19_H_34_](https://pubchem.ncbi.nlm.nih.gov/#query=C21H44)O_2_ | 294.5 | 5284421 | Hydrodistillation  Hydrodistillation | Leaves  Leaves | Dosoky et al. (2016)  Krishnamoorthy et al. (2015) |
| 394 | Methyl linolenate | *M. paniculata*  *M. exotica* | [C_19_H_32_](https://pubchem.ncbi.nlm.nih.gov/#query=C21H44)O_2_ | 292.5 | 5319706 | Hydrodistillation Hydrodistillation | Leaves  Leaves | Dosoky et al. (2016)  Krishnamoorthy et al. (2015) |
| 395 | Phytol | *M. paniculata*  *M. exotica* | [C_20_H_40_](https://pubchem.ncbi.nlm.nih.gov/#query=C21H44)O | 296.5 | 5280435 | Hydrodistillation  Hydrodistillation | Leaves  Flowers | Dosoky et al. (2016)  Naseem et al. (2015) |
| 396 | Methyl stearate | *M. paniculata*  *M. exotica* | [C_19_H_38_](https://pubchem.ncbi.nlm.nih.gov/#query=C21H44)O_2_ | 298.5 | 8201 | Hydrodistillation  n-Hexane | Leaves  Flowers | Dosoky et al. (2016)  Naseem et al. (2015) |
| 397 | *α*-Terpinolene | *M. paniculata*  *M. exotica* | C_10_H_16_ | 136.2 | 11463 | Pentene  Hydrodistillation | Flowers  Leaves, flowers | Rout et al. (2007)  Raina et al. (2006) |
| 398 | Manool | *M. paniculata*  *M. exotica* | [C_20_H_34_](https://pubchem.ncbi.nlm.nih.gov/#query=C21H44)O | 290.5 | 3034394 | Pentane  Hydrodistillation | Flowers  Leaves | Rout et al. (2010)  Krishnamoorthy et al. (2015) |
| 399 | Docosane | *M. paniculata*  *M. exotica* | [C_22_H_46_](https://pubchem.ncbi.nlm.nih.gov/#query=C21H44)O | 310.6 | 12405 | Pentane  Hydrodistillation | Flowers  Leaves | Rout et al. (2010)  Krishnamoorthy et al. (2015) |
| 400 | Benzyldehyde | *M. paniculata*  *M. exotica* | [C_7_H_6_](https://pubchem.ncbi.nlm.nih.gov/#query=C21H44)O | 106.1 | 240 | Pentane  n-Hexane | Flowers  Flowers | Rout et al. (2007)  Naseem et al. (2015) |
| 401 | Myrcene | *M. paniculata*  *M. exotica* | [C_10_H_16_](https://pubchem.ncbi.nlm.nih.gov/#query=C21H44) | 136.2 | 31253 | Pentane  n-Hexane | Flowers  Flowers | Rout et al. (2007)  Naseem et al. (2015) |
| 402 | Limonene | *M. paniculata*  *M. exotica* | [C_10_H_16_](https://pubchem.ncbi.nlm.nih.gov/#query=C21H44) | 136.2 | 22311 | Hydrodistillation  n-Hexane | Leaves  Flowers | Chowdhury et al. (2008)  Naseem et al. (2015) |
| 403 | Ocimene | *M. paniculata*  *M. exotica* | [C_10_H_16_](https://pubchem.ncbi.nlm.nih.gov/#query=C21H44) | 136.2 | 18756 | Hydrodistillation  n-Hexane | Leaves  Flowers | Chowdhury et al. (2008)  Naseem et al. (2015) |
| 404 | Ethyl benzoate | *M. paniculata*  *M. exotica* | [C_9_H_10_](https://pubchem.ncbi.nlm.nih.gov/#query=C21H44)O_2_ | 150.2 | 7165 | Pentane  n-Hexane | Flowers  Flowers | Rout et al. (2010)  Naseem et al. (2015) |
| 405 | 2-Phenyl ethyl acetate | *M. paniculata*  *M. exotica* | [C_10_H_1_](https://pubchem.ncbi.nlm.nih.gov/#query=C21H44)_2_O_2_ | 164.2 | 7590 | Pentane  n-Hexane | Flowers  Flowers | Rout et al. (2010)  Naseem et al. (2015) |
| 406 | Anthranilic acid | *M. paniculata*  *M. exotica* | [C_7_H_14_NO_2_](https://pubchem.ncbi.nlm.nih.gov/#query=C21H44) | 137.1 | 227 | Pentane  n-Hexane | Flowers  Flowers | Rout et al. (2010)  Naseem et al. (2015) |
| 407 | 2-Methyl, 5-isopropylphenol; carvacrol | *M. paniculata*  *M. exotica* | C_10_H_14_O | 150.2 | 10364 | n-Hexane  Hydrodistillation | Aerial parts  Leaves, flowers | Shah et al. (2014)  Raina et al. (2006) |
| 408 | Octadecanoic acid; stearic acid | *M. paniculata*  *M. exotica* | C_18_H_36_O_2_ | 284.5 | 5281 | n-Hexane  Hydrodistillation | Aerial parts  Leaves, flowers | Shah et al. (2014)  Raina et al. (2006) |
| 409 | Tetradecanoic acid | *M. paniculata*  *M. exotica* | C_14_H_18_O_2_ | 228.4 | 11005 | n-Hexane  Hydrodistillation | Aerial parts  Leaves, flowers | Shah et al. (2014)  Raina et al. (2006) |
| 410 | *cis*-*β*-Farnesene | *M. paniculata*  *M. exotica* | [C_15_H_24_](https://pubchem.ncbi.nlm.nih.gov/#query=C21H44) | 204.4 | 5317319 | Hydrodistillation  Hydrodistillation | Leaves, twigs  Leaves | Lv et al. (2013)  Huang et al. (2013) |
| 411 | D-Limonene | *M. paniculata*  *M. exotica* | C_10_H_16_ | 136.2 | 440917 | Hydrodistillation Hydrodistillation | Leaves  Leaves | Mehmood et al. (2012)  Huang et al. (2013) |
| 412 | *cis*-Lanceol | *M. paniculata*  *M. exotica* | [C_15_H_24_](https://pubchem.ncbi.nlm.nih.gov/#query=C21H44)O | 220.4 | 6536796 | Hydrodistillation  Hydrodistillation | Leaves  Leaves | Chowdhury et al. (2008)  Huang et al. (2013) |
| 413 | *Z*-*β*-Ocimene | *M. paniculata*  *M. exotica* | C_10_H_16_ | 136.2 | 5320250 | Hydrodistillation Hydrodistillation | Leaves, twigs  Leaves | Lv et al. (2013)  Pino et al. (2006) |
| 414 | *E*-*β*-Ocimene | *M. paniculata.*  *M. exotica* | [C_10_H_16_](https://pubchem.ncbi.nlm.nih.gov/#query=C21H44) | 136.2 | 5281553 | Pentane Hydrodistillation | Flowers  Leaves | Rout et al. (2010)  Pino et al. (2006) |
| 415 | *cis*-Linalool oxide | *M. paniculata*  *M. exotica* | [C_10_H_18_O_2_](https://pubchem.ncbi.nlm.nih.gov/#query=C21H44) | 170.3 | 6428573 | Hydrodistillation  Hydrodistillation | Leaves, twigs  Leaves, twigs | Lv et al. (2013)  Lv et al. (2013) |
| 416 | *trans*-Linalool oxide | *M. paniculata*  *M. exotica* | [C_10_H_18_O_2_](https://pubchem.ncbi.nlm.nih.gov/#query=C21H44) | 170.3 | 6432254 | Hydrodistillation  Hydrodistillation | Leaves, twigs  Leaves, twigs | Lv et al. (2013)  Lv et al. (2013) |
| 417 | Geijerene | *M. paniculata*  *M. exotica* | [C_12_H_18_](https://pubchem.ncbi.nlm.nih.gov/#query=C21H44) | 162.3 | 12310053 | Hydrodistillation  Hydrodistillation | Leaves, twigs  Leaves, twigs | Lv et al. (2013)  Lv et al. (2013) |
| 418 | Iso-Methone | *M. paniculata*  *M. exotica* | C_10_H_18_O | 154.3 |  | Hydrodistillation  Hydrodistillation | Leaves, twigs  Leaves, twigs | Lv et al. (2013)  Lv et al. (2013) |
| 419 | Terpinen-4-ol | *M. paniculata*  *M. exotica* | C_10_H_18_O | 154.3 | 11230 | Hydrodistillation  Hydrodistillation | Leaves, twigs  Leaves, flowers | Lv et al. (2013)  Raina et al. (2006) |
| 420 | Cyclosativene | *M. paniculata*  *M. exotica* | [C_15_H_24_](https://pubchem.ncbi.nlm.nih.gov/#query=C21H44) | 204.4 | 519960 | Pentane  Hydrodistillation | Flowers  Leaves, twigs | Rout et al. (2010)  Lv et al. (2013) |
| 421 | *Z*-3-hexenyl acetate | *M. paniculata*  *M. exotica* | [C_8_H_14_](https://pubchem.ncbi.nlm.nih.gov/#query=C21H44)O_2_ | 142.2 | 5363388 | Pentane  Hydrodistillation | Flowers  Leaves | Rout et al. (2010)  Pino et al. (2006) |
| 422 | *γ*-Terpinene | *M. paniculata*  *M. exotica* | C_10_H_16_ | 136.2 | 7461 | Hydrodistillation Hydrodistillation | Leaves, flowers  Leaves, fruits | Raina et al. (2006)  Olawore et al. (2005) |
| 423 | Sabinene | *M. paniculata*  *M. exotica* | C_10_H_16_ | 136.2 | 18818 | Hydrodistillation  Hydrodistillation | Leaves  Leaves | Chowdhury et al. (2008)  Pino et al. (2006) |
| 424 | Globulol | *M. paniculata*  *M. exotica* | C_15_H_26_O | 222.4 | 12304985 | Hydrodistillation  Hydrodistillation | Leaves  Leaves, flowers | Chowdhury et al. (2008)  Raina et al. (2006) |
| 425 | Sibirene | *M. paniculata*  *M. exotica* | C_10_H_18_O | 154.3 | 14167644 | Hydrodistillation  Hydrodistillation | Leaves, twigs  Leaves, twigs | Lv et al. (2013)  Lv et al. (2013) |
| 426 | *α*-Guaiene | *M. paniculata*  *M. exotica* | [C_15_H_24_](https://pubchem.ncbi.nlm.nih.gov/#query=C21H44) | 204.4 | 5317844 | Hydrodistillation  Hydrodistillation | Leaves, twigs  Leaves, twigs | Lv et al. (2013)  Lv et al. (2013) |
| 427 | 6,9-Guaiadiene | *M. paniculata*  *M. exotica* | [C_15_H_24_](https://pubchem.ncbi.nlm.nih.gov/#query=C21H44) | 204.4 | 527113 | Hydrodistillation  Hydrodistillation | Leaves, twigs  Leaves, twigs | Lv et al. (2013)  Lv et al. (2013) |
| 428 | allo-Aromadendrene | *M. paniculata*  *M. exotica* | [C_15_H_24_](https://pubchem.ncbi.nlm.nih.gov/#query=C21H44) | 204.4 | 42608158 | Hydrodistillation  Hydrodistillation | Leaves, twigs  Leaves, twigs | Lv et al. (2013)  Lv et al. (2013) |
| 429 | *δ*-Selinene | *M. paniculata*  *M. exotica* | [C_15_H_24_](https://pubchem.ncbi.nlm.nih.gov/#query=C21H44) | 204.4 | 12308846 | Hydrodistillation  Hydrodistillation | Leaves  Leaves, twigs | Mehmood et al. (2012)  Lv et al. (2013) |
| 430 | *cis*-Calamenene | *M. paniculata*  *M. exotica* | [C_15_H_22_](https://pubchem.ncbi.nlm.nih.gov/#query=C21H44) | 202.4 | 6429077 | Hydrodistillation  Hydrodistillation | Leaves, twigs  Leaves, flowers | Lv et al. (2013)  Raina et al. (2006) |
| 431 | *β*-Oplopenone | *M. paniculata*  *M. exotica* | [C_15_H_24_](https://pubchem.ncbi.nlm.nih.gov/#query=C21H44)O | 220.4 | 14038847 | Hydrodistillation  Hydrodistillation | Leaves, twigs  Leaves, twigs | Lv et al. (2013)  Lv et al. (2013) |
| 432 | Ledol | *M. paniculata*  *M. exotica* | [C_15_H_26_](https://pubchem.ncbi.nlm.nih.gov/#query=C21H44)O | 222.4 | 92812 | Hydrodistillation  Hydrodistillation | Leaves  Leaves, twigs | Chowdhury et al. (2008)  Lv et al. (2013) |
| 433 | Humulene epoxide II | *M. paniculata*  *M. exotica* | [C_15_H_24_](https://pubchem.ncbi.nlm.nih.gov/#query=C21H44)O | 220.4 | 10704181 | Hydrodistillation  Hydrodistillation | Leaves, twigs  Leaves, twigs | Lv et al. (2013)  Lv et al. (2013) |
| 434 | 1-*epi*-Cubenol | *M. paniculata*  *M. exotica* | [C_15_H_24_](https://pubchem.ncbi.nlm.nih.gov/#query=C21H44)O | 220.4 | 519857 | Hydrodistillation  Hydrodistillation | Leaves, twigs  Leaves, twigs | Lv et al. (2013)  Lv et al. (2013) |
| 435 | Muurola-4,10(14)-diene-1-*β*-ol | *M. paniculata*  *M. exotica* | [C_15_H_24_](https://pubchem.ncbi.nlm.nih.gov/#query=C21H44)O | 220.4 | 6429089 | Hydrodistillation  Hydrodistillation | Leaves, twigs  Leaves, twigs | Lv et al. (2013)  Lv et al. (2013) |
| 436 | *α*-Calacorene | *M. paniculata*  *M. exotica* | [C_15_H_20_](https://pubchem.ncbi.nlm.nih.gov/#query=C21H44) | 200.3 | 12302243 | Hydrodistillation  Hydrodistillation | Leaves, twigs  Leaves, twigs | Lv et al. (2013)  Lv et al. (2013) |
| 437 | 5*E*,9*E-*Farnesyl acetone | *M. paniculata*  *M. exotica* | [C_18_H_30_](https://pubchem.ncbi.nlm.nih.gov/#query=C21H44)O | 262.4 | 1711945 | Hydrodistillation  Hydrodistillation | Leaves, twigs  Leaves, twigs | Lv et al. (2013)  Lv et al. (2013) |
| 438 | Cadalene | *M. paniculata*  *M. exotica* | [C_15_H_18_](https://pubchem.ncbi.nlm.nih.gov/#query=C21H44) | 198.3 | 10225 | Hydrodistillation  Hydrodistillation | Leaves, twigs  Leaves, twigs | Lv et al. (2013)  Lv et al. (2013) |
| 439 | Eudesma-4 (15),7-dien-1*β*-ol | *M. paniculata*  *M. exotica* | [C_15_H_24_](https://pubchem.ncbi.nlm.nih.gov/#query=C21H44)O | 220.4 |  | Hydrodistillation  Hydrodistillation | Leaves, twigs  Leaves, twigs | Lv et al. (2013)  Lv et al. (2013) |
| 440 | Isoledene | *M. paniculata*  *M. exotica* | [C_15_H_24_](https://pubchem.ncbi.nlm.nih.gov/#query=C21H44) | 204.4 | 530426 | Hydrodistillation Hydrodistillation | Leaves  Leaves, flowers | Mehmood et al. (2012)  Raina et al. (2006) |
| 441 | *α*-Phellandrene | *M. paniculata*  *M. exotica* | [C_10_H_16_](https://pubchem.ncbi.nlm.nih.gov/#query=C21H44) | 136.2 | 7460 | Hydrodistillation Hydrodistillation | Aerial parts  Leaves, fruits | Li et al. (2010)  Olawore et al. (2005) |
| 442 | *δ*-3-Carene | *M. paniculata*  *M. exotica* | [C_10_H_16_](https://pubchem.ncbi.nlm.nih.gov/#query=C21H44) | 136.2 | 26049 | Hydrodistillation Hydrodistillation | Aerial parts  Leaves, fruits | Li et al. (2010)  Olawore et al. (2005) |
| 443 | *ρ*-Cymene | *M. paniculata*  *M. exotica* | [C_10_H_14_](https://pubchem.ncbi.nlm.nih.gov/#query=C21H44) | 134.2 | 7463 | Hydrodistillation Hydrodistillation | Aerial parts  Leaves, fruits | Li et al. (2010)  Olawore et al. (2005) |
| 444 | Isogeijerin | *M. paniculata* | C_15_H_16_O_4_ | 260.3 | 605135 | n-Hexane | Aerial parts | Shah et al. (2014) |
| 445 | 4-(5-Methyl-2-furanyl), 2-butanone | *M. paniculata* | C_9_H_12_O_2_ | 152.2 | 524268 | n-Hexane | Aerial parts | Shah et al. (2014) |
| 446 | Phenylethyl tiglate | *M. paniculata* | C_13_H_16_O_2_ | 204.3 | 5357002 | Hydrodistillation | Leaves | Dosoky et al. (2016) |
| 447 | Spathulenol isomer | *M. paniculata* | [C_15_H_24_](https://pubchem.ncbi.nlm.nih.gov/#query=C21H44)O | 220.4 | 13854255 | Hydrodistillation | Leaves | Dosoky et al. (2016) |
| 448 | *β*-Nootkatol | *M. paniculata* | [C_15_H_24_](https://pubchem.ncbi.nlm.nih.gov/#query=C21H44)O | 220.4 | 182645 | Hydrodistillation | Leaves | Dosoky et al. (2016) |
| 449 | Zingiberenol | *M. paniculata* | [C_15_H_26_](https://pubchem.ncbi.nlm.nih.gov/#query=C21H44)O | 222.4 | 13213649 | Hydrodistillation | Leaves | Dosoky et al. (2016) |
| 450 | Selin-6-en-4-ol | *M. paniculata* | [C_15_H_26_](https://pubchem.ncbi.nlm.nih.gov/#query=C21H44)O | 222.4 |  | Hydrodistillation | Leaves | Dosoky et al. (2016) |
| 451 | Isospathulenol | *M. paniculata* | [C_15_H_24_](https://pubchem.ncbi.nlm.nih.gov/#query=C21H44)O | 220.4 | 102303030 | Hydrodistillation | Leaves | Dosoky et al. (2016) |
| 452 | *epi*-*β*-Muurolol | *M. paniculata* | [C_15_H_26_](https://pubchem.ncbi.nlm.nih.gov/#query=C21H44)O | 222.4 |  | Hydrodistillation | Leaves | Dosoky et al. (2016) |
| 453 | Intermedeol | *M. paniculata* | [C_15_H_26_](https://pubchem.ncbi.nlm.nih.gov/#query=C21H44)O | 222.4 | 15560333 | Hydrodistillation | Leaves | Dosoky et al. (2016) |
| 454 | Germacra-4(15),5,10(14)-trien-1*α*-ol | *M. paniculata* | [C_15_H_24_](https://pubchem.ncbi.nlm.nih.gov/#query=C21H44) | 204.4 |  | Hydrodistillation | Leaves | Dosoky et al. (2016) |
| 455 | Eudesma-4,11-dien-2-ol | *M. paniculata* | [C_15_H_24_](https://pubchem.ncbi.nlm.nih.gov/#query=C21H44)O | 220.4 | 91750419 | Hydrodistillation | Leaves | Dosoky et al. (2016) |
| 456 | Nuciferol | *M. paniculata* | [C_15_H_22_](https://pubchem.ncbi.nlm.nih.gov/#query=C21H44)O | 218.4 | 10932923 | Hydrodistillation | Leaves | Dosoky et al. (2016) |
| 457 | Oplopanone | *M. paniculata* | [C_15_H_26_](https://pubchem.ncbi.nlm.nih.gov/#query=C21H44)O_2_ | 238.4 | 10466745 | Hydrodistillation | Leaves | Dosoky et al. (2016) |
| 458 | Curcumen-12-ol | *M. paniculata* | [C_15_H_24_](https://pubchem.ncbi.nlm.nih.gov/#query=C21H44)O | 220.4 |  | Hydrodistillation | Leaves | Dosoky et al. (2016) |
| 459 | Isospathulenol isomer | *M. paniculata* | [C_15_H_24_](https://pubchem.ncbi.nlm.nih.gov/#query=C21H44)O | 220.4 |  | Hydrodistillation | Leaves | Dosoky et al. (2016) |
| 460 | Phenylethyl salicylate | *M. paniculata* | [C_15_H_14_](https://pubchem.ncbi.nlm.nih.gov/#query=C21H44)O_3_ | 242.3 | 62332 | Hydrodistillation | Leaves | Dosoky et al. (2016) |
| 461 | *α*-Ylangene | *M. paniculata* | [C_15_H_24_](https://pubchem.ncbi.nlm.nih.gov/#query=C21H44) | 204.4 | 442409 | Hydrodistillation | Unripe and ripe fruits | Silva et al. (2020) |
| 462 | Sesquithujene | *M. paniculata* | [C_15_H_24_](https://pubchem.ncbi.nlm.nih.gov/#query=C21H44) | 204.4 | 25147318 | Hydrodistillation | Unripe and ripe fruits | Silva et al. (2020) |
| 463 | *β*-Bisabolene | *M. paniculata* | [C_15_H_24_](https://pubchem.ncbi.nlm.nih.gov/#query=C21H44) | 204.4 | 10104370 | Hydrodistillation | Unripe and ripe fruits | Silva et al. (2020) |
| 464 | Lauryl acetate | *M. paniculata* | [C_14_H_28_](https://pubchem.ncbi.nlm.nih.gov/#query=C21H44)O_2_ | 228.4 | 8205 | Hydrodistillation | Unripe and ripe fruits | Silva et al. (2020) |
| 465 | 2-Methylbutanoate | *M. paniculata* | [C_5_H_9_](https://pubchem.ncbi.nlm.nih.gov/#query=C21H44)O_2_ | 101.1 | 22253297 | Hydrodistillation | Unripe and ripe fruits | Silva et al. (2020) |
| 466 | Isovaleric acid, decyl ester | *M. paniculata* | [C_15_H_30_](https://pubchem.ncbi.nlm.nih.gov/#query=C21H44)O_2_ | 242.4 | 166371 | Hydrodistillation | Unripe and ripe fruits | Silva et al. (2020) |
| 467 | Elixene | *M. paniculata* | [C_15_H_24_](https://pubchem.ncbi.nlm.nih.gov/#query=C21H44) | 204.4 | 94254 | Hydrodistillation | Leaves | Saikia et al. (2021) |
| 468 | Patchoulane | *M. paniculata* | [C_15_H_26_](https://pubchem.ncbi.nlm.nih.gov/#query=C21H44) | 206.4 | 29408 | Hydrodistillation | Leaves | Saikia et al. (2021) |
| 469 | 3,3'-Bi-*p*-menthane | *M. paniculata* | C_20_H_28_ | 278.5 | 557970 | Hydrodistillation | Leaves | Saikia et al. (2021) |
| 470 | Benzyl alcohol | *M. paniculata* | C_7_H_8_O | 108.1 | 244 | - | Flowers | Paul et al. (2020) |
| 471 | Benzyl nitrile | *M. paniculata* | C_8_H_7_N | 117.2 | 8794 | - | Flowers | Paul et al. (2020) |
| 472 | - 2-Aminobenzaldehyde | *M. paniculata* | C_7_H_7_NO | 121.1 | 68255 | - | Flowers | Paul et al. (2020) |
| 473 | Bicyclo[5.3.0]decane,  2-methylene-5-(1-methylvinyl)-8-methyl | *M. paniculata* | [C_15_H_24_](https://pubchem.ncbi.nlm.nih.gov/#query=C21H44) | 204.4 | 564533 | Hydrodistillation | Leaves | Saikia et al. (2021) |
| 474 | (+)-Cyclosativene | *M. paniculata* | C_15_H_24_ | 204.4 | 16212927 | - | Flowers | Paul et al. (2020) |
| 475 | *trans*-Verbenol | *M. paniculata* | [C_10_H_16_O](https://pubchem.ncbi.nlm.nih.gov/#query=C13H20O) | 152.2 | 89664 | Dichloromethane | Leaves | Arya et al. (2017) |
| 476 | *τ*-Caryophyllene | *M. paniculata* | C_14_H_22_ | 190.3 | 6708700 | Dichloromethane | Leaves | Arya et al. (2017) |
| 477 | Decyl senecioate | *M. paniculata* |  |  |  | Hydrodistillation | Unripe and ripe fruits | Silva et al. (2020) |
| 478 | Isovaleric acid. dodecyl ester | *M. paniculata* | [C_17_H_34_](https://pubchem.ncbi.nlm.nih.gov/#query=C21H44)O_2_ | 270.5 | 243803 | Hydrodistillation | Unripe and ripe fruits | Silva et al. (2020) |
| 479 | 5-Methyl thiazole | *M. paniculata* | C_4_H_5_NS | 99.2 | 137980 | Hydrodistillation | Leaves | Saikia et al. (2021) |
| 480 | Longifolene | *M. paniculata* | [C_15_H_24_](https://pubchem.ncbi.nlm.nih.gov/#query=C21H44) | 204.4 | 289151 | Hydrodistillation | Leaves | Saikia et al. (2021) |
| 481 | *γ*-Gurjunene | *M. paniculata* | [C_15_H_24_](https://pubchem.ncbi.nlm.nih.gov/#query=C21H44) | 204.4 | 90805 | Hydrodistillation | Leaves | Saikia et al. (2021) |
| 482 | Dehydro-aromadendrene | *M. paniculata* | [C_15_H_24_](https://pubchem.ncbi.nlm.nih.gov/#query=C21H44) | 204.4 | 91746711 | Hydrodistillation | Leaves | Saikia et al. (2021) |
| 483 | Isophytol | *M. paniculata* | C_20_H_40_O | 296.5 | 10453 | Hydrodistillation | Leaves | Saikia et al. (2021) |
| 484 | Bicycloelemene | *M. paniculata* | [C_15_H_24_](https://pubchem.ncbi.nlm.nih.gov/#query=C21H44) | 204.4 | 56842786 | Hydrodistillation | Unripe and ripe fruits | Silva et al. (2020) |
| 485 | Elemene isomer | *M. paniculata* | [C_15_H_24_](https://pubchem.ncbi.nlm.nih.gov/#query=C21H44) | 204.4 |  | Hydrodistillation | Unripe and ripe fruits | Silva et al. (2020) |
| 486 | *β*-Gurjunene | *M. paniculata* | [C_15_H_24_](https://pubchem.ncbi.nlm.nih.gov/#query=C21H44) | 204.4 | 6450812 | Hydrodistillation | Unripe and ripe fruits | Silva et al. (2020) |
| 487 | Isogermacrene D | *M. paniculata* | [C_15_H_24_](https://pubchem.ncbi.nlm.nih.gov/#query=C21H44) | 204.4 | 91723653 | Hydrodistillation | Unripe and ripe fruits | Silva et al. (2020) |
| 488 | Isogermacrene A | *M. paniculata* | [C_15_H_24_](https://pubchem.ncbi.nlm.nih.gov/#query=C21H44) | 204.4 | 91749707 | Hydrodistillation | Leaves, fruits | Olawore et al. (2005) |
| 489 | Cubeb-11-ene | *M. paniculata* | [C_15_H_24_](https://pubchem.ncbi.nlm.nih.gov/#query=C21H44) | 204.4 | 91747380 | Hydrodistillation | Leaves, fruits | Olawore et al. (2005) |
| 490 | *β*-Bisabolol | *M. paniculata* | C_15_H_26_O | 222.4 | 12300146 | Dichloromethane | Leaves | Arya et al. (2017) |
| 491 | 3-Methoxy, 4-hydroxy benzaldehyde | *M. paniculata* | C_8_H_8_O_3_ | 152.2 |  | n-Hexane | Aerial parts | Shah et al. (2014) |
| 492 | Tetrahydrodehydrogeijerin | *M. paniculata* | C_15_H_18_O_4_ | 262.3 | 609637 | n-Hexane | Aerial parts | Shah et al. (2014) |
| 493 | 6-Amino, 4-oxochromen-2-carboxylate | *M. paniculata* | C_12_H_11_NO_4_ | 233.2 | 616248 | n-Hexane | Aerial parts | Shah et al. (2014) |
| 494 | 2,4-Dihexyl, 7,7-dimethyl, 1,3,5-cycloheptatriene | *M. paniculata* | C_21_H_36_ | 233.2 | 608892 | n-Hexane | Aerial parts | Shah et al. (2014) |
| 495 | 3-Hydroxy, 3-nonyl-1H-quinoline-2,4-dione | *M. paniculata* | C_18_H_25_NO_3_ | 303.4 | 605393 | n-Hexane | Aerial parts | Shah et al. (2014) |
| 496 | Bis (2-ethylhexyl) phthalate | *M. paniculata* | C_24_H_38_O_4_ | 390.6 | 8343 | n-Hexane | Aerial parts | Shah et al. (2014) |
| 497 | 6-Methoxy, 2-vinyl, 9-[3-deoxyribofuranosyl] purine | *M. paniculata* | C_13_H_16_N_4_O_4_ | 292.3 |  | n-Hexane | Aerial parts | Shah et al. (2014) |
| 498 | 8-Amino, 6-methoxy, 4-methyl, 5-[n-nonoxy] quinoline | *M. paniculata* | C_20_H_30_N_2_O_2_ | 330.5 | 608899 | n-Hexane | Aerial parts | Shah et al. (2014) |
| 499 | Myristoylolean-12-en-28-ol | *M. paniculata* | C_44_H_76_O_3_ | 653.1 | 609052 | n-Hexane | Aerial parts | Shah et al. (2014) |
| 500 | Dimethyl nonanedioate | *M. paniculata* | C_11_H_20_O_4_ | 216.3 | 15612 | n-Hexane | Aerial parts | Shah et al. (2014) |
| 501 | Ethyl 4-hydroxy, 3-methoxybenzoate | *M. paniculata* | C_10_H_12_O_4_ | 196.2 | 12038 | n-Hexane | Aerial parts | Shah et al. (2014) |
| 502 | 4,7-Dimethoxy, 5-(2-propenyl), 1,3-benzodioxole | *M. paniculata* | C_12_H_14_O_4_ | 222.2 | 10659 | n-Hexane | Aerial parts | Shah et al. (2014) |
| 503 | Methyl tetradecanoate | *M. paniculata* | C_15_H_30_O_2_ | 242.4 | 31284 | n-Hexane | Aerial parts | Shah et al. (2014) |
| 504 | Methyl 12-methyltetradecanoate | *M. paniculata* | C_16_H_32_O_2_ | 256.4 | 21206 | n-Hexane | Aerial parts | Shah et al. (2014) |
| 505 | 6,10,14-Trimethyl, 2-pentadecanone | *M. paniculata* | C_18_H_36_O | 268.5 | 10408 | n-Hexane | Aerial parts | Shah et al. (2014) |
| 506 | Methyl 14-methylpentadecanoate | *M. paniculata* | C_17_H_34_O_2_ | 270.5 | 21205 | n-Hexane | Aerial parts | Shah et al. (2014) |
| 507 | Methyl 14-methylhexadecanoate | *M. paniculata* | C_18_H_36_O_2_ | 284.5 | 520159 | n-Hexane | Aerial parts | Shah et al. (2014) |
| 508 | Methyl 16-methylheptadecanoate | *M. paniculata* | C_19_H_38_O_2_ | 298.5 | 110444 | n-Hexane | Aerial parts | Shah et al. (2014) |
| 509 | Methyl 10-octadecenoate | *M. paniculata* | C_19_H_36_O_2_ | 296.5 | 25642 | n-Hexane | Aerial parts | Shah et al. (2014) |
| 510 | 9-Octadecenoic acid | *M. paniculata* | C_18_H_34_O_2_ | 282.5 | 637517 | n-Hexane | Aerial parts | Shah et al. (2014) |
| 511 | Habranthine | *M. paniculata* | C_17_H_21_NO_4_ | 303.4 | 615376 | n-Hexane | Aerial parts | Shah et al. (2014) |
| 512 | 3-Methoxy, 5-(2-phenylethenyl), diacetate 1,2-benzenediol | *M. paniculata* | C_19_H_18_O_5_ | 326.3 | 90474715 | n-Hexane | Aerial parts | Shah et al. (2014) |
| 513 | Methyl tetracosanoate | *M. paniculata* | C_25_H_50_O_2_ | 382.7 | 75546 | n-Hexane | Aerial parts | Shah et al. (2014) |
| 514 | 4-Angeloyloxypruteninone | *M. paniculata* | C_25_H_30_O_7_ | 442.5 | 5367747 | n-Hexane | Aerial parts | Shah et al. (2014) |
| 515 | 6-(*p*-Tolyl), 2-methyl hepten-2-ol | *M. paniculata* | C_15_H_22_O | 218.3 | 69775937 | n-Hexane | Aerial parts | Shah et al. (2014) |
| 516 | 5-Octadecenal | *M. paniculata* | C_18_H_34_O | 266.5 | 545652 | n-Hexane | Aerial parts | Shah et al. (2014) |
| 517 | Heptadecanoic acid | *M. paniculata* | C_17_H_34_O_2_ | 270.5 | 10465 | n-Hexane | Aerial parts | Shah et al. (2014) |
| 518 | 3,7,11,15-Tetramethyl, 2-hexadecen-1-ol | *M. paniculata* | C_20_H_40_O | 296.5 | 5366244 | n-Hexane | Aerial parts | Shah et al. (2014) |
| 519 | Butyl cyclohexyl phthalate | *M. paniculata* | C_18_H_24_O_4_ | 304.4 | 6779 | n-Hexane | Aerial parts | Shah et al. (2014) |
| 520 | 10-Hydroxy, 5,7dimethoxy, 2,3dimethyl, 1,4anthracenedione | *M. paniculata* | C_18_H_16_O_5_ | 296.4 |  | n-Hexane | Aerial parts | Shah et al. (2014) |
| 521 | Cholestane-3,6,7-triol | *M. paniculata* | C_27_H_48_O_3_ | 420.7 | 22296770 | n-Hexane | Aerial parts | Shah et al. (2014) |
| 522 | 4-(4-Pentylcyclohexyl), 4'-(4-propyl-1-  cyclohexen-1-yl) 1,1'-biphenyl | *M. paniculata* | C_32_H_44_ | 428.7 | 634697 | n-Hexane | Aerial parts | Shah et al. (2014) |
| 523 | (3*Z*)-Hexenyl benzoate | *M. paniculata* | C_13_H_16_O_2_ | 204.3 | 5367706 | Hydrodistillation | Leaves | Dosoky et al. (2016) |
| 524 | *α*-Chamigrene | *M. paniculata* | [C_15_H_24_](https://pubchem.ncbi.nlm.nih.gov/#query=C21H44) | 204.4 | 442351 | Hydrodistillation | Leaves | Dosoky et al. (2016) |
| 525 | Isogeigerin | *M. paniculata* | [C_15_H_20_](https://pubchem.ncbi.nlm.nih.gov/#query=C21H44)O_4_ | 264.3 |  | Hydrodistillation | Leaves | Dosoky et al. (2016) |
| 526 | Suberosin epoxide | *M. paniculata* | [C_15_H_16_](https://pubchem.ncbi.nlm.nih.gov/#query=C21H44)O_4_ | 260.3 | 612480 | Hydrodistillation | Leaves | Dosoky et al. (2016) |
| 527 | Octyl palmitate | *M. paniculata* | [C_24_H_48_](https://pubchem.ncbi.nlm.nih.gov/#query=C21H44)O_2_ | 368.6 | 85651 | Hydrodistillation | Leaves | Dosoky et al. (2016) |
| 528 | Octyl stearate | *M. paniculata* | [C_26_H_52_](https://pubchem.ncbi.nlm.nih.gov/#query=C21H44)O_2_ | 396.7 | 30916 | Hydrodistillation | Leaves | Dosoky et al. (2016) |
| 529 | Squalene | *M. paniculata* | [C_30_H_50_](https://pubchem.ncbi.nlm.nih.gov/#query=C21H44) | 410.7 | 638072 | Hydrodistillation | Leaves | Dosoky et al. (2016) |
| 530 | *γ*-Eudesmol | *M. paniculata* | C_15_H_26_O | 222.4 | 6432005 | Dichloromethane | Leaves | Arya et al. (2017) |
| 531 | *n*-Undecane | *M. paniculata* | C_11_H_24_ | 156.3 | 14257 | Hydrodistillation | Leaves, twigs | Lv et al. (2013) |
| 532 | Menthone | *M. paniculata* | C_10_H_18_O | 154.3 | 26447 | Hydrodistillation | Leaves, twigs | Lv et al. (2013) |
| 533 | Menthol | *M. paniculata* | C_10_H_20_O | 156.3 | 1254 | Hydrodistillation | Leaves, twigs | Lv et al. (2013) |
| 534 | *n*-Dodecane | *M. paniculata* | C_12_H_26_ | 170.3 | 8182 | Hydrodistillation | Leaves, twigs | Lv et al. (2013) |
| 535 | *n*-Tridecane | *M. paniculata* | C_13_H_28_ | 184.4 | 12388 | Hydrodistillation | Leaves, twigs | Lv et al. (2013) |
| 536 | Carvenone | *M. paniculata* | C_10_H_16_O | 152.2 | 10363 | Hydrodistillation | Leaves, twigs | Lv et al. (2013) |
| 537 | 7-*epi*-Sesquithujene | *M. paniculata* | [C_15_H_24_](https://pubchem.ncbi.nlm.nih.gov/#query=C21H44) | 204.4 | 56927990 | Hydrodistillation | Leaves, twigs | Lv et al. (2013) |
| 538 | *n*-Tetradecane | *M. paniculata* | [C_14_H_30_](https://pubchem.ncbi.nlm.nih.gov/#query=C21H44) | 198.4 | 12389 | Hydrodistillation | Leaves, twigs | Lv et al. (2013) |
| 539 | Sesquisabinene | *M. paniculata* | [C_15_H_24_](https://pubchem.ncbi.nlm.nih.gov/#query=C21H44) | 204.4 | 25202482 | Hydrodistillation | Leaves, twigs | Lv et al. (2013) |
| 540 | *epi*-*α*-Bisabolol | *M. paniculata* | [C_15_H_26_](https://pubchem.ncbi.nlm.nih.gov/#query=C21H44)O | 222.4 | 1201551 | Hydrodistillation | Leaves, twigs | Lv et al. (2013) |
| 541 | *n*-Heptadecane | *M. paniculata* | [C_17_H_36_](https://pubchem.ncbi.nlm.nih.gov/#query=C21H44) | 240.5 | 12398 | Hydrodistillation | Leaves, twigs | Lv et al. (2013) |
| 542 | 14-Hydroxy-9-*epi*-(*E*)- caryophyllene | *M. paniculata* | [C_15_H_24_](https://pubchem.ncbi.nlm.nih.gov/#query=C21H44)O | 220.4 | 5352484 | Hydrodistillation | Leaves, twigs | Lv et al. (2013) |
| 543 | *β*-Macrocarpene | *M. paniculata* | [C_15_H_24_](https://pubchem.ncbi.nlm.nih.gov/#query=C21H44) | 204.4 | 49859620 | Hydrodistillation | Leaves, twigs | Lv et al. (2013) |
| 544 | *α*-Bulnesene | *M. paniculata* | [C_15_H_24_](https://pubchem.ncbi.nlm.nih.gov/#query=C21H44) | 204.4 | 94275 | Hydrodistillation | Leaves, twigs | Lv et al. (2013) |
| 545 | *δ*-Amorphene | *M. paniculata* | [C_15_H_24_](https://pubchem.ncbi.nlm.nih.gov/#query=C21H44) | 204.4 | 10223 | Hydrodistillation | Leaves, twigs | Lv et al. (2013) |
| 546 | Geranyl isobutanoate | *M. paniculata* | [C_14_H_24_](https://pubchem.ncbi.nlm.nih.gov/#query=C21H44)O_2_ | 224.3 | 5365991 | Hydrodistillation | Leaves, twigs | Lv et al. (2013) |
| 547 | Sesquicineole | *M. paniculata* | [C_15_H_24_](https://pubchem.ncbi.nlm.nih.gov/#query=C21H44) | 204.4 | 341779 | Hydrodistillation | Leaves, twigs | Lv et al. (2013) |
| 548 | *α*-Corocalene | *M. paniculata* | [C_15_H_20_](https://pubchem.ncbi.nlm.nih.gov/#query=C21H44) | 200.3 | 5316074 | Hydrodistillation | Leaves, twigs | Lv et al. (2013) |
| 549 | *α*-Eudesmol | *M. paniculata* | [C_15_H_26_](https://pubchem.ncbi.nlm.nih.gov/#query=C21H44)O | 222.4 | 92762 | Hydrodistillation | Leaves, twigs | Lv et al. (2013) |
| 550 | *γ*-Vetivenene | *M. paniculata* | [C_15_H_22_](https://pubchem.ncbi.nlm.nih.gov/#query=C21H44) | 202.4 | 90470826 | Hydrodistillation | Leaves, twigs | Lv et al. (2013) |
| 551 | Dodecanoic acid | *M. paniculata* | [C_12_H_24_](https://pubchem.ncbi.nlm.nih.gov/#query=C21H44)O_2_ | 200.3 | 3893 | Hydrodistillation | Leaves, twigs | Lv et al. (2013) |
| 552 | *trans*-Cadina-1,4-diene | *M. paniculata* | [C_15_H_24_](https://pubchem.ncbi.nlm.nih.gov/#query=C21H44) | 204.4 | 6430869 | Hydrodistillation | Leaves, twigs | Lv et al. (2013) |
| 553 | Sandaracopimara-8(14),15-diene | *M. paniculata* | [C_20_H_32_](https://pubchem.ncbi.nlm.nih.gov/#query=C21H44) | 272.5 | 440909 | Hydrodistillation | Leaves, twigs | Lv et al. (2013) |
| 554 | Kaurene | *M. paniculata* | [C_20_H_32_](https://pubchem.ncbi.nlm.nih.gov/#query=C21H44) | 272.5 | 5318786 | Hydrodistillation | Leaves, twigs | Lv et al. (2013) |
| 555 | Psi-cumene | *M. paniculata* | [C_9_H_12_](https://pubchem.ncbi.nlm.nih.gov/#query=C21H44) | 120.2 | 7247 | Hydrodistillation | Leaves | Mehmood et al. (2012) |
| 556 | (+)-3-Carene | *M. paniculata* | [C_10_H_16_](https://pubchem.ncbi.nlm.nih.gov/#query=C21H44) | 136.2 | 443156 | Hydrodistillation | Leaves | Mehmood et al. (2012) |
| 557 | Columbin | *M. paniculata* | [C_20_H_22_O_6_](https://pubchem.ncbi.nlm.nih.gov/#query=C21H44) | 358.4 | 442015 | Hydrodistillation | Leaves | Mehmood et al. (2012) |
| 558 | (-1)-4-Carene | *M. paniculata* | [C_10_H_16_](https://pubchem.ncbi.nlm.nih.gov/#query=C21H44) | 136.2 | 16211586 | Hydrodistillation | Leaves | Mehmood et al. (2012) |
| 559 | Azulene | *M. paniculata* | [C_10_H_8_](https://pubchem.ncbi.nlm.nih.gov/#query=C21H44) | 128.2 | 9231 | Hydrodistillation | Leaves | Mehmood et al. (2012) |
| 560 | Nerolidyl acetate | *M. paniculata* | [C_17_H_28_O_2_](https://pubchem.ncbi.nlm.nih.gov/#query=C21H44) | 264.4 | 5363426 | Hydrodistillation | Leaves | Mehmood et al. (2012) |
| 561 | 2-Nonynoic acid | *M. paniculata* | [C_9_H_14_](https://pubchem.ncbi.nlm.nih.gov/#query=C21H44)O_2_ | 204.4 | 74611 | Hydrodistillation | Leaves | Mehmood et al. (2012) |
| 562 | Undecanol | *M. paniculata* | [C_11_H_24_](https://pubchem.ncbi.nlm.nih.gov/#query=C21H44)O | 172.3 | 8184 | Hydrodistillation | Leaves | Mehmood et al. (2012) |
| 563 | 1,10-Di-*epi*-cubenol | *M. paniculata* | C_15_H_26_O | 222.4 |  | — | Leaves | Rodríguez et al. (2012) |
| 564 | 2-Hexanal | *M. paniculata* | [C_6_H_12_](https://pubchem.ncbi.nlm.nih.gov/#query=C21H44)O | 100.2 | 11583 | Pentane | Flowers | Rout et al. (2010) |
| 565 | *E*-3-Hexen-1-ol | *M. paniculata* | [C_6_H_12_](https://pubchem.ncbi.nlm.nih.gov/#query=C21H44)O | 100.2 | 5284503 | Pentane | Flowers | Rout et al. (2010) |
| 566 | *Z*-2-Hexen-1-ol | *M. paniculata* | [C_6_H_12_](https://pubchem.ncbi.nlm.nih.gov/#query=C21H44)O | 100.2 | 5324489 | Pentane | Flowers | Rout et al. (2010) |
| 567 | Phenyl acetonitrile | *M. paniculata* | C_8_H_7_N | 117.2 | 8794 | Pentane | Flowers | Rout et al. (2010) |
| 568 | Methyl phenylacetate | *M. paniculata* | C_9_H_10_O_2_ | 150.2 | 7559 | Pentane | Flowers | Rout et al. (2010) |
| 569 | Geranyl acetone | *M. paniculata* | C_13_H_22_O | 194.3 | 1549778 | Pentane | Flowers | Rout et al. (2010) |
| 570 | Phenyl ethyl tiglate | *M. paniculata* | C_13_H_16_O_2_ | 204.3 | 41552 | Pentane | Flowers | Rout et al. (2010) |
| 571 | Pentyl furan | *M. paniculata* | C_9_H_14_O | 138.2 | 19602 | Hydrodistillation | Leaves, fruits | Olawore et al. (2005) |
| 572 | Bicyclosesquiphellandrene | *M. paniculata* | C_15_H_24_ | 204.4 | 521496 | Hydrodistillation | Leaves, fruits | Olawore et al. (2005) |
| 573 | Benzyl phenyl acetate | *M. paniculata* | C_15_H_14_O_2_ | 226.3 | 60999 | Pentane | Flowers | Rout et al. (2010) |
| 574 | Tetracosane | *M. paniculata* | C_24_H_50_ | 338.7 | 12592 | Pentane | Flowers | Rout et al. (2010) |
| 575 | 9,12-Octadecadienol | *M. paniculata* | C_18_H_34_O | 266.5 | 5462912 | Pentane | Flowers | Rout et al. (2010) |
| 576 | Pentacosane | *M. paniculata* | C_25_H_52_ | 352.7 | 12406 | Pentane | Flowers | Rout et al. (2010) |
| 577 | Hexacosane | *M. paniculata* | C_26_H_54_ | 366.7 | 12407 | Pentane | Flowers | Rout et al. (2010) |
| 578 | Phenethyl benzoate | *M. paniculata* | C_15_H_14_O_2_ | 226.3 | 7194 | Pentane | Flowers | Rout et al. (2010) |
| 579 | 3-Hexen-1-ol, formate | *M. paniculata* | C_7_H_12_O_2_ | 128.2 | 6306732 | Hydrodistillation | Leaves | Chowdhury et al. (2008) |
| 580 | Cyclohexene, 3,4-diethenyl-3-methyl- | *M. paniculata* | C_11_H_16_ | 148.2 | 556488 | Hydrodistillation | Leaves | Chowdhury et al. (2008) |
| 581 | Cyclohexene, 5,6-diethenyl-3-methyl- | *M. paniculata* | C_11_H_16_ | 148.2 |  | Hydrodistillation | Leaves | Chowdhury et al. (2008) |
| 582 | *cis*-3-Hexenyl valerate | *M. paniculata* | C_11_H_20_O_2_ | 184.3 | 5367682 | Hydrodistillation | Leaves | Chowdhury et al. (2008) |
| 583 | (+)-carvone | *M. paniculata* | C_10_H_14_O | 150.2 | 16724 | Hydrodistillation | Leaves | Chowdhury et al. (2008) |
| 584 | 1H-Imidazole-4-methanol, 5-methyl- | *M. paniculata* | C_5_H_8_N_2_O | 112.1 | 122433 | Hydrodistillation | Leaves | Chowdhury et al. (2008) |
| 585 | Cyclooctene, 4-methylene-6- (1-propenylidene)- | *M. paniculata* | C_12_H_16_ | 160.3 | 5368213 | Hydrodistillation | Leaves | Chowdhury et al. (2008) |
| 586 | Retinal | *M. paniculata* | C_20_H_28_O | 284.4 | 638015 | Hydrodistillation | Leaves | Chowdhury et al. (2008) |
| 587 | 3,9-Dodecadiene | *M. paniculata* | C_12_H_22_ | 166.3 | 5365588 | Hydrodistillation | Leaves | Chowdhury et al. (2008) |
| 588 | 3-Tetradecynoic acid | *M. paniculata* | C_14_H_24_O_2_ | 224.3 | 534441 | Hydrodistillation | Leaves | Chowdhury et al. (2008) |
| 589 | *β*-Vatirenene | *M. paniculata* | C_15_H_22_ | 202.3 | 608753 | Hydrodistillation | Leaves | Chowdhury et al. (2008) |
| 590 | D-Verbenone | *M. paniculata* | C_10_H_14_O | 150.2 | 29025 | Hydrodistillation | Leaves | Chowdhury et al. (2008) |
| 591 | 12-Oxabicyclo (9.1.0) dodeca-3,7-diene, 1,5,5,8-tetramethyl- | *M. paniculata* | C_15_H_24_O | 220.4 | 524129 | Hydrodistillation | Leaves | Chowdhury et al. (2008) |
| 592 | Eremophilene | *M. paniculata* | C_15_H_24_ | 204.4 | 12309744 | Hydrodistillation | Leaves | Chowdhury et al. (2008) |
| 593 | Aromadendrene oxide | *M. paniculata* | C_15_H_24_O | 220.4 | 528759 | Hydrodistillation | Leaves | Chowdhury et al. (2008) |
| 594 | Carveol | *M. paniculata* | C_10_H_16_O | 152.2 | 7438 | Hydrodistillation | Leaves | Chowdhury et al. (2008) |
| 595 | Longifolenealdehyde | *M. paniculata* | C_15_H_24_O | 220.4 | 565584 | Hydrodistillation | Leaves | Chowdhury et al. (2008) |
| 596 | 11-Hexadecyn-1-ol | *M. paniculata* | C_16_H_30_O | 238.4 | 144130 | Hydrodistillation | Leaves | Chowdhury et al. (2008) |
| 597 | 1-Cyclohexene-1-ethanol, 2,6,6-trimethyl- | *M. paniculata* | C_11_H_20_O | 168.3 | 592706 | Hydrodistillation | Leaves | Chowdhury et al. (2008) |
| 598 | Corymbolone | *M. paniculata* | C_15_H_24_O_2_ | 236.4 | 178931 | Hydrodistillation | Leaves | Chowdhury et al. (2008) |
| 599 | Ledene alcohol | *M. paniculata* | C_15_H_24_O | 220.4 | 540567 | Hydrodistillation | Leaves | Chowdhury et al. (2008) |
| 600 | Longifolene-[12]-epoxide | *M. paniculata* | C_15_H_24_O | 220.4 |  | Hydrodistillation | Leaves | Chowdhury et al. (2008) |
| 601 | 2(1H)- Naphthalenone, 4a,5,6,7,8,8a- hexahydro,4a,8a-dimethyl- | *M. paniculata* | C_12_H_18_O | 178.3 | 91700520 | Hydrodistillation | Leaves | Chowdhury et al. (2008) |
| 602 | Muurola-4(14),5-diene | *M. paniculata* | C_15_H_24_ | 204.4 | 91748903 | Pentene | Flowers | Rout et al. (2007) |
| 603 | Patchouli alcohol | *M. paniculata* | C_15_H_26_O | 222.4 | 10955174 | Pentene | Flowers | Rout et al. (2007) |
| 604 | (*Z*, *E*)-Farnesol | *M. exotica* | [C_15_H_26_](https://pubchem.ncbi.nlm.nih.gov/#query=C21H44)O | 222.4 | 1549108 | Hydrodistillation | Leaves, flowers | Raina, et al. (2006) |
| 605 | *trans*-*β*-Guaiene | *M. exotica* | [C_15_H_24_](https://pubchem.ncbi.nlm.nih.gov/#query=C21H44) | 204.4 | 15560252 | Hydrodistillation | Leaves, twigs | Lv et al. (2013) |
| 606 | Cedrene | *M. exotica* | [C_15_H_24_](https://pubchem.ncbi.nlm.nih.gov/#query=C21H44) | 204.4 | 521207 | Hydrodistillation | Fresh leaves | Huang et al. (2013) |
| 607 | *α*-Terpinene | *M. exotica* | C_10_H_16_ | 136.2 | 7462 | Hydrodistillation | Leaves | Krishnamoorthy et al. (2015) |
| 608 | *α*-Muurolene | *M. exotica* | [C_15_H_24_](https://pubchem.ncbi.nlm.nih.gov/#query=C21H44) | 204.4 | 12306047 | Hydrodistillation | Leaves | Krishnamoorthy et al. (2015) |
| 609 | *α*-Thujenal | *M. exotica* | C_10_H_14_O | 150.2 | 530411 | Hydrodistillation | Leaves | Krishnamoorthy et al. (2015) |
| 610 | *α*-Amorphene | *M. exotica* | [C_15_H_24_](https://pubchem.ncbi.nlm.nih.gov/#query=C21H44) | 204.4 | 12306046 | Hydrodistillation | Leaves | Krishnamoorthy et al. (2015) |
| 611 | Thujopsene | *M. exotica* | [C_15_H_24_](https://pubchem.ncbi.nlm.nih.gov/#query=C21H44) | 204.4 | 442402 | Hydrodistillation | Leaves | Krishnamoorthy et al. (2015) |
| 612 | Pentadecanal | *M. exotica* | [C_15_H_30_](https://pubchem.ncbi.nlm.nih.gov/#query=C21H44)O | 226.4 | 17697 | Hydrodistillation | Leaves | Krishnamoorthy et al. (2015) |
| 613 | 3-Octen-1-ol, (*E*)- | *M. exotica* | [C_8_H_16_](https://pubchem.ncbi.nlm.nih.gov/#query=C21H44)O | 128.2 | 5364475 | Hydrodistillation | Leaves | Krishnamoorthy et al. (2015) |
| 614 | Nerol | *M. exotica* | [C_10_H_1_](https://pubchem.ncbi.nlm.nih.gov/#query=C21H44)_8_O | 154.3 | 643820 | n-Hexane | Flowers | Naseem et al. (2015) |
| 615 | 1*R*-*α*-Pinene | *M. exotica* | C_10_H_16_ | 136.2 | 82227 | Hydrodistillation | Leaves | Huang et al. (2013) |
| 616 | *R*(-)3,7-Dimethyl-1,6-octadiene | *M. exotica* | C_10_H_18_ | 138.3 | 10997105 | Hydrodistillation | Leaves | Huang et al. (2013) |
| 617 | Eucalyptol; 1,8-cineole | *M. exotica* | C_10_H_18_O | 154.3 | 2758 | Hydrodistillation | Leaves | Huang et al. (2013) |
| 618 | Octahydro-7-methyl-3-methylene-4-(1-methylethyl)-1H-cyclopenta[1,3] cyclopropa[1,2]benzene | *M. exotica* | C_14_H_22_ | 190.3 | 91752851 | Hydrodistillation | Leaves | Huang et al. (2013) |
| 619 | 2,6-Dimethyl-6-(4-methyl-3-pentenyl)-bicyclo[3.1.1]hept-2-ene; *α*-Bergamotene | *M. exotica* | [C_15_H_24_](https://pubchem.ncbi.nlm.nih.gov/#query=C21H44) | 204.4 | 86608 | Hydrodistillation | Leaves | Huang et al. (2013) |
| 620 | (1*S*-Endo)-2-methyl-3-methylene-2-(4-methyl-3-pentenyl)-bicyclo[2.2.1] heptane | *M. exotica* | [C_15_H_24_](https://pubchem.ncbi.nlm.nih.gov/#query=C21H44) | 204.4 |  | Hydrodistillation | Leaves | Huang et al. (2013) |
| 621 | 2,4α,5,6,7,8,9,9a-Octahydro-3,5,5-trimethyl  -9-methylene-1H-benzocyclo-heptene | *M. exotica* | [C_15_H_24_](https://pubchem.ncbi.nlm.nih.gov/#query=C21H44) | 204.4 |  | Hydrodistillation | Leaves | Huang et al. (2013) |
| 622 | 1-Ethenyl-1-methyl-2-(1-methylethenyl)-4-(1-methylethylidene)-cyclohexane | *M. exotica* | [C_15_H_24_](https://pubchem.ncbi.nlm.nih.gov/#query=C21H44) | 204.4 | 12309452 | Hydrodistillation | Leaves | Huang et al. (2013) |
| 623 | (*Z*)- 5-(1-Propenyl)-1,3-benzodioxole | *M. exotica* | [C_10_H_10_O_2_](https://pubchem.ncbi.nlm.nih.gov/#query=C21H44) | 162.2 | 1549044 | Hydrodistillation | Leaves | Huang et al. (2013) |
| 624 | *Z*-*α*-*trans* -Bergamotol | *M. exotica* | [C_15_H_24_](https://pubchem.ncbi.nlm.nih.gov/#query=C21H44) | 204.4 | 5368743 | Hydrodistillation | Leaves | Huang et al. (2013) |
| 625 | *trans* -*Z*-*α*-Bisabolene epoxide | *M. exotica* | [C_15_H_24_](https://pubchem.ncbi.nlm.nih.gov/#query=C21H44)O | 220.4 | 91753504 | Hydrodistillation | Leaves | Huang et al. (2013) |
| 626 | 1,2-Dimethoxy-4-(2-propenyl)-benzene | *M. exotica* | [C_11_H_14_](https://pubchem.ncbi.nlm.nih.gov/#query=C21H44)O_2_ | 178.2 | 7127 | Hydrodistillation | Leaves | Huang et al. (2013) |
| 627 | Decahydro-1,1,7-trimethyl-4-methylene-1H-cycloprop[e]azulen-7-ol | *M. exotica* | [C_15_H_24_](https://pubchem.ncbi.nlm.nih.gov/#query=C21H44)O | 220.4 | 522266 | Hydrodistillation | Leaves | Huang et al. (2013) |
| 628 | *trans*-Sabinene hydrate | *M. exotica* | [C_10_H_18_O](https://pubchem.ncbi.nlm.nih.gov/#query=C21H44) | 154.3 | 12315151 | Hydrodistillation | Leaves, twigs | Lv et al. (2013) |
| 629 | *cis*-Sabinene hydrate | *M. exotica* | [C_10_H_18_O](https://pubchem.ncbi.nlm.nih.gov/#query=C21H44) | 154.3 | 62367 | Hydrodistillation | Leaves, flowers | Raina et al. (2006) |
| 630 | *γ*-Terpineol | *M. exotica* | C_10_H_18_O | 154.3 | 11467 | Hydrodistillation | Leaves, twigs | Lv et al. (2013) |
| 631 | Neral | *M. exotica* | C_10_H_16_O | 152.2 | 643779 | Hydrodistillation | Leaves, twigs | Lv et al. (2013) |
| 632 | Piperitone | *M. exotica* | C_10_H_16_O | 152.2 | 6987 | Hydrodistillation | Leaves, twigs | Lv et al. (2013) |
| 633 | Geranial | *M. exotica* | C_10_H_16_O | 152.2 | 638011 | Hydrodistillation | Leaves, twigs | Lv et al. (2013) |
| 634 | 2,3,4-Trimethyl benzaldehyde | *M. exotica* | C_10_H_12_O | 148.2 | 2752597 | Hydrodistillation | Leaves, twigs | Lv et al. (2013) |
| 635 | Linalool propanoate | *M. exotica* | C_14_H_24_O_2_ | 224.3 | 6431132 | Hydrodistillation | Leaves, twigs | Lv et al. (2013) |
| 636 | Neryl acetate | *M. exotica* | C_12_H_20_O_2_ | 196.3 | 1549025 | Hydrodistillation | Leaves, twigs | Lv et al. (2013) |
| 637 | *E*-*β*-Damascenone | *M. exotica* | [C_13_H_18_O](https://pubchem.ncbi.nlm.nih.gov/#query=C21H44) | 190.3 | 5366074 | Hydrodistillation | Leaves, twigs | Lv et al. (2013) |
| 638 | *trans*-Muurola-3,5-diene | *M. exotica* | [C_15_H_24_](https://pubchem.ncbi.nlm.nih.gov/#query=C21H44) | 204.4 | 10632031 | Hydrodistillation | Leaves, twigs | Lv et al. (2013) |
| 639 | *trans*-Calamenene | *M. exotica* | [C_15_H_22_](https://pubchem.ncbi.nlm.nih.gov/#query=C21H44) | 202.4 | 6429022 | Hydrodistillation | Leaves, twigs | Lv et al. (2013) |
| 640 | *β*-Chamigrene | *M. exotica* | [C_15_H_24_](https://pubchem.ncbi.nlm.nih.gov/#query=C21H44) | 204.4 | 442353 | Hydrodistillation | Leaves, twigs | Lv et al. (2013) |
| 641 | Zonarene | *M. exotica* | [C_15_H_24_](https://pubchem.ncbi.nlm.nih.gov/#query=C21H44) | 204.4 | 6428488 | Hydrodistillation | Leaves, twigs | Lv et al. (2013) |
| 642 | *epi*-Zizanone | *M. exotica* | [C_15_H_22_](https://pubchem.ncbi.nlm.nih.gov/#query=C21H44)O | 218.3 | 101618762 | Hydrodistillation | Leaves, twigs | Lv et al. (2013) |
| 643 | 3*E*-Cembrene A | *M. exotica* | [C_20_H_32_](https://pubchem.ncbi.nlm.nih.gov/#query=C21H44) | 272.5 |  | Hydrodistillation | Leaves, twigs | Lv et al. (2013) |
| 644 | 1,10-Epoxy-amorph-4-ene | *M. exotica* | [C_15_H_24_](https://pubchem.ncbi.nlm.nih.gov/#query=C21H44)O | 220.4 | 6428329 | Hydrodistillation | Leaves, twigs | Lv et al. (2013) |
| 645 | n-Octadecanol | *M. exotica* | [C_18_H_38_](https://pubchem.ncbi.nlm.nih.gov/#query=C21H44)O | 270.5 | 8221 | Hydrodistillation | Leaves, twigs | Lv et al. (2013) |
| 646 | *E*-phytol acetate | *M. exotica* | [C_22_H_42_](https://pubchem.ncbi.nlm.nih.gov/#query=C21H44)O_2_ | 338.6 | 6428538 | Hydrodistillation | Leaves, twigs | Lv et al. (2013) |
| 647 | *β*-Copaen-4-*α*-ol | *M. exotica* | [C_15_H_24_](https://pubchem.ncbi.nlm.nih.gov/#query=C21H44)O | 220.4 | 91748521 | Hydrodistillation | Leaves, twigs | Lv et al. (2013) |
| 648 | Salvial-4(14)-en-1-one | *M. exotica* | [C_15_H_24_](https://pubchem.ncbi.nlm.nih.gov/#query=C21H44)O | 220.4 | 42608172 | Hydrodistillation | Leaves, twigs | Lv et al. (2013) |
| 649 | Allo-aromadendrene epoxide | *M. exotica* | [C_15_H_24_](https://pubchem.ncbi.nlm.nih.gov/#query=C21H44)O | 220.4 | 91746712 | Hydrodistillation | Leaves, twigs | Lv et al. (2013) |
| 650 | 10-Nor-calamenen-10-one | *M. exotica* | [C_14_H_18_](https://pubchem.ncbi.nlm.nih.gov/#query=C21H44)O | 202.3 | 6429078 | Hydrodistillation | Leaves, twigs | Lv et al. (2013) |
| 651 | *β*-Bisabolenol | *M. exotica* | [C_15_H_24_](https://pubchem.ncbi.nlm.nih.gov/#query=C21H44)O | 220.4 | 91747530 | Hydrodistillation | Leaves, twigs | Lv et al. (2013) |
| 652 | *n*-Octadecane β | *M. exotica* | [C_18_H_38_](https://pubchem.ncbi.nlm.nih.gov/#query=C21H44)O | 254.5 | 11635 | Hydrodistillation | Leaves, twigs | Lv et al. (2013) |
| 653 | *β*-Vetivone | *M. exotica* | [C_15_H_22_](https://pubchem.ncbi.nlm.nih.gov/#query=C21H44)O | 218.3 | 442406 | Hydrodistillation | Leaves, twigs | Lv et al. (2013) |
| 654 | *n*-Nonadecane | *M. exotica* | [C_19_H_40_](https://pubchem.ncbi.nlm.nih.gov/#query=C21H44) | 268.5 | 12401 | Hydrodistillation | Leaves, twigs | Lv et al. (2013) |
| 655 | *α*-Terpineol | *M. exotica* | [C_10_H_18_O](https://pubchem.ncbi.nlm.nih.gov/#query=C21H44) | 154.3 | 442501 | Hydrodistillation | Aerial parts | Li et al. (2010) |
| 656 | Thymol methyl ether | *M. exotica* | [C_11_H_16_O](https://pubchem.ncbi.nlm.nih.gov/#query=C21H44) | 164.2 | 14104 | Hydrodistillation | Aerial parts | Li et al. (2010) |
| 657 | *p*-Menth-1(7)-en-2-one | *M. exotica* | [C_10_H_16_O](https://pubchem.ncbi.nlm.nih.gov/#query=C21H44) | 152.2 | 557612 | Hydrodistillation | Aerial parts | Li et al. (2010) |
| 658 | Phellandral | *M. exotica* | [C_10_H_16_O](https://pubchem.ncbi.nlm.nih.gov/#query=C21H44) | 152.2 | 89488 | Hydrodistillation | Aerial parts | Li et al. (2010) |
| 659 | Bornyl acetate | *M. exotica* | [C_12_H_20_O](https://pubchem.ncbi.nlm.nih.gov/#query=C21H44)_3_ | 196.3 | 93009 | Hydrodistillation | Aerial parts | Li et al. (2010) |
| 670 | Thymol | *M. exotica* | [C_10_H_14_O](https://pubchem.ncbi.nlm.nih.gov/#query=C21H44) | 150.2 | 6989 | Hydrodistillation | Aerial parts | Li et al. (2010) |
| 671 | 4-Vinylguaiacol | *M. exotica* | [C_10_H_14_O](https://pubchem.ncbi.nlm.nih.gov/#query=C21H44) | 150.2 | 332 | Hydrodistillation | Aerial parts | Li et al. (2010) |
| 672 | Eugenol | *M. exotica* | [C_10_H_12_O](https://pubchem.ncbi.nlm.nih.gov/#query=C21H44)_2_ | 164.2 | 3314 | Hydrodistillation | Aerial parts | Li et al. (2010) |
| 673 | *β*-Patchoulene | *M. exotica* | [C_15_H_24_](https://pubchem.ncbi.nlm.nih.gov/#query=C21H44) | 204.4 | 101731 | Hydrodistillation | Aerial parts | Li et al. (2010) |
| 674 | Eudesma-3,7(11)-diene | *M. exotica* | [C_15_H_24_](https://pubchem.ncbi.nlm.nih.gov/#query=C21H44) | 204.4 | 6432648 | Hydrodistillation | Aerial parts | Li et al. (2010) |
| 675 | *α*-Calacorene | *M. exotica* | [C_15_H_20_](https://pubchem.ncbi.nlm.nih.gov/#query=C21H44) | 200.3 | 528708 | Hydrodistillation | Aerial parts | Li et al. (2010) |
| 676 | *cis*-Nerolidol | *M. exotica* | [C_15_H_26_](https://pubchem.ncbi.nlm.nih.gov/#query=C21H44)O | 222.4 | 5320128 | Hydrodistillation | Aerial parts | Li et al. (2010) |
| 677 | Camphene | *M. exotica* | C_10_H_16_ | 136.2 | 6616 | Hydrodistillation | Leaves, flowers | Raina et al. (2006) |
| 678 | *β*-Pinene | *M. exotica* | C_10_H_16_ | 136.2 | 14896 | Hydrodistillation | Leaves, flowers | Raina et al. (2006) |
| 679 | Lavandulol | *M. exotica* | C_10_H_18_O | 154.3 | 5464156 | Hydrodistillation | Leaves, flowers | Raina et al. (2006) |
| 680 | Borneol | *M. exotica* | C_10_H_18_O | 154.3 | 64685 | Hydrodistillation | Leaves, flowers | Raina et al. (2006) |
| 681 | Neryl formate | *M. exotica* | C_11_H_18_O_2_ | 182.3 | 5354882 | Hydrodistillation | Leaves, flowers | Raina et al. (2006) |
| 682 | Lavandulyl acetate | *M. exotica* | C_12_H_20_O_2_ | 196.3 | 30247 | Hydrodistillation | Leaves, flowers | Raina et al. (2006) |
| 683 | Tridecanoic acid | *M. exotica* | C_13_H_26_O_2_ | 214.3 | 12530 | Hydrodistillation | Leaves, flowers | Raina et al. (2006) |
| 684 | Tetradecanol | *M. exotica* | C_14_H_30_O | 214.3 | 8209 | Hydrodistillation | Leaves, flowers | Raina et al. (2006) |
| 685 | *γ*-Methyl ionone | *M. exotica* | C_14_H_22_O | 206.3 | 5356787 | Hydrodistillation | Leaves, flowers | Raina et al. (2006) |
| 686 | Pentadecan-2-one | *M. exotica* | C_15_H_30_O | 226.3 | 61303 | Hydrodistillation | Leaves, flowers | Raina et al. (2006) |
| 687 | Hexadecanal | *M. exotica* | C_16_H_32_O | 240.4 | 984 | Hydrodistillation | Leaves, flowers | Raina et al. (2006) |
| 688 | Isopropyl myristate | *M. exotica* | C_17_H_34_O_2_ | 270.5 | 8042 | Hydrodistillation | Leaves, flowers | Raina et al. (2006) |
| 689 | Hexadecanol | *M. exotica* | C_16_H_34_O | 242.4 | 2682 | Hydrodistillation | Leaves, flowers | Raina et al. (2006) |
| 690 | Pentadecanoic acid | *M. exotica* | C_15_H_30_O_2_ | 242.4 | 13849 | Hydrodistillation | Leaves, flowers | Raina et al. (2006) |
| 691 | Oleic acid | *M. exotica* | C_18_H_34_O_2_ | 282.5 | 445639 | Hydrodistillation | Leaves, flowers | Raina et al. (2006) |
| 692 | (*E,E,E*)-*α-*Springene | *M. exotica* | C_20_H_32_ | 272.5 | 5365883 | Hydrodistillation | Leaves, flowers | Raina et al. (2006) |
| 693 | Eicosanal | *M. exotica* | C_20_H_40_O | 296.5 | 75458 | Hydrodistillation | Leaves, flowers | Raina et al. (2006) |
| 694 | Methyl pimarate | *M. exotica* | C_21_H_32_O_2_ | 316.5 | 11023516 | Hydrodistillation | Leaves, flowers | Raina et al. (2006) |
| 695 | Phenylethyl anthranilate | *M. exotica* | C_15_H_15_NO_2_ | 241.3 | 8615 | Hydrodistillation | Leaves, flowers | Raina et al. (2006) |
| 696 | Ethylbutyrate | *M. exotica* | C_6_H_12_O_2_ | 116.2 | 7762 | Hydrodistillation | Leaves | Pino et al. (2006) |
| 697 | (*Z*)-3-Hexenol | *M. exotica* | C_6_H_12_O | 100.2 | 5281167 | Hydrodistillation | Leaves | Pino et al. (2006) |
| 698 | Hexanol | *M. exotica* | C_6_H_14_O | 102.2 | 8103 | Hydrodistillation | Leaves | Pino et al. (2006) |
| 699 | Heptanal | *M. exotica* | C_7_H_14_O | 114.2 | 8130 | Hydrodistillation | Leaves | Pino et al. (2006) |
| 700 | Mesitylene | *M. exotica* | C_9_H_12_ | 120.2 | 7947 | Hydrodistillation | Leaves | Pino et al. (2006) |
| 701 | Nonanal | *M. exotica* | C_9_H_18_O | 142.2 | 31289 | Hydrodistillation | Leaves | Pino et al. (2006) |
| 702 | *p*-Mentha-1,3,8-triene | *M. exotica* | C_10_H_14_ | 134.2 | 176983 | Hydrodistillation | Leaves | Pino et al. (2006) |
| 703 | Isophorone | *M. exotica* | C_9_H_18_O | 138.2 | 6544 | Hydrodistillation | Leaves | Pino et al. (2006) |
| 704 | (*Z*)-3-Hexenyl isovalerate | *M. exotica* | C_11_H_20_O_2_ | 184.3 | 5367681 | Hydrodistillation | Leaves | Pino et al. (2006) |
| 705 | *cis*-Muurola-4(14),5-diene | *M. exotica* | C_15_H_24_ | 204.4 | 51351709 | Hydrodistillation | Leaves | Pino et al. (2006) |
| 706 | Valerianol | *M. exotica* | C_15_H_26_O | 222.4 | 9859337 | Hydrodistillation | Leaves | Pino et al. (2006) |
| 707 | Isobornyl acetate | *M. exotica* | C_12_H_20_O_2_ | 196.3 | 6950273 | Hydrodistillation | Leaves, fruits, flowers | El-Sakhawy et al. (1998) |
| 708 | Cuparene | *M. exotica* | C_15_H_22_ | 202.4 | 86895 | Hydrodistillation | Leaves, fruits, flowers | El-Sakhawy et al. (1998) |
| 709 | *α*-Longipinene | *M. exotica* | C_15_H_24_ | 204.4 | 520957 | Hydrodistillation | Leaves, fruits, flowers | El-Sakhawy et al. (1998) |
| 710 | Gamma-Methylionone | *M. exotica* | C_14_H_22_O | 206.3 | 6365590 | Hydrodistillation | Leaves, fruits, flowers | El-Sakhawy et al. (1998) |
| 711 | Naphthalene | *M. exotica* | C_10_H_8_ | 128.2 | 931 | Hydrodistillation | Leaves, fruits, flowers | El-Sakhawy et al. (1998) |
| 712 | Tricyclene | *M. exotica* | C_10_H_16_ | 136.2 | 79035 | Hydrodistillation | Leaves, fruits, flowers | El-Sakhawy et al. (1998) |
| 713 | *o*-Cymene | *M. exotica* | C_10_H_14_ | 134.2 | 10703 | Hydrodistillation | Leaves, fruits, flowers | El-Sakhawy et al. (1998) |
| 714 | *E-*Carophyllene | *M. exotica* | [C_15_H_24_](https://pubchem.ncbi.nlm.nih.gov/#query=C21H44) | 204.4 | 5281522 | n-Hexane | Flowers | Naseem et al. (2015) |
| 715 | Benzyl salicylate | *M. exotica* | [C_14_H_12_O_3_](https://pubchem.ncbi.nlm.nih.gov/#query=C21H44) | 228.2 | 8363 | n-Hexane | Flowers | Naseem et al. (2015) |
| 716 | *n*-Eicosane | *M. exotica* | [C_14_H_12_O_3_](https://pubchem.ncbi.nlm.nih.gov/#query=C21H44) | 228.2 | 8222 | n-Hexane | Flowers | Naseem et al. (2015) |
| 717 | *γ*-Pyronene | *M. exotica* | [C_10_H_16_](https://pubchem.ncbi.nlm.nih.gov/#query=C21H44) | 136.2 | 578237 | Hydrodistillation | Branches with leaves | You et al. (2015) |
| 718 | Calarene | *M. exotica* | [C_15_H_24_](https://pubchem.ncbi.nlm.nih.gov/#query=C21H44) | 204.4 | 28481 | Hydrodistillation | Branches with leaves | You et al. (2015) |
| 719 | *γ*-Selinene | *M. exotica* | [C_15_H_24_](https://pubchem.ncbi.nlm.nih.gov/#query=C21H44) | 204.4 | 521334 | Hydrodistillation | Branches with leaves | You et al. (2015) |
| 720 | 10-*epi*-*γ*-Eudesmol | *M. exotica* | C_15_H_26_O | 222.4 | 6430754 | Hydrodistillation | Leaves, flowers | Raina et al. (2006) |

Note: — refer to “Not mention”
